# MOGAT3-mediated DAG accumulation drives acquired resistance to anti-BRAF/anti-EGFR therapy in *BRAF^V600E^*-mutant metastatic colorectal cancer

**DOI:** 10.1172/JCI182217

**Published:** 2024-10-22

**Authors:** Jiawei Wang, Huogang Wang, Wei Zhou, Xin Luo, Huijuan Wang, Qing Meng, Jiaxin Chen, Xiaoyu Chen, Yingqiang Liu, David W. Chan, Zhenyu Ju, Zhangfa Song

**Affiliations:** 1 Department of Colorectal Surgery, Sir Run Run Shaw Hospital, School of Medicine, Zhejiang University, Hangzhou, Zhejiang, China.; 2 Key Laboratory of Biological Treatment of Zhejiang Province, Hangzhou, China.; 3 Key Laboratory of Integrated Traditional Chinese and Western Medicine Research on Anorectal Diseases of Zhejiang Province, Hangzhou, China.; 4 School of Medicine, The Chinese University of Hong Kong, Shenzhen, Guangdong, China.; 5 Key Laboratory of Regenerative Medicine of Ministry of Education, Institute of Aging and Regenerative Medicine, Jinan University, Guangzhou, China.

**Keywords:** Gastroenterology, Therapeutics, Colorectal cancer, Drug therapy

## Abstract

*BRAF^V600E^*-mutant metastatic colorectal cancer (mCRC) is associated with poor prognosis. The combination of anti-BRAF/anti-EGFR (encorafenib/cetuximab) treatment for patients with *BRAF^V600E^*-mutant mCRC improves clinical benefits; unfortunately, inevitable acquired resistance limits the treatment outcome, and the mechanism has not been validated. Here, we discovered that monoacylglycerol O-acyltransferase 3–mediated (MOGAT3-mediated) diacylglycerol (DAG) accumulation contributed to acquired resistance to encorafenib/cetuximab by dissecting a *BRAF^V600E^*-mutant mCRC patient–derived xenograft (PDX) model exposed to encorafenib/cetuximab administration. Mechanistically, the upregulated MOGAT3 promoted DAG synthesis and reduced fatty acid oxidation–promoting DAG accumulation and activated PKCα/CRAF/MEK/ERK signaling, driving acquired resistance. Resistance-induced hypoxia promoted MOGAT3 transcriptional elevation; simultaneously, MOGAT3-mediated DAG accumulation increased HIF1A expression at the translation level through PKCα/CRAF/eIF4E activation, strengthening the resistance status. Intriguingly, reducing intratumoral DAG with fenofibrate or PF-06471553 restored the antitumor efficacy of encorafenib/cetuximab in resistant *BRAF^V600E^*-mutant mCRC, which interrupted PKCα/CRAF/MEK/ERK signaling. These findings reveal the critical role of the metabolite DAG as a modulator of encorafenib/cetuximab efficacy in *BRAF^V600E^*-mutant mCRC, suggesting that fenofibrate might prove beneficial for resistant *BRAF^V600E^*-mutant mCRC patients.

## Abstract 2

MOGAT3-mediated DAG as a modulator of encorafenib/cetuximab efficacy in *BRAF^V600^*^E^-mutant mCRC, and fenofibrate inhibiting DAG improve the treatment efficiency of encorafenib/cetuximab in resistant* BRAF^V600E^*-mutant mCRC.

## Graphical abstract

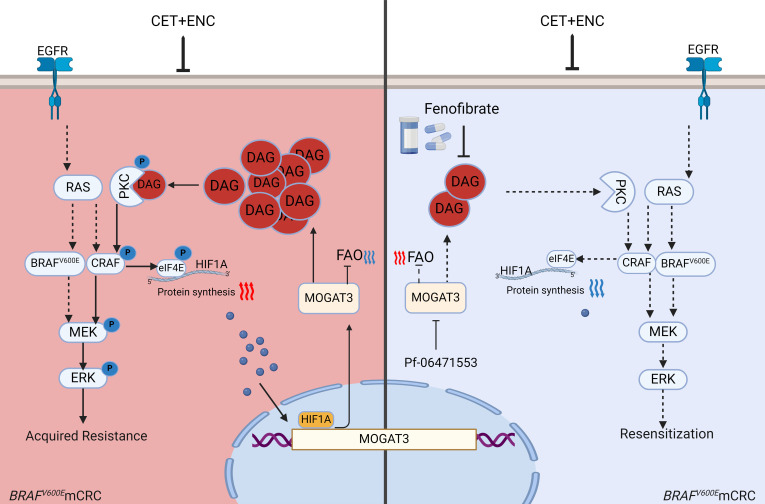

## Introduction

Colorectal cancer (CRC), the second-leading cause of cancer-related mortality worldwide, is a highly heterogeneous cancer with multiple genetic subtypes (1). Ten percent of CRC patients are diagnosed with mutations in the *BRAF* oncogene of the MAPK pathway, and the most common missense mutation occurs at the 600th amino acid with a valine to glutamic acid (V600E) change, predicting distant metastasis and poor prognosis (2). Unfortunately, patients diagnosed with metastatic CRC (mCRC) harboring the *BRAF^V600E^* mutation respond poorly to conventional chemotherapy (3). Recently, anti-BRAF/anti-EGFR (hereafter referred to as anti-BRAF/EGFR) combinatorial therapy (encorafenib/cetuximab) was approved in April, 2020 by the US Food and Drug Administration (FDA) for the treatment of patients with *BRAF^V600E^*-mutant mCRC (4). Despite the favorable initial response of this therapy, almost all the *BRAF^V600E^*-mutant mCRC patients developed therapy resistance after approximately 4.3 months of treatment (5). Moreover, the objective response rate was only 28.0%, and the median overall survival was 9.57 months (6). Improving the efficiency of encorafenib/cetuximab to control disease progression in *BRAF^V600E^*-mutant mCRC remains challenging.

Cotargeting BRAF/EGFR reinforces inhibition of the oncogenic BRAF/MEK pathway while shutting down the adverse feedback resistance pathway (EGFR), the theoretical basis of dual-target therapy. A recent study showed that SRC kinases are systematically activated in *BRAF^V600E^*-mutant mCRC following targeted inhibition of BRAF with and without EGFR inhibition (7). Clinical observation showed that 43% of *BRAF^V600E^*-mutant mCRC patients treated with anti-BRAF/EGFR acquired an *RNF43* mutation related to treatment failure (8). Continuous treatment often drives genomic alteration or epigenetic changes, ultimately leading to resistance. However, the acquired resistance mechanism of *BRAF^V600E^*-mutant mCRC to ongoing encorafenib/cetuximab treatment is largely unknown.

Metabolic adaptation confers tumor survival in a harsh drug-exposure environment (9), especially in *BRAF^V600E^*-mutant tumors (10). A preclinical model showed that *BRAF^V600E^*-mutant tumors determine lipid profiles in response to drug treatment (11). In addition to fueling tumor cells, lipids orchestrate signal transduction cascades to support tumor growth upon harsh drug treatment. Moreover, increasing evidence has indicated that aberrant lipid droplet accumulation in CRC with *KRAS* and *BRAF* mutations is associated with a poor response to anti-EGFR therapy (erlotinib), implying drug-resistant status in *BRAF*-mutated tumors is closely related to lipid metabolism (12, 13). As an essential lipid metabolism pathway, glyceride homeostasis maintains various biological processes and functions, including energy supply and signal transduction (14), primarily dependent on monoacylglycerol O-acyltransferase (MOGAT) activity (15). Abnormal MOGAT enzymatic activities (MOGAT1, MOGAT2, and MOGAT3) are associated with various disease progressions, such as nonalcoholic fatty liver disease and obesity (16, 17). Similarly, MOGAT3 is believed to maintain glyceride homeostasis in the human intestine and liver (18); however, the functions of MOGAT3 in physiological processes and tumor progression remain to be clarified.

In this study, we report that the upregulated MOGAT3 endows resistance of *BRAF^V600E^*-mutant mCRC to encorafenib/cetuximab treatment through synthesis of diacylglycerol (DAG), connecting the phosphorylated protein kinase C α (PKCα)/CRAF/MEK/ERK signaling axis. Specifically, resistance-induced hypoxia promotes *MOGAT3* transcriptional elevation and MOGAT3-mediated DAG synthesis and inhibits lipid oxidation respiration, resulting in intratumoral DAG accumulation. Accumulated intratumoral DAG reactivates MAPK signaling circuitry through PKCα/CRAF phosphorylation activation and strengthens HIF1A expression through PKCα/CRAF/eIF4E activation. Of note, targeting MOGAT3 or reducing intratumoral DAG restores the treatment efficiency of anti-BRAF/EGFR combinatorial therapy in resistant *BRAF^V600E^-*mutant mCRC. Overall, our study uncovers a clinically actionable strategy to fix the failure of anti-BRAF/EGFR combinatorial therapies.

## Results

### Upregulated lipid metabolism linked to encorafenib/cetuximab resistance in BRAF^^V600E^^-mutant mCRC.

**Figure 1:**
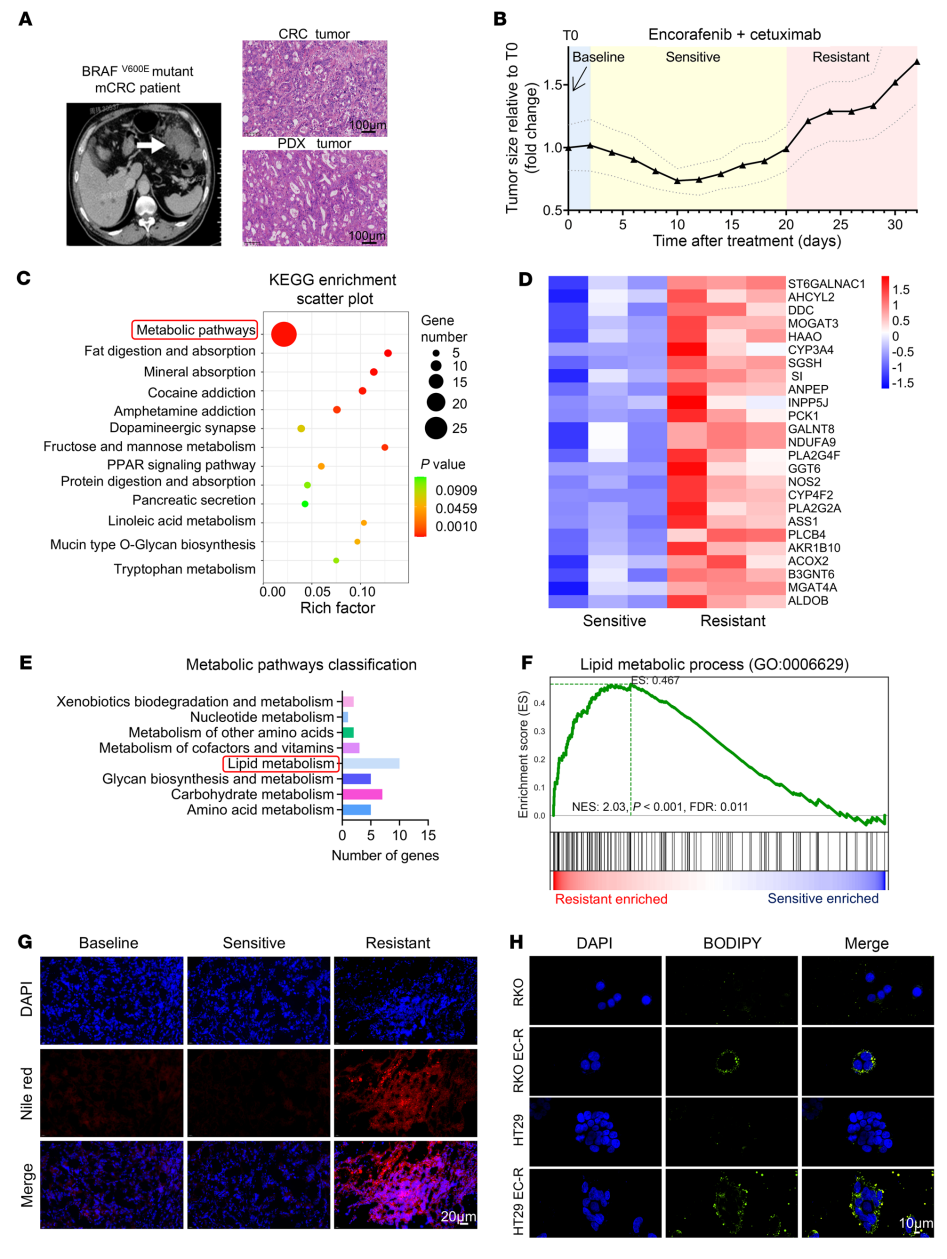
Encorafenib- and cetuximab-resistant *BRAF^V600E^*-mutant mCRC tumors exhibited abnormal lipid-metabolizing activity. (**A**) Patient-derived *BRAF^V600E^*-mutant mCRC samples: Computed tomography picture shows primary tumor location (left) and H&E morphology of original primary and PDX tumor mass (right). (**B**) Mean tumor volumes (±SEM) of *BRAF^V600E^*-mutant mCRC PDXs treated with encorafenib and cetuximab relative to baseline (T0) (*n* = 6). (**C**) Bubble plot showing KEGG pathways (https://www.genome.jp/kegg/pathway.html) of upregulated genes enriched in resistant PDX tumors versus sensitive PDX tumors based on RNA-seq data (*n* = 3). (**D**) Heatmap showing metabolic pathways genes related to **C** (*n* = 3). (**E**) Bar chart presenting a classification of metabolic pathways genes related to **D**. (**F**) Gene set enrichment analysis (GSEA) of resistant tumors versus sensitive tumors (*n* = 3) showing enhanced lipid metabolic process. Normalized enrichment score (NES) and nominal *P* value are provided according to GSEA. (**G**) Lipid droplet content of tumors was assessed by Nile red staining over 3 periods. Representative images are shown from 3 independent experiments. Scale bar: 20 μm. (**H**) RKO, RKO EC-R, HT29, and HT29 EC-R cells were stained with BODIPY 493/503 (green). Representative images are shown from 3 independent experiments. Scale bar: 10 μm.

To investigate the potential mechanism of the acquired resistance of anti-BRAF/EGFR therapy to *BRAF^V600E^*-mutant mCRC, we first employed operative tumor tissue derived from untreated *BRAF^V600E^*-mutant mCRC patients with liver metastasis to establish patient-derived xenograft (PDX) models to thoroughly assess the progressive resistance of encorafenib/cetuximab treatment on *BRAF^V600E^*-mutant mCRC (Figure 1A). The histological assessment showed successful PDX tumor model establishment (Supplemental Figure 1A; supplemental material available online with this article; https://doi.org/10.1172/JCI182217DS1). PDX tumors reaching 150 mm^3^ received either vehicle or drug treatments (20 mg/kg encorafenib orally daily; 20 mg/kg cetuximab i.p. twice weekly), mirroring clinical dosing (19). After continuous dosing, tumors exhibited resistance compared with initial regression (Figure 1B). The response of PDX tumors to encorafenib/cetuximab treatment was categorized into 3 stages based on tumor volume changes: baseline (untreated), sensitive (regression from baseline), and resistant (progression from baseline) (Figure 1B). Moreover, histological analysis of PDX tumors revealed statistically significant increases in Ki67 levels and decreases in TUNEL staining in resistant tumors compared with sensitive ones after 20 days of encorafenib/cetuximab treatment (Supplemental Figure 1A). Notably, resistant PDX tumors recapitulated the response to encorafenib/cetuximab treatment in vivo, confirming the successful establishment of the acquired-resistance PDX model (Supplemental Figure 1C). Sensitive and resistant PDX tumors were then reimplanted in vivo. After 20 days of drug-free growth, both were subjected to encorafenib/cetuximab therapy. The resistant tumors maintained their robust resistance, demonstrating that the resistance in the PDX model is stable and enduring, not merely a transient adaptive response (Supplemental Figure 1D). To characterize the *BRAF^V600E^*-mutant mCRC evolution to anti-BRAF/EGFR therapy resistance, we first performed whole-exome sequencing to analyze the PDX tumors within different response periods (baseline, sensitive, and resistant). The *BRAF^V600E^* mutation was conserved, and no new consistent mutations (e.g., *RNF43*) were detected in resistant tumors compared to baseline and sensitive ones, suggesting that transcriptional differences may underlie the acquired resistance (Supplemental Figure 1B). Next, transcriptomic analysis was performed to compare the PDX tumors in baseline, sensitive, and resistant periods. RNA-seq enrichment analysis identified that the most differentially regulated pathway was the metabolic pathway (Figure 1, C and D), especially the lipid metabolism pathway (Figure 1E), which was significantly upregulated in resistant tumors. Moreover, gene set enrichment analysis (GSEA) revealed that the lipid metabolic process was upregulated in resistant PDX tumors (Figure 1F). Consistently, we observed that the levels of intratumoral lipid (identified by Nile red staining) markedly increased in resistant PDX tumor tissues compared with sensitive PDX tumors (Figure 1G). In addition, we generated 2 encorafenib/cetuximab-resistant human *BRAF^V600E^*-mutant CRC cell lines, RKO EC-R and HT29 EC-R (Supplemental Figure 1E). A similar intracellular lipid increase was observed in resistant cells (identified by BODIPY 493/503 staining), and the *BRAF^V600E^* mutation was consistent in resistant cells and parental cells (Figure 1H and Supplemental Figure 1F). Together, these results suggested lipid metabolism upregulation is associated with the acquired resistance of anti-BRAF/EGFR therapy in *BRAF^V600E^*-mutant mCRC.

### DAG accumulation induces BRAF/EGFR therapy resistance in BRAF^^V600E^^-mutant mCRC.

**Figure 2:**
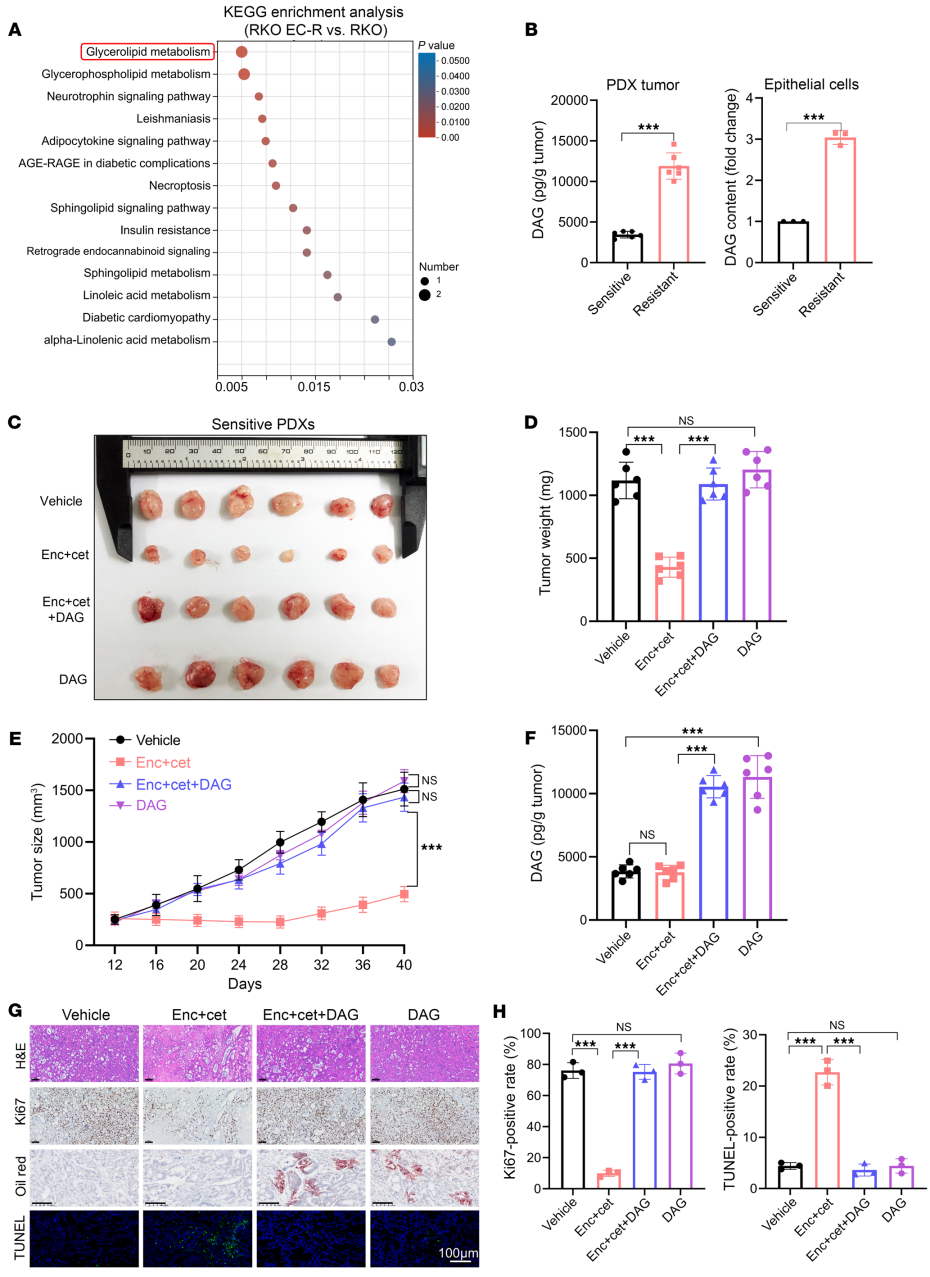
DAG accumulation drives the acquired resistance of *BRAF^V600E^*-mutant mCRC to BRAF/EGFR inhibitor treatment. (**A**) Bubble plot showing KEGG pathways of upregulated metabolites enriched in RKO EC-R versus RKO cells based on lipidomic analysis (*n* = 6). The *x* axis shows the *P* values. (**B**) DAG content in PDX tumors (*n* = 6) and DAG content in tumor epithelial cells (*n* = 3). (**C**–**F**) Xenograft tumor size in nude mice inoculated with encorafenib- and cetuximab-sensitive *BRAF^V600E^*-mutant mCRC tumor tissues (*n* = 6). (**C**) PDXs were treated i.p. with vehicle (PBS), encorafenib-cetuximab, encorafenib-cetuximab combined with DAG, or DAG alone. (**D**) Tumor weight, (**E**) tumor growth, and (**F**) intratumoral DAG level. (**G**) Representative images of H&E, Ki67, Oil Red O, and TUNEL staining related to **C**. Scale bar: 100 μm. (**H**) Ki67 and TUNEL quantitation (*n* = 3). The data are presented as mean ± SEM of 3 independent experiments. NS, no significance. ***P < 0.001 by 2-tailed, unpaired *t* test (**B**), 1-way ANOVA with Tukey’s multiple-comparison test (**D**, **F**, and **H**), or 2-way ANOVA with Tukey’s multiple-comparison test (**E**).

To characterize the underlying lipid biological processes in the resistant *BRAF^V600E^*-mutant mCRC during dual therapy treatment, we performed lipid metabolomics analysis and found that glyceride metabolism was significantly upregulated in resistant *BRAF^V600E^*-mutant mCRC (Figure 2A). Glyceride metabolism cycling is the process of DAG and triacylglycerol (TAG) synthesis and decomposition (20). Indeed, we observed statistically significant increases in intratumoral DAG and TAG levels in resistant PDX tumors compared with sensitive ones (Figure 2B and Supplemental Figure 2, A–C). Furthermore, we sorted EpCAM^+^ tumor cells from resistant and sensitive PDX tumors and discovered that DAG and TAG predominantly originated from these cells (Supplemental Figure 2F). These results suggest that elevated levels of TAG or DAG may contribute to the resistance observed. To test this hypothesis, we first assessed the treatment efficiency of encorafenib (0.25 μM) plus cetuximab (0.5 μM) in sensitive RKO and HT29 cells upon DAG (10 μM) or TAG (10 μM) treatment according to DAG/TAG concentration in resistant tumors. Surprisingly, a significant increase in cell growth and decreased cell apoptosis was observed in the group with encorafenib/cetuximab plus DAG, but not in the TAG group compared with the dual-therapy group (Supplemental Figure 2, D and E). These data demonstrated that DAG, but not TAG, enhances the resistance of *BRAF^V600E^*-mutant mCRC to encorafenib/cetuximab.

To further substantiate DAG-mediated encorafenib/cetuximab resistance, we evaluated the treatment efficiency of encorafenib/cetuximab combined with DAG or vehicle in sensitive PDX tumors (Figure 2C). As expected, DAG (100 mg/kg/day) treatment significantly promoted sensitive PDX tumor growth upon dual-therapy treatment, which was associated with intratumoral DAG elevation (Figure 2, C–F). Moreover, histological assessment of sensitive PDX tumors demonstrated statistically significant increases in the levels of Ki67 and decreased the TUNEL level upon DAG plus encorafenib/cetuximab treatment compared with the dual-therapy group (Figure 2, G and H). These data illustrated that intratumoral DAG accumulation contributes to anti-BRAF/EGFR treatment resistance in *BRAF^V600E^*-mutant mCRC.

### MOGAT3-driven DAG buildup promotes anti-BRAF/EGFR therapy resistance.

**Figure 3:**
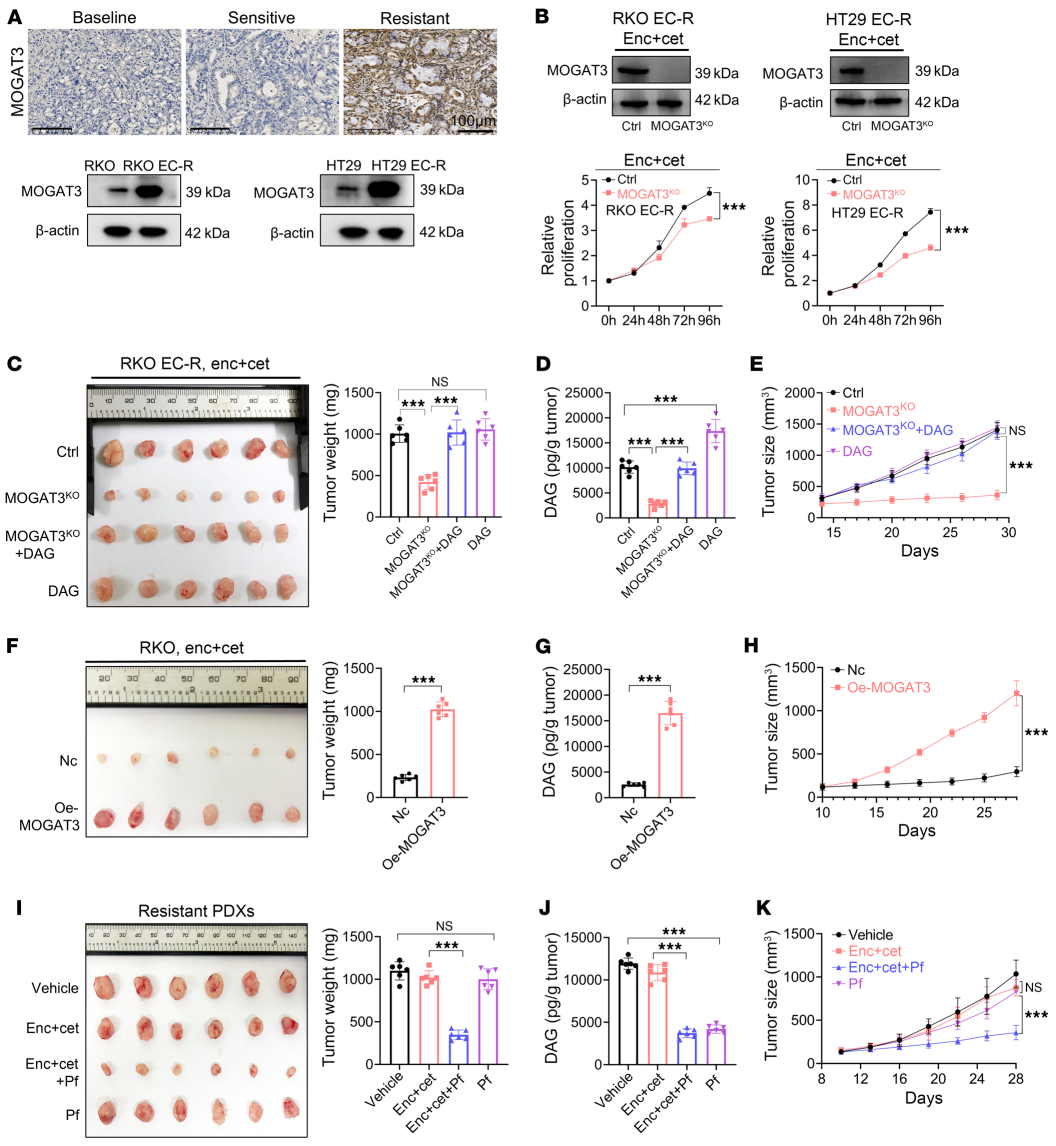
MOGAT3-mediated DAG elevation determines anti-BRAF/EGFR treatment failure in *BRAF^V600E^*-mutant mCRC tumors. (**A**) Representative IHC images of MOGAT3 in baseline, sensitive, and resistant tumor tissues. Scale bar: 100 μm. (**B**) Top: Western blots showing protein expression of MOGAT3 in RKO, RKO EC-R, HT29, and HT29 EC-R cells. Representative blots are shown. MOGAT3^KO^ RKO EC-R and HT29 EC-R, along with RKO EC-R-CTRL and HT29 EC-R-CTRL cell lines, were exposed to 2 μM encorafenib/4 μM cetuximab for 96 hours. Bottom: Relative OD value was assessed to determine cell viability by the CCK-8 assay (*n* = 3). (**C**–**E**) Xenograft tumor size in nude mice inoculated with RKO EC-R cells (CTRL) or MOGAT3^KO^ RKO EC-R cells, and treated with just encorafenib-cetuximab or encorafenib-cetuximab in combination with i.p. injection of DAG. (**C**) Tumor weight, (**D**) tumor DAG level, and (**E**) tumor growth in nude mice (*n* = 6). (**F**–**H**) Xenograft tumor size in nude mice inoculated with RKO cells (Nc) or RKO Oe-MOGAT3 cells and treated with encorafenib-cetuximab. (**F**) Xenograft tumor weight, (**G**) DAG level in tumor tissues, and (**H**) tumor growth (*n* = 6). (**I**–**K**) Xenograft tumor size in nude mice inoculated with encorafenib-cetuximab–resistant *BRAF^V600E^*-mutant mCRC tumor tissues. PDXs were treated with vehicle (PBS), 20 mg/kg encorafenib/20 mg/kg cetuximab, or MOGAT3 inhibitor PF-06471553 (Pf; 50 mg/kg) alone or in combination with encorafenib-cetuximab. (**I**) Xenograft tumor weight, (**J**) DAG level in tumor tissues, and (**K**) growth in nude mice (*n* = 6). The data are presented as mean ± SEM of 3 independent experiments. NS, no significance. ***P < 0.001 by 2-way ANOVA with Tukey’s multiple-comparison test (**B**, **E**, **H**, and **K**), 1-way ANOVA with Tukey’s multiple-comparison test (**C**, **D**, **I**, and **J**), or 2-tailed, unpaired *t* test (**F** and **G**).

Next, we sought to elucidate the mechanism underlying intratumoral DAG accumulation in resistant *BRAF^V600E^*-mutant mCRC. DAG synthase *MOGAT3* (Supplemental Figure 3A), the only upregulated gene in both the metabolic and DAG O-acyltransferase pathways (Supplemental Figure 3E), was found to be dramatically upregulated in resistant PDX tumors and RKO EC-R cells (Supplemental Figure 3, B and D). Previous studies indicated that MOGATs catalyze the synthesis of DAG from 2-monoacylglycerol and fatty acyl-CoA in the intestine (18, 21). Next, we observed MOGAT3, but not MOGAT1 or MOGAT2, markedly elevated in resistant PDX tumors and RKO EC-R cells compared with respective sensitive tumor cells (Figure 3A and Supplemental Figure 3C). Moreover, the protein level of MOGAT3 in parental RKO and HT29 cells was assessed upon monotherapy treatment (encorafenib or cetuximab). The Western blotting result showed that the MOGAT3 protein expression level was not changed upon monotherapy treatment, implying the acquired resistance of *BRAF^V600E^*-mutant mCRC to dual therapy responsible for MOGAT3 dysregulation (Supplemental Figure 3F). Next, we treated sensitive cells with encorafenib and cetuximab and assessed MOGAT3 protein levels. At the beginning of dual therapy, MOGAT3 protein levels showed no change in sensitive cells (RKO and HT29 cell lines). Under continuous pressure of dual therapy, MOGAT3 levels were restored (Supplemental Figure 3G). These results suggested that long-term induction of sensitive cells might induce an increase in MOGAT3. We next examined whether MOGAT3-mediated DAG accumulation drives anti-BRAF/EGFR therapy resistance. Knocking out MOGAT3 (MOGAT3^KO^) in combination with encorafenib/cetuximab treatment significantly inhibited RKO EC-R and HT29 EC-R cell growth and lowered DAG accumulation (Figure 3B and Supplemental Figure 3H). Moreover, MOGAT3^KO^ in parental cells (RKO or HT29) showed no effect on cell proliferation (Supplemental Figure 3I). Similar to the in vitro results, MOGAT3^^KO^^ in RKO EC-R cells restored the efficacy of encorafenib/cetuximab treatment in cell line–derived xenograft (CDX) tumors in vivo and reduced intratumoral DAG levels compared with the dual-therapy group (Figure 3, C–E). Treatment with DAG alone did not affect tumor growth (Figure 3, C and E). In contrast, restoring DAG levels reversed the increased sensitivity to encorafenib/cetuximab treatment caused by MOGAT3^KO^ in resistant tumors, leading to renewed CDX tumor growth (Figure 3, C–E). This suggests that the response to the dual therapy is contingent upon intratumoral DAG levels. Moreover, histological analysis of RKO EC-R CDX tumors revealed a statistically significant decrease in Ki67 expression and an increase in TUNEL-positive cells in the MOGAT3^KO^ group treated with encorafenib/cetuximab compared with the dual-therapy group, effects that were negated by DAG treatment (Supplemental Figure 3, J and K). Furthermore, we assessed whether overexpressed MOGAT3 in sensitive RKO cells (Oe-MOGAT3 RKO) would confer resistance to anti-BRAF/EGFR therapy in these cells. The tumor volume in the Oe-MOGAT3 RKO CDX group increased approximately 4-fold compared with the negative control (NC) RKO CDX group when treated with encorafenib/cetuximab in vivo (Figure 3, F and H). Intratumoral DAG levels were statistically higher in Oe-MOGAT3 RKO CDX tumors, aligning with the pattern observed in resistant tumors (Figure 3G). Histological assessment revealed a statistically significant rise in Ki67 and a reduction in TUNEL in Oe-MOGAT3 RKO CDX tumors, indicating that MOGAT3-mediated DAG accumulation may confer resistance to *BRAF^V600E^*-mutant mCRC (Supplemental Figure 3L). Next, we evaluated the impact of the MOGAT3 inhibitor PF-06471553 (Pf) on enhancing the efficacy of encorafenib/cetuximab in PDX tumors with acquired resistance. The triple combination led to a roughly 1-fold decrease in tumor volume and an approximately 2.5-fold reduction in intratumoral DAG levels in resistant PDX tumors relative to the dual-therapy control group (Figure 3, I–K). Monotherapy with Pf reduced the intratumoral DAG, but did not affect resistant PDX tumor growth, suggesting that MOGAT3-regulated levels of intratumoral DAG determine the treatment response to dual therapy (Figure 3, I–K). Histological assessment of resistant PDX tumors demonstrated statistically significant decreased levels of Ki67 and increased TUNEL in the triple regimen group compared with the dual or monotherapy treatment group (Supplemental Figure 3M). In addition, MOGAT3 inhibitor Pf combined with encorafenib/cetuximab reduced DAG and had an equivalent effect on growth inhibition in RKO EC-R and HT-29 EC-R cells (Supplemental Figure 3, N and O). Furthermore, triple therapy (BRAF + EGFR + MOGAT3 inhibitor) markedly increased apoptotic rates and the expression of proapoptotic proteins BAX and cleaved caspase-3/9, while it decreased the expression of the antiapoptotic protein Bcl2, compared with dual therapy (Supplemental Figure 3, P and Q). Owing to MOGAT3 being a pseudogene in mouse models, toxicity experiments were performed in rat models. Pf showed negligible toxicity in the rat blood index and histopathology (including heart, liver, kidney, and lung) (Supplemental Figure 3, R and S). These results demonstrated that targeting MOGAT3 overcomes the resistance of *BRAF^V600E^*-mutant mCRC to anti-BRAF/EGFR therapy by reducing intratumoral DAG.

### MOGAT3 inhibition disrupts DAG synthesis and boosts lipid oxidative phosphorylation, lowering intratumoral DAG.

**Figure 4:**
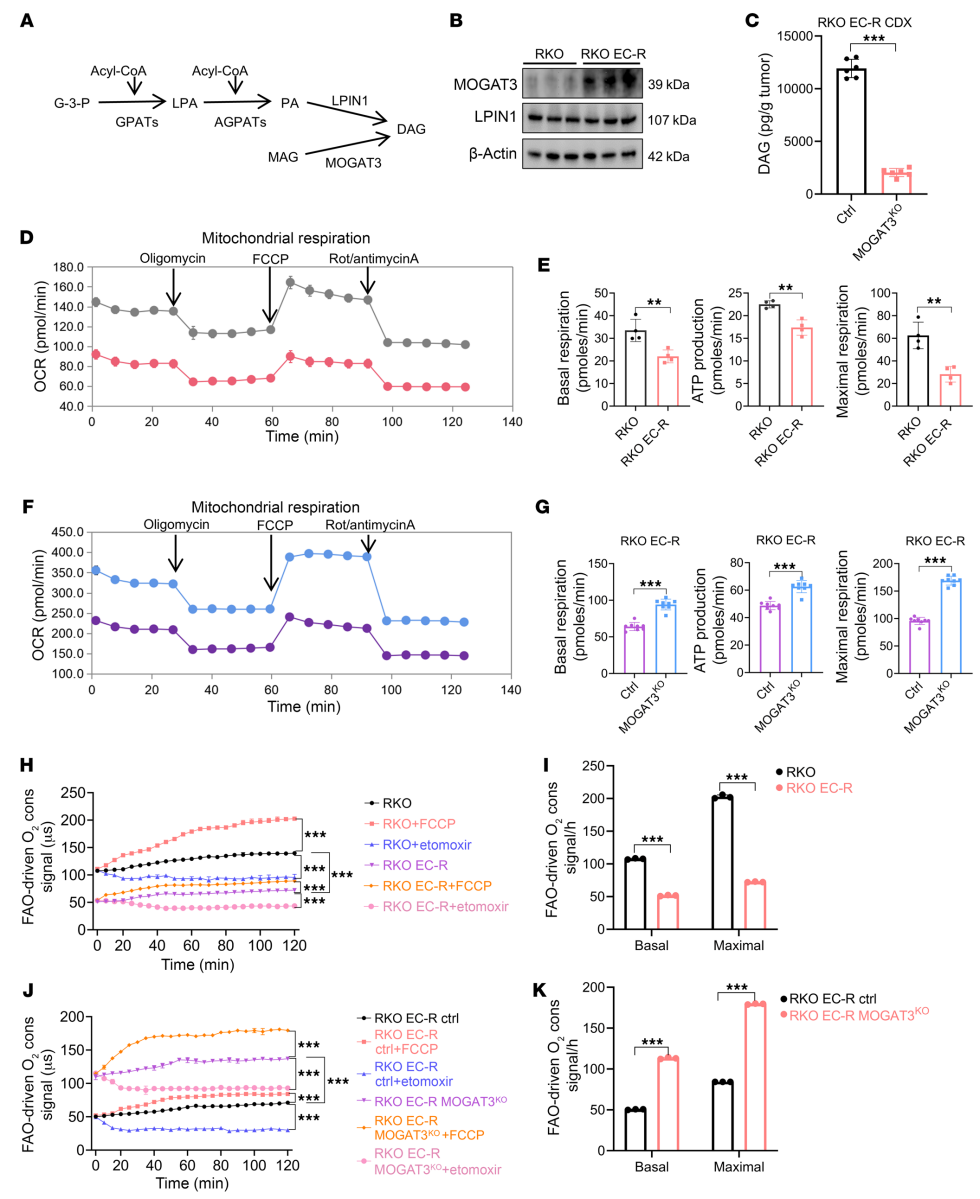
Highly expressed MOGAT3 promotes lipid synthesis and inhibits lipid OXPHOS, resulting in DAG accumulation. (**A**) Schematic diagram of the main DAG synthesis pathway. (**B**) Western blot showing the protein expression levels of LPIN1 and MOGAT3 in RKO and RKO EC-R cells. A representative blots is shown. (**C**) DAG level in RKO EC-R MOGAT3^KO^ CDX (*n* = 6). (**D**) Oxygen consumption rate (OCR) in RKO and RKO EC-R cells. Oligo, oligomycin; FCCP, carbonyl cyanide 4-trifluoromethoxy-phenylhydrazone; Rot, rotenone. (**E**) OXPHOS-related indicators were quantified (*n* = 4). (**F**) OCR in RKO EC-R and RKO EC-R MOGAT3^KO^ cells. (**G**) OXPHOS-related indicators were quantified (*n* = 8). (**H**–**K**) FAO assay of RKO and RKO EC-R cells (**H**) and RKO EC-R MOGAT3^KO^ cells (**J**). Cells treated with FCCP were used as the positive control, and cells treated with etomoxir (Eto) were used as the negative control. (**I** and **K**) Graphs show relative FAO rates (*n* = 3). The data are presented as mean ± SEM of 3 independent experiments. NS, no significance. **P < 0.01; ***P < 0.001 by 2-tailed, unpaired *t* test (**C**, **E**, **G**, **I**, and **K**) or 2-way ANOVA with Tukey’s multiple-comparison test (**H** and **J**).

Next, we examined the functions of MOGAT3 in regulating DAG synthesis in resistant *BRAF^V600E^*-mutant CRC cells. It has been reported that DAG synthesis relied on 2 pathways: the *sn*-glycerol-3-phosphate (G-3-P) pathway and the MOGAT-dependent pathway (22) (Figure 4A). We observed that LPIN1, the key to DAG synthesis in the G3P pathway, was unchanged in RKO and RKO EC-R cells, suggesting DAG synthesis is predominantly MOGAT3 dependent (Figure 4B). Moreover, the level of intratumoral DAG decreased in the MOGAT3^KO^ CDX group (Figure 4C), and the lipidomic analysis showed that MOGAT3 inhibition significantly reduced DAG-related lipid profiles in RKO EC-R cells (Supplemental Figure 4B). Furthermore, we assessed the live-cell oxygen consumption rate (OCR) to profile the respiration of RKO and RKO EC-R cells and to ascertain whether mitochondrial respiration is influenced by MOGAT3-mediated DAG accumulation. The basal, maximal, and ATP-linked OCR analyses indicated a significantly reduced OCR in RKO EC-R cells compared with RKO cells, suggesting inhibited oxidative phosphorylation (OXPHOS) in drug-resistant cells (Figure 4, D and E).

On the other hand, knockout of MOGAT3 did not affect LPIN1 protein levels in RKO EC-R cells, suggesting MOGAT3 regulates DAG synthesis in RKO EC-R cells (Supplemental Figure 4A). Additionally, basal, maximal, and ATP-linked OCR increased in MOGAT3^^KO^^ RKO EC-R cells relative to the control group (Figure 4, F and G). We examined fatty acid oxidation (FAO) in RKO EC-R cells to determine whether MOGAT3-regulated OCR stems from FAO. O__2__ consumption decreased with etomoxir treatment in both RKO EC-R and RKO cells, with RKO EC-R cells showing lower O__2__ consumption than RKO cells upon etomoxir treatment (Figure 4H). Moreover, there was a decrease in both basal and maximal respiration in RKO EC-R cells compared with RKO cells, indicating a substantial reduction in FAO in RKO EC-R cells (Figure 4, H and I). On the other hand, MOGAT3^^KO^^ notably increased FAO in RKO EC-R cells relative to control cells (Figure 4, J and K). These data suggest that MOGAT3 mediated DAG accumulation by promoting DAG synthesis and inhibiting FAO in *BRAF^V600E^*-mutant CRC cells.

### MOGAT3-induced DAG accumulation triggers MAPK rebound.

**Figure 5:**
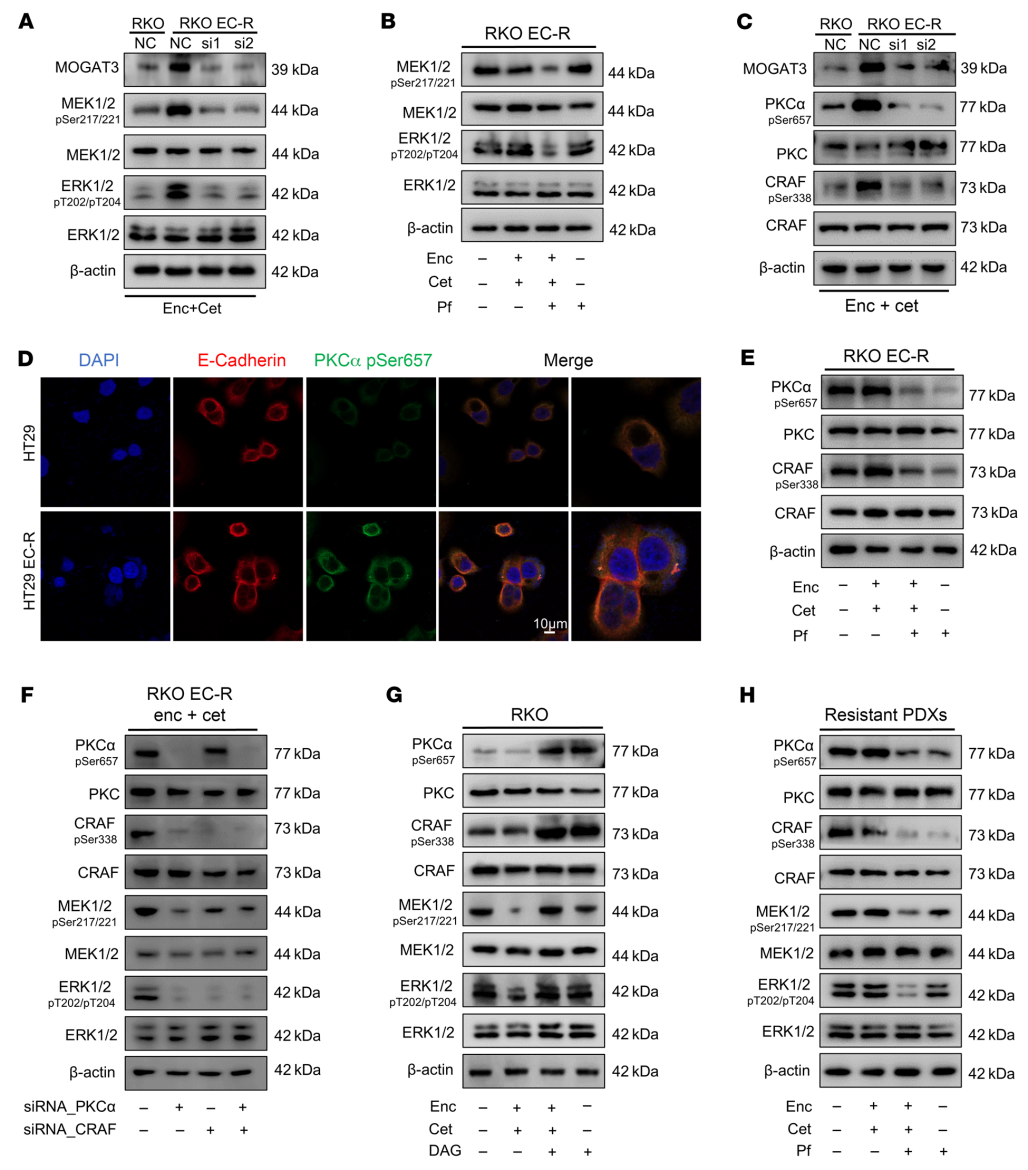
MOGAT3 reactivates MAPK through DAG-mediated PKCα/CRAF axis. (**A**) RKO EC-R cells transfected with siRNA-NC, siRNA-MOGAT3-1, or siRNA-MOGAT3-2 were treated with 2 μM encorafenib/4 μM cetuximab for 72 hours. Western blot assessing MOGAT3 and MEK/ERK signaling. Representative blots are shown. (**B**) Immunoblot analysis of MEK/ERK signaling in RKO EC-R cells treated with 2 μM encorafenib/4 μM cetuximab, 10 μM PF-06471553 (Pf), alone or in combination for 48 hours. (**C**) RKO EC-R cells transfected with siRNA-NC, siRNA-MOGAT3-1, or siRNA-MOGAT3-2 treated with 2 μM encorafenib/4 μM cetuximab for 72 hours. Western blot detecting MOGAT3 and PKCα/CRAF signaling. (**D**) Immunofluorescence of p-PKCα signaling in HT29 and HT29 EC-R cells. Representative images are shown. Scale bar: 10 μm. The images on the far right were further magnified ×4. (**E**) Immunoblot analysis of PKCα/CRAF signaling in RKO EC-R cells treated with 2 μM encorafenib/4 μM cetuximab, Pf (10 μM), alone or a combination of both for 48 hours. (**F**) Western blot detecting PKCα/CRAF and MEK/ERK signaling in RKO EC-R cells treated with siRNA-PKCα, siRNA-CRAF, or a combination of both for 48 hours. (**G**) Immunoblot analysis of PKCα/CRAF and MEK/ERK signaling in RKO cells treated with 0.25 μM encorafenib/0.5 μM cetuximab, 10 μM DAG, or a combination of both for 48 hours. (**H**) Western bolts detecting the intracellular signal change in encorafenib/cetuximab-resistant PDXs from Figure 3I. The tumor tissues were harvested for Western blotting to detect the indicated signaling proteins. A representative blot is shown from 3 independent experiments.

MAPK signaling rebound is recognized as an essential resistance mechanism in *BRAF*-mutant tumor treatment (23), so we tested whether a MOGAT3 inhibitor combined with dual therapy would inhibit p-ERK rebound more profoundly than anti-BRAF/EGFR treatment. Following dual therapy, the BRAF and EGFR statuses were first assessed in resistant cells. Western blot analysis indicated that BRAF and EGFR signaling was suppressed in RKO EC-R and HT29 EC-R cells, and resistant PDX tumors after treatment (Supplemental Figure 5E). Increased ERK and MEK phosphorylation was noted in RKO EC-R cells (Figure 5A), while MOGAT3 levels rose in resistant cells, predominantly localizing to the perinuclear region of the cytoplasm (Supplemental Figure 5, B and D). Genetic or pharmacological inhibition of MOGAT3, combined with anti-BRAF/EGFR therapy, markedly suppressed the upsurge in ERK and MEK phosphorylation (Figure 5, A and B). MOGAT3 knockdown significantly reduced DAG levels in RKO EC-R cells (Supplemental Figure 5A). We then investigated whether DAG accumulation, mediated by MOGAT3, leads to the reactivation of the MEK/ERK pathway. A critical role of DAG in signal transduction is its regulation of various cellular processes via the activation of PKC, which occurs when DAG binds to the C1 domains of PKC, prompting its phosphorylation (24). As expected, we observed increased phosphorylated PKCα (p-PKCα) in RKO EC-R cells (Figure 5C). To determine where DAG accumulates, we utilized the response of PKCα to DAG. We found that DAG levels were elevated in resistant cells and activated p-PKCα was localized to the cell membrane, colocalizing with E-cadherin (Figure 5D and Supplemental Figure 5, C and F). Previous studies reported that CRAF activation is a compensatory mechanism for BRAF inhibition (25), and CRAF can be phosphorylated by PKCα (26). Consistently, we observed that p-CRAF was elevated in RKO EC-R cells compared with RKO cells. Combined genetic or pharmacological inhibition of MOGAT3 with anti-BRAF/EGFR treatment effectively suppressed PKCα/CRAF signaling activation in RKO EC-R cells (Figure 5, C and E). To test whether PKCα activation led to a MAPK rebound via CRAF activation, we knocked down PKCα and CRAF in RKO EC-R cells. The results indicated that PKCα knockdown diminished CRAF-mediated phosphorylation of ERK and MEK in RKO EC-R cells under dual-therapy treatment (Figure 5F). And CRAF knockdown suppressed MEK/ERK signaling without affecting PKCα levels (Figure 5F). The activation of PKC through DAG or the PKC agonist phorbol 12-myristate 13-acetate (PMA) in RKO cells led to CRAF phosphorylation under dual-therapy treatment, triggering the MEK/ERK signaling cascade (Supplemental Figure 5G). This activation was eliminated by the PKC inhibitor PKC-IN-1 (Supplemental Figure 5G). Similarly, DAG plus dual therapy activated p-PKCα/p-CRAF/p-MEK/p-ERK signaling in RKO cells (Supplemental Figure 5G). DAG-only treatment–activated p-PKCα/p-CRAF showed no exacerbating effect on p-MEK/p-ERK signaling compared to the control group (Figure 5G). On the contrary, the triple therapy inhibited MOGAT3-mediated DAG accumulation and interrupted DAG/PKCα/CRAF signaling in resistant PDX tumors (Figure 5H). Treatment with Pf alone could inhibit p-PKCα/p-CRAF signaling, but had no impact on p-MEK/p-ERK signaling, elucidating why Pf monotherapy is ineffective at halting the growth of drug-resistant tumors (Figure 5H and Figure 3I). In addition, overexpression of MOGAT3 in RKO cells caused an increase in DAG levels, which in turn promoted PKCα/CRAF signaling activation in CDX tumors, resulting in resistance to encorafenib/cetuximab treatment (Supplemental Figure 5H). These findings indicate that the accumulation of DAG mediated by MOGAT3 leads to PKCα/CRAF activation, thereby linking to the activation of MEK/ERK signaling.

### Hypoxia-induced resistance upregulates MOGAT3, enhancing DAG accumulation and tumor resilience.

**Figure 6:**
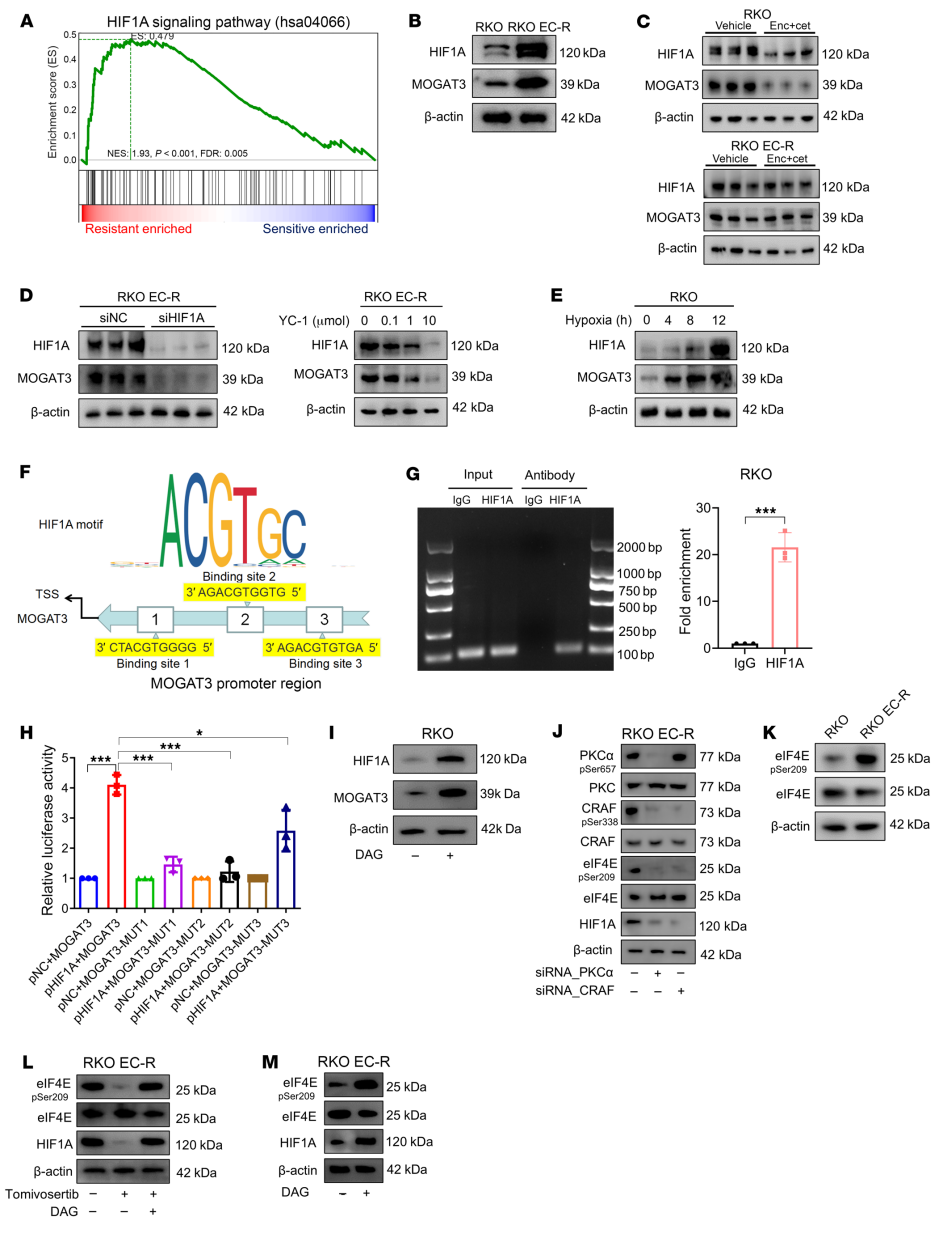
Accumulated DAG enhances *MOGAT3* transcription expression through PKCα/CRAF/eIF4E/HIF1A signaling activation. (**A**) Gene set enrichment analysis (GSEA) of resistant tumors versus sensitive tumors (*n* = 3) shows enhanced HIF1A signaling pathway. Normalized enrichment score (NES) and nominal *P* value were provided according to GSEA. (**B**) Immunoblot analysis of MOGAT3 and HIF1A in RKO and RKO EC-R cells. (**C**) Immunoblot analysis of HIF1A and MOGAT3 in RKO and RKO EC-R cells treated with encorafenib-cetuximab for 48 hours. (**D**) Immunoblot analysis of HIF1A and MOGAT3 in RKO EC-R cells after siRNA-HIF1A knockdown for 72 hours (left) or treated with the indicated concentrations of YC-1 (1 μM) for 24 hours (right). (**E**) Immunoblot analysis of HIF1A and MOGAT3 in RKO cells after hypoxia for 0, 4, 8, and 12 hours. (**F**) Illustration of HIF1A site and the predicted HIF1A site in the *MOGAT3* promoter. The HIF1A motif in the ACGTGC promoter was predicted by JASPAR 2022 (https://jaspar2022.genereg.net/). (**G**) Left: ChIP-PCR confirms that HIF1A can directly transcriptionally regulate *MOGAT3*. Right: RT-qPCR of ChIP-PCR (*n* = 3). (**H**) Luciferase reporter assay shows that HIF1A overexpression significantly activated the promoter activity of MOGAT3 (*n* = 3). (**I**) Immunoblot analysis of MOGAT3 and HIF1A in RKO cells treated with DAG for 48 hours. (**J**) Immunoblot analysis of p-CRAF/CRAF, p-PKCα/PKC, p-eIF4E/eIF4E, and HIF1A in RKO EC-R cells treated with siRNA-PKCα or siRNA-CRAF for 48 hours. (**K**) Immunoblot analysis of p-eIF4E and eIF4E in RKO and RKO EC-R cells. (**L**) Immunoblot analysis of p-eIF4E/eIF4E and HIF1A in RKO EC-R cells after treatment with p-eIF4E inhibitor (10 μM) or plus DAG (10 μM) for 24 hours. (**M**) Immunoblot analysis of p-eIF4E/eIF4E and HIF1A in RKO EC-R cells treated with DAG for 48 hours. The data are presented as mean ± SEM of 3 independent experiments. NS, no significance. *P < 0.05; ***P < 0.001 by 2-tailed, unpaired *t* test (**G**) or 1-way ANOVA with Tukey’s multiple-comparison test (**H**).

Hypoxia and nutrient shortages in tumor mass are accompanied by long-term treatment (27–29). Our GSEA results indicated that the HIF1A pathway was significantly upregulated in resistant PDX tumors, and we observed that HIF1A protein expression was increased in resistant cells compared with parental cells (Figure 6, A and B, and Supplemental Figure 6, A and B). To assess whether drug resistance status contributes to HIF1A elevation, we measured the HIF1A protein expression in RKO and RKO EC-R cells upon encorafenib/cetuximab treatment. Surprisingly, encorafenib/cetuximab treatment inhibited the protein level of HIF1A in the sensitive RKO cells, but not in the resistant RKO EC-R cells, implying the inability to downregulate HIF1A was associated with drug resistance status (Figure 6C). Next, we examined whether HIF1A, a well-known transcription factor (30), regulated *MOGAT3* transcriptional expression. Inhibiting HIF1A by either siRNA or pharmacological inhibitor YC1 reduced the MOGAT3 protein expression level in the RKO EC-R cells (Figure 6D). Moreover, forced expression of HIF1A through hypoxic induction increased the protein expression level of MOGAT3 in the RKO cells (Figure 6E). The JASPAR-predicted binding motif suggested HIF1A bound to the MOGAT3 promoter region, and the ChIP-PCR result revealed direct binding of HIF1A to the MOGAT3 promoter (Figure 6, F and G). Moreover, site-directed mutagenesis combined with luciferase assay indicated that binding sites 1 and 2 in the MOGAT3 promoter mainly mediated HIF1A-induced promoter activity (Figure 6H). We next asked whether HIF1A was regulated by DAG-mediated PKCα/CRAF signaling. In addition, we found that DAG-only treatment increases HIF1A protein expression in RKO cells and sensitive PDX tumors (Figure 6I and Supplemental Figure 6, C and D). Surprisingly, knocking down PKCα or CRAF suppressed HIF1A protein expression in RKO EC-R cells (Figure 6J). Previous studies reported that eIF4E, a rate-limiting component of eukaryotic translation, could increase the translation of HIF1A protein (31, 32), and we observed that p-eIF4E was elevated in RKO EC-R cells compared with RKO cells (Figure 6K). On the other hand, an inhibited PKCα/CRAF cascade suppressed eIF4E phosphorylation (Figure 6J) and directly inhibiting p-eIF4E with tomivosertib reduced HIF1A protein expression in RKO EC-R cells (Figure 6L), which was reversed by DAG supplementation, indicating PKCα/CRAF signaling promotes HIF1A elevation through eIF4E phosphorylation. HIF1A and eIF4E phosphorylation levels were increased in RKO EC-R cells under solo DAG treatment compared with the control group (Figure 6M). Then, to further investigate the causes behind the accumulation of MOGAT3 protein, we studied the impact of protein synthesis and degradation on MOGAT3 levels. We treated both sensitive and resistant cells with cycloheximide (CHX), a protein synthesis inhibitor, at various time points. Western blot analysis revealed that the rate of MOGAT3 protein degradation was similar in both sensitive and resistant cells (Supplemental Figure 6, E–H). Additionally, we treated sensitive and resistant cells with MG132, a proteasome inhibitor, and Eeyarestatin I (Eer I), an endoplasmic reticulum–associated degradation (ERAD) inhibitor. The results indicated that MOGAT3 protein levels rose following Eer I treatment, while MG132 treatment did not alter MOGAT3 levels in either cell type (Supplemental Figure 6, I and J). The increase in MOGAT3 protein levels following Eer I treatment was consistent in resistant and sensitive cell groups, suggesting that ERAD does not influence MOGAT3 accumulation in cells that are resistant to therapy (Supplemental Figure 6, I and J). These findings indicate that resistance-induced hypoxia promotes MOGAT3 transcriptional activation, and MOGAT3-mediated DAG accumulation reinforces resistance status through the PKCα/CRAF/eIF4E/HIF1A cascade.

### Fenofibrate overcomes the acquired resistance of BRAF^^V600E^^-mutant mCRC to anti-BRAF/EGFR therapy.

**Figure 7:**
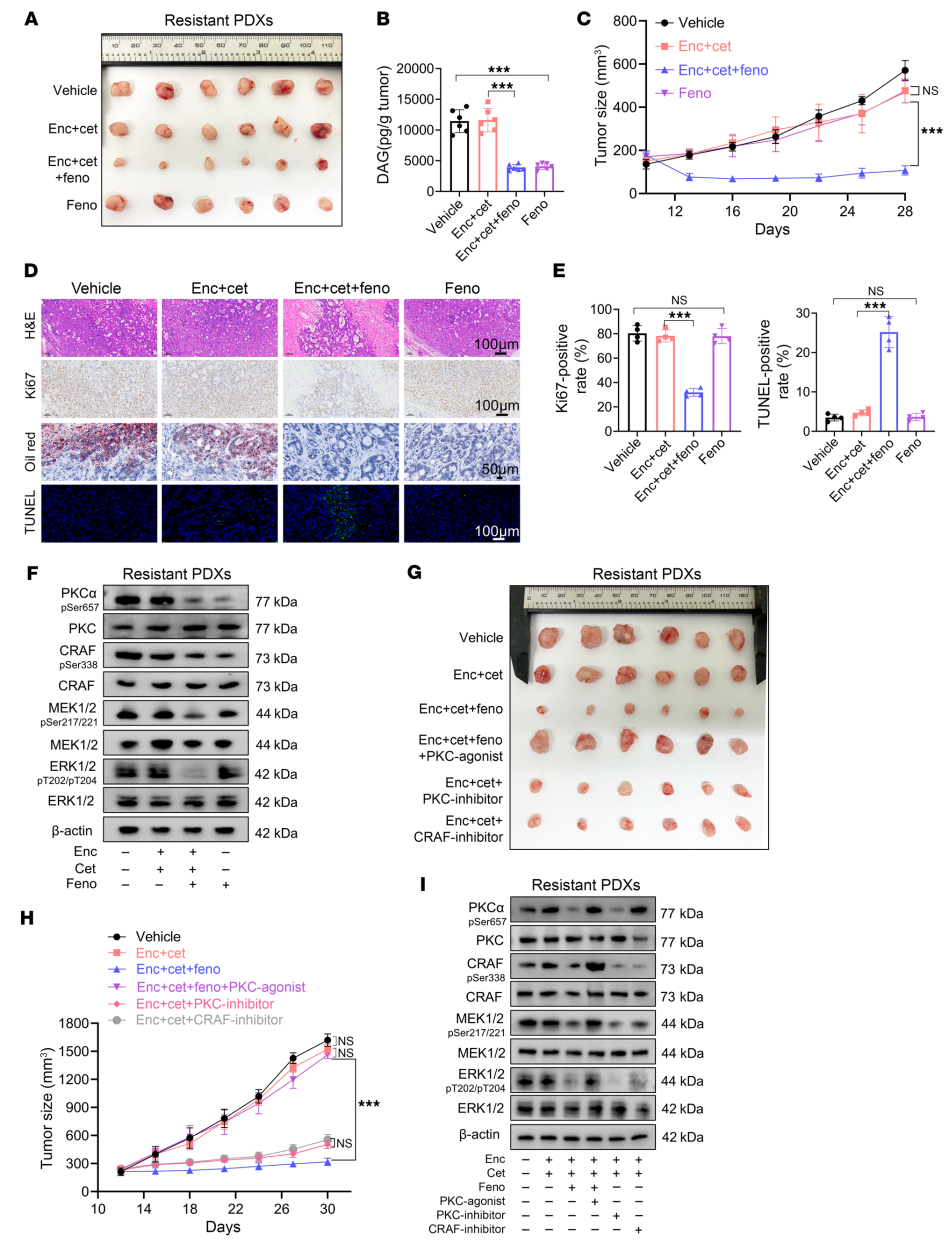
Reducing intratumoral DAG with fenofibrate overcomes the resistance of *BRAF^V600E^*-mutant mCRC tumors upon dual therapy. (**A**–**C**) Xenograft tumor size in nude mice inoculated with encorafenib- and cetuximab-resistant *BRAF^V600E^*-mutant mCRC tumor tissues treated with vehicle (PBS), encorafenib (20 mg/kg), cetuximab (20 mg/kg), or fenofibrate (100 mg/kg), alone or all 3 together (*n* = 6). (**A**) Representative tumor images. (**B**) Quantification of DAG levels in tumor tissues. (**C**) Quantification of tumor growth (*n* = 6). (**D**) Representative images of H&E, Ki67, Oil Red O, and TUNEL staining. (**E**) The quantitation of Ki67 and TUNEL (*n* = 4). (**F**) Immunoblot analysis of PKCα/CRAF and MEK/ERK signaling in tumor tissues related to **A**. (**G** and **H**) Xenograft tumor size in nude mice inoculated with encorafenib- and cetuximab-resistant *BRAF^V600E^*-mutant mCRC tumor tissues orally treated with vehicle (PBS), encorafenib/cetuximab (20 mg/kg, 20 mg/kg), encorafenib/cetuximab/fenofibrate (20 mg/kg, 20 mg/kg, 100 mg/kg), encorafenib/cetuximab/fenofibrate/PMA (20 mg/kg, 20 mg/kg, 100 mg/kg, 20 mg/kg), encorafenib/cetuximab/PKC-IN-1 (20 mg/kg, 20 mg/kg, 30 mg/kg), or encorafenib/cetuximab/RAF-IN-1 (20 mg/kg, 20 mg/kg, 30 mg/kg) (*n* = 6). (**G**) Representative images of xenograft tumor growth in nude mice. (**H**) Quantification of tumor growth. (**I**) Western bolt assessing the protein expression of PKCα/CRAF/MEK/ERK signaling in encorafenib/cetuximab-resistant PDXs from **G**. The tumor tissues were harvested for Western blotting to detect the indicated signaling proteins. A representative blot is shown from 3 independent experiments. The data are presented as mean ± SEM of 3 independent experiments. NS, no significance. ***P < 0.001 by 1-way ANOVA with Tukey’s multiple-comparison test (**B** and **E**) or 2-way ANOVA with Tukey’s multiple-comparison test (**C** and **H**).

Acknowledging that the addition of a MOGAT3 inhibitor to anti-BRAF/EGFR therapy for the treatment of *BRAF^V600E^*-mutant mCRC is unlikely to be clinically acceptable, currently owing to concerns about toxicity in patients, we explored whether targeting DAG could lead to a more clinically appropriate regimen. Fenofibrate, an FDA-approved clinical drug, was designed to treat patients with hypertriglyceridemia, primary hypercholesterolemia, or mixed dyslipidemia (33) and can effectively reduce the levels of DAG. To evaluate the effectiveness of fenofibrate in resistant tumors, we treated resistant PDX tumors with vehicle (PBS), fenofibrate, encorafenib/cetuximab, or fenofibrate plus encorafenib/cetuximab in vivo (Figure 7A and Supplemental Figure 7A). Triple therapy (fenofibrate combined with encorafenib/cetuximab) significantly inhibited resistant PDX tumor growth, and dual therapy or fenofibrate monotherapy showed modest inhibition compared with the vehicle group (Figure 7, A and C). As expected, the levels of DAG in resistant PDX tumors were dramatically decreased upon fenofibrate treatment (Figure 7B). In addition, histological analysis revealed that the triple therapy markedly enhanced TUNEL staining and decreased Ki67 expression in resistant PDX tumors (Figure 7, D and E). Then, we explored whether the efficacy of triple therapy is dependent on MAPK signaling reduction. To this end, PKCα/CRAF/MEK/ERK signaling was assessed in resistant PDX tumors. Western blotting showed that the triple treatment inhibited DAG/PKCα/CRAF signaling (Figure 7F). Consistent with the Pf-only treatment effects on resistant PDX tumors, fenofibrate-only treatment inhibited DAG/PKCα/CRAF signaling, but showed no impact on MEK/ERK signaling (Figure 7F). Of note, the PKC agonist PMA blocked the inhibition of tumor growth upon triple therapy, and PKCα or CRAF inhibitors (RAF-IN-1, PKC-IN-1) combined with encorafenib/cetuximab treatment suppressed resistant PDX tumor growth (Figure 7, G and H and Supplemental Figure 7C). Western blotting results showed PKCα agonists reconnected the PKCα/CRAF signaling and inhibited treatment outcome in triple therapy (Figure 7I). Elevating DAG enhanced HIF1A, implying that reducing DAG may modulate MOGAT3 (Figure 6I and Supplemental Figure 6C). To test whether fenofibrate influences MOGAT3, we measured its expression in resistant cells after fenofibrate treatment. Indeed, fenofibrate reduced MOGAT3 protein levels in RKO EC-R, HT29 EC-R, and resistant PDX tumors (Supplemental Figure 7B). Together, our data provide compelling evidence that fenofibrate overcomes the resistance of *BRAF^V600E^*-mutant mCRC tumors to encorafenib/cetuximab treatment, depending on the MAPK signaling inhibition.

## Discussion

Despite the benefits of the recently approved encorafenib/cetuximab combination therapy for *BRAF^V600E^*-mutant mCRC patients’ survival, the duration time of this dual therapy is far from satisfactory. Improving the durability of treatment effects of anti-BRAF/EGFR therapy in resistant *BRAF^V600E^*-mutant mCRC patients is urgently needed. Ana Ruiz-Saenz et al. recently reported that targeted inhibition of BRAF with and without EGFR inhibition in *BRAF^V600E^*-mutant mCRC systematically activated SRC in parallel with MAPK signaling (7). *RNF43* mutations were found in *BRAF^V600E^*-mutant mCRC patients who were partially resistant to anti-BRAF treatment with and without anti-EGFR, and correlated with combination therapy efficiency (8). We found that SRC inhibition alone or combined with anti-BRAF treatment with or without anti-EGFR did not affect the tumor growth in our resistant models (data not shown). Moreover, whole-exome sequencing analysis revealed no consistent mutations in resistant PDX tumors such as those in *RNF43*, ruling out genomic mutation as a cause of resistance. Our results provide an insight into how intratumoral DAG levels affect the response to anti-BRAF/EGFR therapy in *BRAF^V600E^*-mutant mCRC by activating PKCα/CRAF/MEK/ERK signaling, leading to acquired resistance. We showed that MOGAT3-mediated DAG accumulation triggers a rebound in the MAPK pathway, conferring resistance to encorafenib/cetuximab therapy. Noticeably, resistance-induced hypoxia leads to increased *MOGAT3* transcription, with MOGAT3-mediated DAG accumulation strengthening resistance via elevated PKCα/CRAF/eIF4E/HIF1A signaling. In contrast, inhibiting MOGAT3 decreases intratumoral DAG and dampens PKCα/CRAF/MEK/ERK signaling, enhancing the effectiveness of encorafenib/cetuximab dual therapy in resistant *BRAF^V600E^*-mutant mCRC. Interestingly, fenofibrate, a clinically actionable drug, overcomes the acquired resistance to encorafenib/cetuximab therapy in *BRAF^V600E^*-mutant mCRC in vivo through DAG reduction and subsequent inhibition of PKCα/CRAF/MEK/ERK signaling. Our study uncovered a lipid-mediated resistance mechanism in *BRAF^V600E^*-mutant mCRC and suggested a viable clinical approach to counter resistance to anti-BRAF/EGFR therapy.

DAGs are central to multiple metabolic processes and mediate signaling transduction (34). Dysregulation of DAG metabolism is thought to affect cellular signaling adversely and is involved in developing various disease states, such as insulin resistance (35). Most notably, PKC senses DAG generated in different cellular compartments in various physiological processes (36). Recent studies reported that DAG kinase α (DGKα) facilitated phosphatidic acid synthesis by consuming DAG to negatively regulate the lipogenic transcription factor SREBP-1 in CRC tumor cells, implying the signal transduction function of DAG in controlling tumor growth (37). We report that the level of intratumoral DAG determines the response of anti-BRAF/EGFR therapy in *BRAF^V600E^*-mutant mCRC, enriching our understanding of DAG’s regulation of tumor-targeted therapy. DAG accumulation induced by resistance is mainly concentrated in tumor cells, and we demonstrated that DAG-mediated PKCα/CRAF activation results in combination therapy treatment failure. The increase or decrease in DAG in *BRAF^V600E^*-mutant tumors does not independently affect tumor growth; it is related to therapeutic interventions. Further research indicated that DAG modulates p-PKCα/p-CRAF signaling without impacting p-MEK/p-ERK pathways. The proliferation of resistant *BRAF^V600E^*-mutant tumors is governed by BRAF/CRAF-mediated MEK/ERK signaling. In *BRAF^V600E^*-mutant tumors with resistance, targeting either CRAF or BRAF alone does not disrupt MEK/ERK signaling, which is why neither MOGAT3 inhibition (with Pf) nor DAG reduction (through fenofibrate) is sufficient to hinder the growth of resistant tumors. On the other hand, MOGAT3-mediated DAG accumulation elevated the phosphorylation of eIF4E mediated by PKCα/CRAF activation and then translationally promoted HIF1A protein expression, reinforcing hypoxia and acquired resistance statuses (32, 38). Short-term dual therapy showed no effect on HIF1A. DAG plus dual therapy increased HIF1A protein expression, suggesting acquired resistance–induced hypoxia, and the resistant status in *BRAF^V600E^*-mutant mCRC is bilaterally enhanced under DAG accumulation. Moreover, our data showed that DAG enhances HIF1A signaling in *BRAF^V600E^*-mutant mCRC. HIF1A, a critical transcription factor for cancer cell survival, orchestrates the expression of genes related to metabolism and survival, enabling adaptation to adverse microenvironments (39). The involvement of HIF1A in glucose metabolism, particularly in the context of the Warburg effect, has been the subject of extensive research over the last 2 decades (40). Upon activation, HIF1A stimulates the uptake of fatty acids and enhances lipid storage (41). Furthermore, HIF1A inhibits FAO by downregulating PGC-1α, CPT1A, and acyl-CoA dehydrogenases, and it also hampers lipolysis by repressing ATGL (42). In line with these findings, we observed a decrease in FAO and CPT1A expression in resistant cells with elevated DAG levels compared with sensitive cells. This may account for the observed inhibition of FAO in resistant *BRAF^V600E^*-mutant CRC cells with high DAG levels. These results suggest an intimate association between the lipid metabolite accumulation in modulating the tumor resistance of mCRC with the *BRAF^V600E^* mutation, which provided a therapeutic insight into overcoming drug resistance via metabolic rewiring.

MOGAT3 is primarily expressed in the gastrointestinal tract (16). As an integral membrane enzyme, MOGAT3 catalyzes the acylation of monoacylglycerol (MAG) and DAG, promoting DAG synthesis (18). Previous evidence has suggested that MOGAT3 has MOGAT and DAG O-acetyltransferase activity (36), yet its role in, and impact on, disease progression remain unclear. We found that hypoxia induced by acquired resistance upregulates MOGAT3 transcription, leading to DAG accumulation and affecting the efficacy of the dual therapy. Moreover, upregulated MOGAT3 enhanced DAG synthesis while simultaneously reducing its breakdown, promoting DAG accumulation in a bidirectional manner.

Recent studies have illustrated the mechanism of treatment failure of anti-BRAF with and without anti-EGFR in *BRAF^V600E^*-mutant mCRC, but have not resolved the resistance issue in our models. The resistance of *BRAF^V600E^*-mutant tumors to anti-BRAF/EGFR therapies is primarily attributed to the rebound activation of MAPK signaling (23). Indeed, our results showed that MOGAT3-mediated DAG accumulation drives resistance through PKCα/CRAF-mediated MAPK reactivation. Clinical studies have demonstrated that the synergistic treatment of MEK inhibitors has no impact on prolonging the duration of patients’ anti-BRAF/EGFR therapies (6). Developing a clinical treatment to overcome drug resistance is time consuming and labor intensive. Our data demonstrated that MOGAT3/DAG signaling drives acquired resistance in *BRAF^V600E^*-mutant mCRC, and targeting DAG in an equivalent manner to MOGAT3 inhibition overcomes the resistance. Impressively, fenofibrate plus encorafenib/cetuximab ideally inhibits resistant tumor growth, with reductions in levels of intratumoral DAG. In our model, the levels of DAG in *BRAF^V600E^*-mutant mCRC tumors determined the efficiency of dual therapy. Lower DAG by MOGAT3 inhibition made the resistant cells responsive to dual therapy. On the other hand, FAO was inhibited in resistant cells compared with sensitive cells, which might contribute to high levels of DAG. Lower DAG by fenofibrate re-sensitized the resistant *BRAF^V600E^*-mutant mCRC tumors to dual therapy. These effects of fenofibrate indicate that DAG-mediated downstream activation was disrupted by fenofibrate. Furthermore, our results illustrated that DAG accumulation also increases the expression of MOGAT3 in a transcriptional manner to strengthen drug resistance. Lowering DAG with fenofibrate could reduce DAG levels and inhibit MOGAT3 expression. This triple therapy has shown clinical promise in overcoming resistance in *BRAF^V600E^*-mutant mCRC. Moreover, we noted that elevated blood lipids correlate with resistance to encorafenib/cetuximab combination therapy in PDX models. During the follow-up of clinical drug treatment, we observed an increase in serum lipids. This increase seems to be related to the ineffectiveness of the encorafenib/cetuximab combination therapy, and further investigation is warranted.

In conclusion, our results demonstrate that MOGAT3-mediated DAG accumulation has a dominant role in mediating the acquired resistance of *BRAF^V600E^*-mutant mCRC to anti-BRAF/EGFR therapy. We show that resistance-induced hypoxia promotes MOGAT3-mediated DAG accumulation and drives PKCα/CRAF/MEK activation; in parallel, accumulated DAG reinforces resistant status via PKCα/CRAF/eIF4E/HIF1A signaling activation. We propose a clinically viable enhancement strategy involving triple therapy with fenofibrate combined with encorafenib/cetuximab to improve treatment efficiency in *BRAF^V600E^*-mutant mCRC.

## Methods

### Sex as a biological variable.

Our study exclusively examined female mice. It is unknown whether the findings would be similar for male mice, although we would not expect significant differences in the results.

### Patient samples.

The established PDX (derived from the primary tumor) originated from a 68-year-old male patient who presented with primary transverse colon cancer with liver metastasis and underwent laparoscopic left colectomy. Molecular pathology testing found that the patient had wild-type *RAS*, *BRAF^V600E^*, *TP53* mutation, and microsatellite stability (MSS) status. Before surgery, the patient had not received anti-BRAF/EGFR therapy. The tumors in situ were directly snap-frozen or fixed in formalin and embedded in paraffin for further use.

### PDX.

Fresh *BRAF^V600E^*-mutant mCRC tissue was collected in RPMI 1640 medium with antibiotics, rinsed in PBS, and transplanted subcutaneously into the groins of 4-week-old female BALB/c nude mice. Sedation and analgesia were performed using ketamine, medetomidine, and buprenorphine. Upon reaching generation 3, tumor fragments were transplanted into nude mice. A tumor size of 150 mm^3^ was defined as the baseline as a control time point for the efficacy of subsequent dosing (43). Mice were randomly assigned to a cohort, and drugs or vehicles were blindly administered daily by oral gavage and i.p. injection twice a week. Encorafenib was administered orally at 20 mg/kg daily and cetuximab by i.p. injection at 20 mg/kg, twice weekly (19). Tumor size was measured by digital calipers every 3 days. After the treatment with BRAF/EGFR inhibitors, the subcutaneous tumors of mice continued to decrease in volume, defined as the BRAF/EGFR inhibitors’ sensitive time. An initial reduction in tumor size in the experimental group followed by a regrowth of more than 150 mm^3^ represented a successful establishment of a PDX model that is resistant to BRAF/EGFR inhibitors, which was defined as the BRAF/EGFR inhibitors’ resistant time. The sensitive and resistant tissues were reinoculated in nude mice, respectively, using the exact dosage as above, to validate PDX tumor response to BRAF/EGFR inhibitors. In the follow-up PDX experiments, we used sensitive or resistant tissues for PDX modeling. Mice were sacrificed at 28 days following the start of treatment or when tumors reached a volume of 1500 mm^3^. The investigators were blinded for the evaluation of the results. Once the PDXs were obtained, blood samples were collected from the eyelids of nude mice, after which mice were sacrificed to obtain tumor tissues.

### Cell lines and drug treatment.

Two CRC cell lines, HT29 and RKO cells with a *BRAF^V600E^* mutation, were obtained from the American Type Culture Collection (ATCC). The human embryonic kidney cell line HEK-293T was purchased from the Cell Bank of the Shanghai Academy of Chinese Sciences. The mutational status of these cell lines utilized in this research can be accessed from the Cancer Cell Line Encyclopedia (CCLE) database and a prior study (44). All cell lines were cultured in Dulbecco’s modified essential medium (DMEM) or McCoy’s 5A medium containing 100 μg/mL streptomycin, 100 μg/mL penicillin, and 10% fetal bovine serum (FBS; Gibco). The cells were incubated at 37°C in a 5% humidified CO__2__ atmosphere. All the cell lines utilized in the study were negative for mycoplasma contamination (Lonza, LT07-318). DAG (Sigma-Aldrich, 24529-88-2) was dissolved in fresh dimethyl sulfoxide (DMSO) for a stock solution at 50 mM (or 50 mg/mL for the in vivo study). Similarly, TAG (Sigma-Aldrich, 1716-07-0) was dissolved in fresh DMSO to 50 mM. A working solution was added with pre-set DAG and TAG concentrations by mixing common serum-free medium proportionately. Encorafenib (MCE, HY-15605), cetuximab (MCE, HY-P9905), and PF-06471553 (MCE, HY-108339) were used to treat the cells after diluting according to the manufacturer’s instructions.

### CDX.

Approximately 2 × 10^6^ RKO EC-R or RKO EC-R-MOGAT3^KO^ cells were subcutaneously injected into the right hind limbs of BALB/c nude mice. Treatment began 1 week following the injection. The mice were randomized into 3 groups (*n* = 6 per group) and i.p. injected with vehicle (PBS), cetuximab (20 mg/kg/i.p. twice per week) plus encorafenib oral administration (20 mg/kg/day) together or combined with DAG i.p. injected (50 mg/kg/day). Tumor growth was recorded every 3 days from 1 week after inoculation by measurement of 2 perpendicular diameters using the formula 4π/3 × (width/2)^2^ × (length/2). Mice were sacrificed 4 weeks after inoculation. The masses of tumors (mg) derived from treatments were compared. In the MOGAT3 overexpression model, 2 × 10^6^ cells (RKO NC, RKO Oe-MOGAT3) were in a mixture of PBS in a volume of 100 μL, which was then injected into the subserous layer of the middle of nude mice cecum. After 4 weeks, all mice were sacrificed.

### Biochemical indicator quantification.

The levels of diglycerides in PDX tumor lysates were measured using the Diacylglycerol Assay Kit (Cloud-Clone Corp, CEC038Ge) following the manufacturer’s instructions. AST, ALT, CR, and BUN levels in rat serum were measured accordingly using the Purebio Assay Kit (ALT01, AST01, URE01, G034) following the manufacturer’s instructions and were detected in Automatic Biochemical Analyzer LWC400 (Landwind).

### Cell viability assay.

Cell viability was assessed using the Cell Counting Kit-8 (CCK8) from Dojindo Molecular Technologies, according to the manufacturer’s instructions. The absorbance was measured at 450 nm using a microplate reader. The experiments were conducted in triplicate.

### DAG and TAG assay.

Intracellular and tissue DAG levels were determined with a DAG ELISA Kit (Cloud Clone Corp, CEC038Ge), and TAG was detected using a TAG Content Enzymatic Assay kit (Applygen, E1013-50) according to the manufacturer’s instructions.

### RNA-seq analysis and whole-exome sequencing.

Total RNA of indicated tumor tissues from baseline, sensitive, and resistant periods were extracted using TRIzol reagent (Invitrogen) according to the manufacturer’s protocol. RNA purity, quantification, and integrity were evaluated. Then, the libraries were constructed using VAHTS Universal V6 RNA-seq Library Prep Kit according to the manufacturer’s instructions. Subsequently, paired-end sequencing on an Illumina NovaSeq 6000 (Lianchuan Biotech Co., Ltd) was performed following the vendor’s recommended protocol. The total DNA was extracted using QIAamp DNA FFPE Tissue (QIAGEN). Then, the DNA, which was fragmented using the M220 focused ultrasonicator (Covaris), was subjected to sequencing library construction. Exome capture was performed using SureSelect Human All Exon V6 Kit (Agilent Technologies) following the vendor’s recommended protocol. Sequencing was performed using the Illumina NovaSeq 6000 with 150-bp paired-end sequencing mode. The transcriptome sequencing, whole-exome sequencing, and its analysis were conducted by Lianchuan Biotech Co., Ltd.

### Lipidomic analysis.

Indicated RKO, RKO EC-R, and RKO EC-R MOGAT3^KO^ cells were collected for lipid extraction, which were then analyzed with a Dionex UltiMate 3000 HPLC system (Thermo Fisher Scientific) equipped with a Q Exactive hybrid quadrupole Orbitrap mass spectrometer (Thermo Fisher Scientific). For the UHPLC-MS/MS analysis, chromatographic lipids were separated using the UHPLC-Q Exactive HF-X Vanquish Horizon system (Thermo Fisher Scientific) by Majorbio. After UPLC-MS/MS analyses, the raw data were imported into LipidSearch (Thermo Fisher Scientific) for peak detection, alignment, and identification. MS/MS fragments identified the lipids. The data were analyzed through the free online central cloud platform (https://www.majorbio.com/).

### RNA interference and lentiviral transfection.

Small interfering RNAs (siRNAs) targeting MOGAT3 were synthesized by Gene Pharma and transfected into the RKO EC-R and HT29 EC-R cell lines with Lipofectamine RNAiMAX (Invitrogen). Stable MOGAT3-overexpressing RKO cells were established using a MOGAT3 overexpression plasmid (Qingke Co. Ltd.). According to the manufacturer’s instructions, lentivirus production and infection were performed with Lipofectamine 3000 (Invitrogen). The CRISPR/Cas9 editing system was employed to knock out MOGAT3 cells in RKO EC-R and HT29 EC-R cells based on the manufacturer’s protocol. Additionally, we generated the PCDH-CMV-MCS-EF1-GFP-Puro vector (Tsingke).

### RNA isolation and quantitative RT-PCR.

Total RNA was extracted from cells using TRIzol reagent. Subsequently, cDNA was synthesized using a cDNA reverse transcriptase kit (Takara). SYBR Green–based quantitative real-time PCR (RT-qPCR) was carried out using the LightCycler 480 real-time PCR system (Roche). The primer sequences are listed in Supplemental Table 1.

### Antibodies and Western blotting.

After being treated with RIPA buffer containing protease inhibitors and phosphorylase inhibitors, protein concentration was determined using a BCA Protein Assay Kit (Beyotime), and then samples were supplemented with DTT (Sigma-Aldrich), sonicated, and boiled for 10 minutes. Equal amounts of protein were resolved in 4%–12% SDS-PAGE gels and then transferred to PVDF membranes (Millipore). The membranes were incubated with the appropriate antibodies. All antibodies were used at the recommended dilution (Supplemental Table 2).

### Oil Red O staining.

Frozen cancer tissues were embedded in OCT compound (Sakura) and cut into 10-μm sections. The sections were washed several times with distilled water, followed by preincubation in 60% isopropanol before being finally stained with a filtered Oil Red O working solution consisting of 60% Oil Red O stock solution (Baso, BA-4081) and 40% deionized water. After a series of washing steps in 60% isopropanol, the nuclei were counterstained with hematoxylin and differentiated in 1% hydrochloric acid in alcohol. Finally, the slides were washed several times with distilled water and sealed with glycerin gelatin. Representative images were captured using an inverted microscope (Olympus).

### Immunofluorescence.

Five thousand cells were plated in each confocal dish. Media were aspirated, and cells were fixed with 2% paraformaldehyde (PFA) in PBS for 10 minutes. The confocal dish was washed twice with 0.1% Triton X-100 in PBS. A blocking solution (2% BSA) was added for 1 hour, followed by primary antibodies (Supplemental Table 2) diluted in the blocking solution at 1:500 and incubated at 4°C overnight. The next day, confocal dishes were washed twice with PBS. Secondary antibodies were FITC-conjugated donkey anti–rabbit IgG, Alexa Fluor 647–conjugated donkey anti–rabbit IgG, and Alexa Fluor 488–conjugated goat anti–mouse IgG1 (Invitrogen/Thermo Fisher Scientific). Cells were mounted with a fluorescence mounting medium containing DAPI (Abcam, ab104135). Immunofluorescence results were imaged using a Zeiss LSM 800 confocal microscope. Data were processed using ZEISS ZEN software.

### Nile red staining.

The tumor tissue slides were seeded on cover glasses and fixed using 4% PFA for 20 minutes at room temperature. Subsequently, Nile red (MCE, HY-D0718) was added at a 1:2000 dilution in PBS for 10 minutes. Afterward, the slides were counterstained with DAPI (MCE, HY-D0814) at a concentration of 1 μg/mL in PBS for 5 minutes at room temperature before imaging. The slides were visualized using a fluorescence microscope (Olympus).

### BODIPY 493/503 staining.

Cells were fixed in 4% PFA for 20 minutes at room temperature and incubated with BODIPY 493/503 (Thermo Fisher Scientific, D3299) at 1:2000 and DAPI in PBS for 15 minutes at room temperature. Finally, the cells were visualized with a fluorescence microscope (Olympus).

### Immunohistochemistry.

Immunohistochemistry (IHC) was performed as previously described (45). Semiquantitative scoring was used to analyze the IHC results. According to the dyeing intensity, the grading was as follows: non-dyeing scored 0, light yellow scored 1, brown/yellow scored 2, and brown scored 3. The mean values of 5 visual fields (×400) were used to calculate the percentage of positive tumor cells in all visual fields. A percentage of positive tumor cells in the visual field of less than 1% scored 0, 1%–25% scored 1, 25%–75% scored 2, and 75%–100% scored 3. The final score was the sum of the dyeing intensity and positive cell scores. Antibodies used here are listed in Supplemental Table 2. All these antibodies were used at the manufacturer’s recommended dilution.

### TUNEL assay.

In situ, paraffin-embedded specimens were tested using a cell death detection kit (Roche, 11684795910), according to the manufacturer’s instructions. Representative TUNEL images were captured using an inverted microscope (Olympus).

### Apoptosis analysis and tumor cell sorting.

The number of apoptotic cells was determined using an Annexin V-FITC apoptosis kit (BD Biosciences), following the manufacturer’s instructions. Cells from different groups were harvested with 0.25% trypsin and washed with PBS. After centrifugation, the cells were resuspended in 100 μL of buffer and stained with 3 μL of Annexin V and 5 μL of propidium iodide (PI). The mixture was incubated in the dark at 4°C for 15 minutes. The cells were sorted using a FACSCalibur flow cytometer (BD Biosciences), and 10,000 cells per sample were counted during the assay. The results were analyzed using Cell Quest software (BD Biosciences). The experiments were repeated 3 times. Briefly, tumors were digested using DNase I (Sigma-Aldrich, D5025) and collagenase type II (STEMCELL Technologies, 07419), followed by treatment with ACK lysis buffer (Gibco, A10492-01). Cells were blocked for 15 minutes on ice with Human TruStain FcX Fc Receptor Blocking Solution (BioLegend, 422301). For flow cytometric analysis of epithelial cells and immune cells, cells were stained for 30 minutes on ice with anti-CD45–PE (1:100; BioLegend), anti-CD326 (anti-EpCAM)–APC-Cy7 (1:100; BioLegend), and Zombie Violet dye (1:200; BioLegend). Cells were resuspended in PBS and analyzed on a CytoFLEX SRT Cell Sorter. Flow gating strategies were kept consistent between samples to enable comparison across cohorts.

### ChIP assay and double luciferase reporter gene experiment.

ChIP was carried out via a ChIP kit (Beyotime, P2080S) according to the manufacturer’s recommended protocol. Antibody and primer sequences are listed in Supplemental Tables 1 and 2. The cells were plated in 24-well plates at a density of 3 × 10^4^ cells per well and then transfected with 0.5 μg of the promoter-luciferase plasmid. Meanwhile, 0.5 μg of pRL-CMV (*Renilla* luciferase) was also transfected to normalize the transfection efficiency. Luciferase activity was measured using a Dual-Luciferase Assay kit (Promega) after 48-hour transfection and a full-wavelength microplate reader (Varioskan Flash, Thermo Fisher Scientific) following the manufacturer’s guidelines.

### Seahorse analysis.

The Seahorse XFe 96 Extracellular Flux Bioanalyzer from Agilent was utilized to measure the oxygen consumption rate (OCR) according to the manufacturer’s protocol. After plating the cells in a 96-well plate for 24 hours, cells were placed in fresh DMEM containing 10 mM glucose, 2 mM L-glutamine, and 1 mM sodium pyruvate and incubated for 1 hour. To each well, 3 metabolic inhibitors were added sequentially, namely oligomycin (Oligo; 1 μM), followed by carbonyl cyanide 4-trifluoromethoxy-phenylhydrazone (FCCP; 2 μM), and then rotenone (Rot; 2 μM).

### FAO assay.

An FAO assay was conducted following the protocol provided by Abcam (ab222944). In brief, approximately 6 × 10^4^ cells were seeded into 96-well plates, and positive controls were treated with 2.5 μM FCCP, while negative controls were treated with 40 μM etomoxir. Rates of FAO were calculated by determining the slopes (*m*) from the linear portion of each profile and using the formula provided by Abcam.

### Statistics.

Data are presented as the mean ± SEM of 3 independent experiments. Comparisons were analyzed using a 2-tailed, unpaired *t* test or 1-way ANOVA with Tukey’s multiple-comparison test or 2-way ANOVA with Tukey’s multiple-comparison test. Statistical analyses were conducted using GraphPad Prism version 9.0. or SPSS Statistics software. A *P* value of less than 0.05 was considered significant.

### Study approval.

The Medical Ethical Board of the Sir Run Run Shaw Hospital, School of Medicine, Zhejiang University approved the collection and use of human tumor tissue for the PDX model (study number 20220209-93). All animal procedures were conducted strictly within institutional guidelines and were approved by the Medical Ethical Board of the Sir Run Run Shaw Hospital, School of Medicine, Zhejiang University (SRRSH202202112).

### Data availability.

Raw data are accessible in NODE (https://www.biosino.org/node) with the accession number OEP00005624 or through the URL https://www.biosino.org/node/project/detail/OEP00005624 All data reported in this work are available in the Supporting Data Values file.

## Author contributions

ZS contributed to conceptualization and methodology. JW, HW, WZ, XL, HW, QM, JC, and XC performed experiments. JW, HW, WZ, and ZS performed data, bioinformatic, and statistical analyses. ZS, WZ, and YJ provided reagents and resources. JW, HW, WZ, YL, DWC, and ZS contributed to manuscript drafting and editing. ZS and ZJ provided guidance and supervision. ZS, WZ, and HW acquired funding for the study. All authors reviewed the manuscript.

## Supplementary Material

Supplemental data

Unedited blot and gel images

Supporting data values

## Version 1 10/22/2024

In-Press Preview

## Version 2 12/16/2024

Electronic publication
