# Supplementary material for: MOGAT3-mediated DAG accumulation drives acquired resistance to anti-BRAF/anti-EGFR therapy in BRAFV600E-mutant metastatic colorectal cancer
Source: J Clin Invest. 2024 Oct 22;134(24):e182217. doi: 10.1172/JCI182217 (PMC11645146; doi:10.1172/JCI182217)
Supplement: Supplemental data [file jci-134-182217-s085.pdf]

Supplemental material

MOGAT3-Mediated DAG Accumulation Drives Acquired Resistance to Anti-BRAF/EGFR Therapy in *BRAF*^{V600E}-Mutant Metastatic Colorectal Cancer

Jiawei Wang[1,2,3,6], Huogang Wang[1,2,3,6], Wei Zhou[1,2,3], Xin Luo[1], Huijuan Wang[1,2,3], Qing Meng[1,2,3], Jiaxin Chen[1], Xiaoyu Chen[1,2,3], Yingqiang Liu[1,2,3], David W Chan[4], Zhenyu Ju[5], Zhangfa Song[1,2,3#]

[1] Department of Colorectal Surgery, Sir Run Run Shaw Hospital, School of Medicine, Zhejiang University, Hangzhou, Zhejiang, 310016, P.R. China.

[2] Key Laboratory of Biological Treatment of Zhejiang Province, 310016, Hangzhou, P.R. China

[3] Key Laboratory of Integrated Traditional Chinese and Western Medicine Research on Anorectal

Diseases of Zhejiang Province,310016, Hangzhou, P.R. China.

[4] School of Medicine, The Chinese University of Hong Kong, Shenzhen, Guangdong, 518172, P.R. China.

[5] Key Laboratory of Regenerative Medicine of Ministry of Education, Institute of Aging and Regenerative Medicine, Jinan University, Guangzhou, P.R. China

[6] These authors contributed equally: Jiawei Wang, Huogang Wang.

#Correspondence should be addressed to:

Prof. Zhangfa Song, Department of Colorectal Surgery, Sir Run Run Shaw Hospital,

Zhejiang University School of Medicine, Key Laboratory of Biological Treatment of Zhejiang Province, Key Laboratory of Integrated Traditional Chinese and Western Medicine Research on Anorectal Diseases of Zhejiang Province, 310016, Hangzhou, China. Phone: (86) 571-86006355; E-mail: songzhangfa@zju.edu.cn

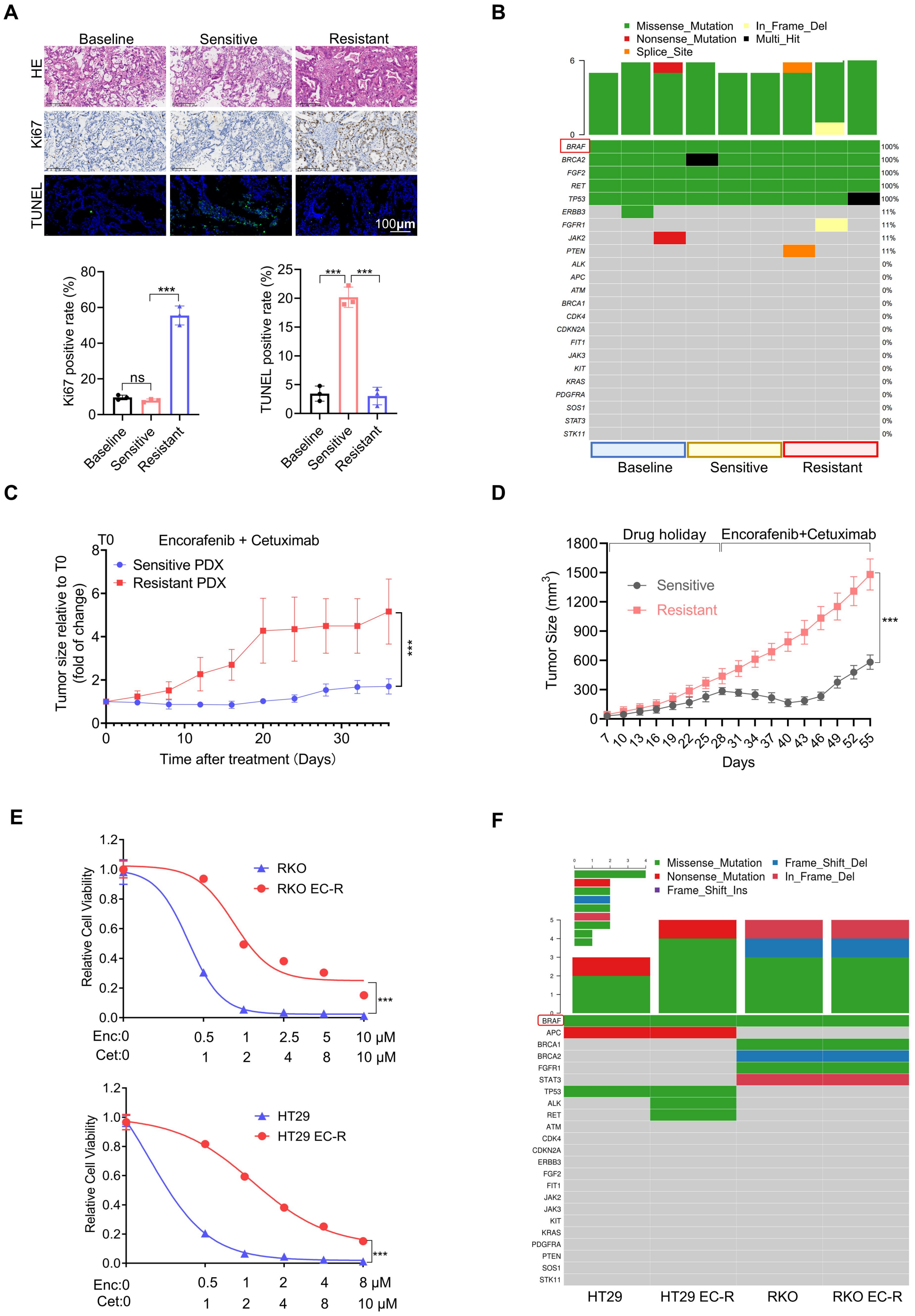

Supplemental Figure 1

A. Representative images of HE, Ki67, and TUNEL staining of baseline, sensitive, and resistant tumor tissues. The Ki67 and TUNEL staining levels were quantified (n=3).

B. Whole-exome sequencing reveals gene mutations in PDXs from baseline, sensitive, and resistant periods (n=3).

C. The fold changes of mean tumor volumes (±SEM) relative to baseline (T0) for implanted sensitive (n=6) and resistant tumor tissues (n=3) in nude mice upon encorafenib and cetuximab treatment.

D. Xenograft tumor growth in nude mice inoculated with sensitive/resistant *BRAF*^{V600E}-Mutant mCRC tumor tissues. PDXs were treated with encorafenib+cetuximab (n=6).

E. Cell viability of RKO, HT29, and RKO EC-R, HT29 EC-R cells was assessed upon increasing concentrations of encorafenib/cetuximab for 3 days (n=3).

F. Whole-exome sequencing reveals gene mutations in RKO, HT29, RKO EC-R, and HT29 EC-R cells.

The data were presented as the mean ± SEM of three independent experiments, ns, no significance; * $p < 0.05$, **$p < 0.01$, and ***$p < 0.001$. (1-way ANOVA with Tukey's multiple-comparison test in A; 2-way ANOVA with Tukey's multiple-comparison test in C, D and E).

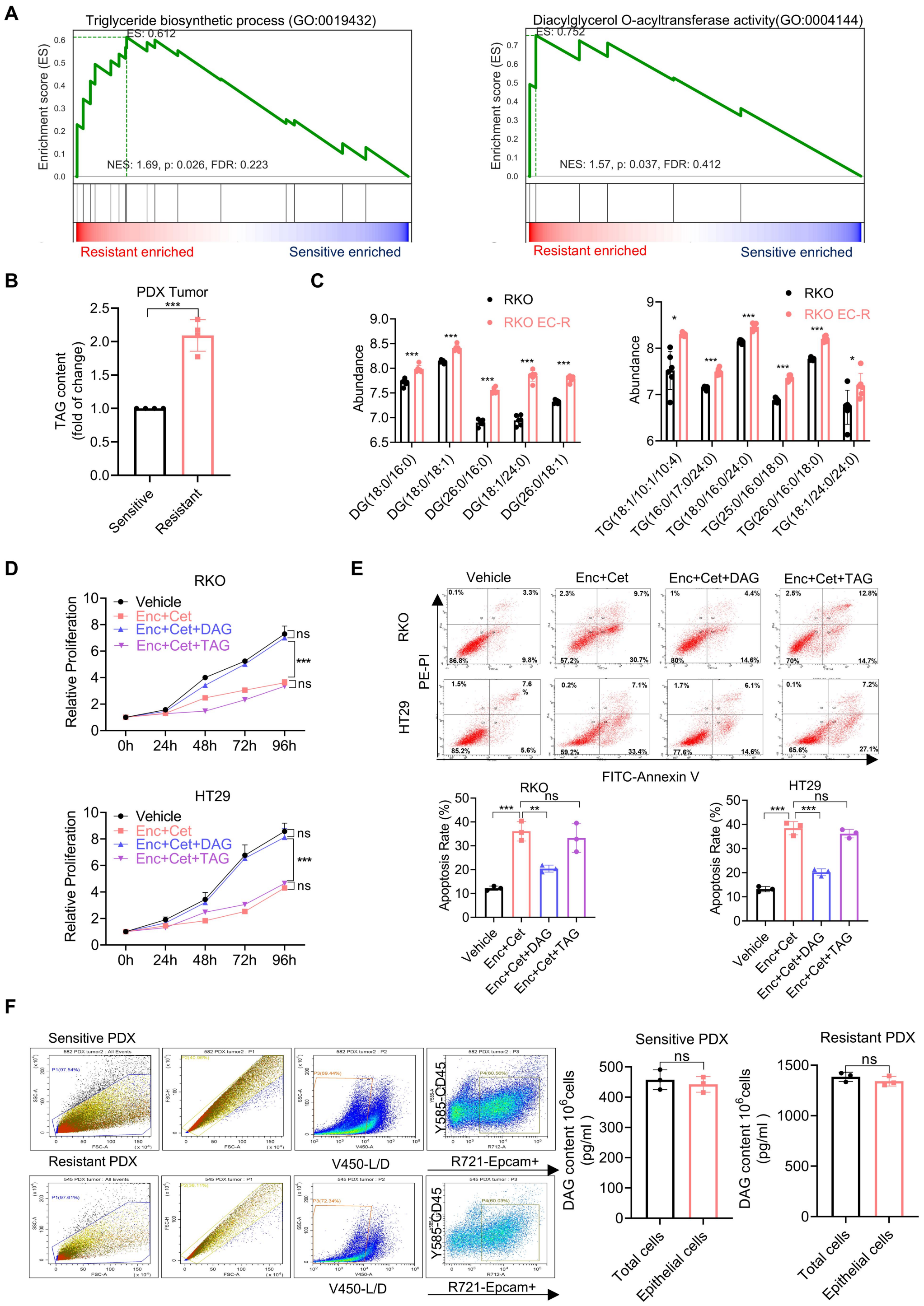

Supplemental Figure 2

A. Gene set enrichment analysis (GSEA) of resistant tumors versus sensitive tumors (n=3) showed enhanced diacylglycerol O-acyltransferase activity and triglyceride biosynthetic process. Normalized enrichment score (NES) and nominal *p value (p)*

were provided according to GSEA analysis.

B. The TAG level in sensitive and resistant PDX tumors (n=4).

C. The DAG and TAG levels were quantified in RKO and RKO EC-R cell lines (n=6).

D. RKO and HT29 cell lines were treated with encorafenib(0.25μM)-cetuximab (0.5μM) or combined with DAG, TAG (10μM) for 96 h. Relative OD value was assessed, representing the cell viability by CCK-8 assays (n=3).

E. RKO, HT29 cell lines were exposed to (DMSO, vehicle), encorafenib (0.25μM)-cetuximab(0.5μM) or encorafenib (0.25μM)-cetuximab (0.5μM) + DAG, TAG (10μM) treatment for 48h. The representative image of apoptotic rates of the indicated RKO HT29 cell lines was assessed by flow cytometry (up). The quantification of the apoptosis rate is shown (down) (n=3).

F. Tumor epithelial cells were flow-sorted from sensitive and resistant PDX tissues, and the DAG content in both total cells and isolated epithelial cells was measured using an ELISA kit (n=3).

The data were presented as the mean ± SEM of three independent experiments, ns, no significance; * $p < 0.05$, ** $p < 0.01$, and *** $p < 0.001$. (2-tailed unpaired t test in B, C and F; 1-way ANOVA with Tukey's multiple-comparison test in E, 2-way ANOVA with Tukey's multiple-comparison test in D).

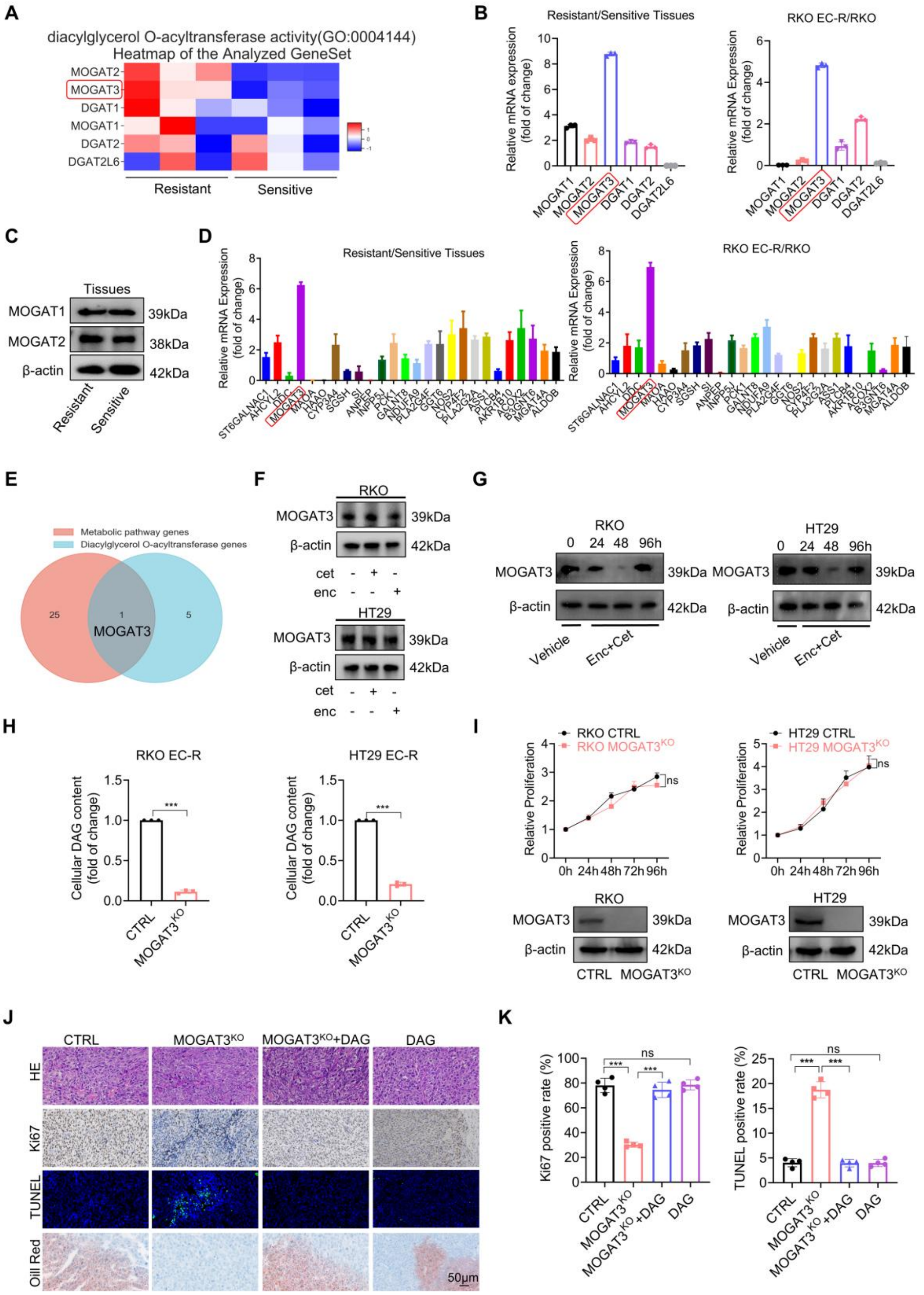

A
diacylglycerol O-acyltransferase activity(GO:0004144)
Heatmap of the Analyzed GeneSet
MOGAT2
MOGAT3
DGAT1
MOGAT1
DGAT2
DGAT2L6
Resistant
Sensitive
B
Resistant/Sensitive Tissues
RKO EC-R/RKO
Relative mRNA expression (fold of change)
Relative mRNA Expression (fold of change)
MOGAT1
MOGAT2
MOGAT3
DGAT1
DGAT2
DGAT2L6
C
Tissues
MOGAT1
39kDa
MOGAT2
38kDa
β-actin
42kDa
Resistant
Sensitive
D
Resistant/Sensitive Tissues
RKO EC-R/RKO
E
Metabolic pathway genes
Diacylglycerol O-acyltransferase genes
25
1
5
MOGAT3
F
RKO
MOGAT3
39kDa
β-actin
42kDa
cet
enc
HT29
G
RKO
HT29
0 24 48 96h
Vehicle
Enc+Cet
H
RKO EC-R
HT29 EC-R
Cellular DAG content (fold of change)
CTRL
MOGAT3KO
I
RKO CTRL
RKO MOGAT3KO
HT29 CTRL
HT29 MOGAT3KO
Relative Proliferation
0h 24h 48h 72h 96h
ns
J
CTRL
MOGAT3KO
MOGAT3KO+DAG
DAG
HE
Ki67
TUNEL
Oill Red
50μm
K
Ki67 positive rate (%)
TUNEL positive rate (%)
CTRL
MOGAT3KO
MOGAT3KO+DAG
DAG
ns

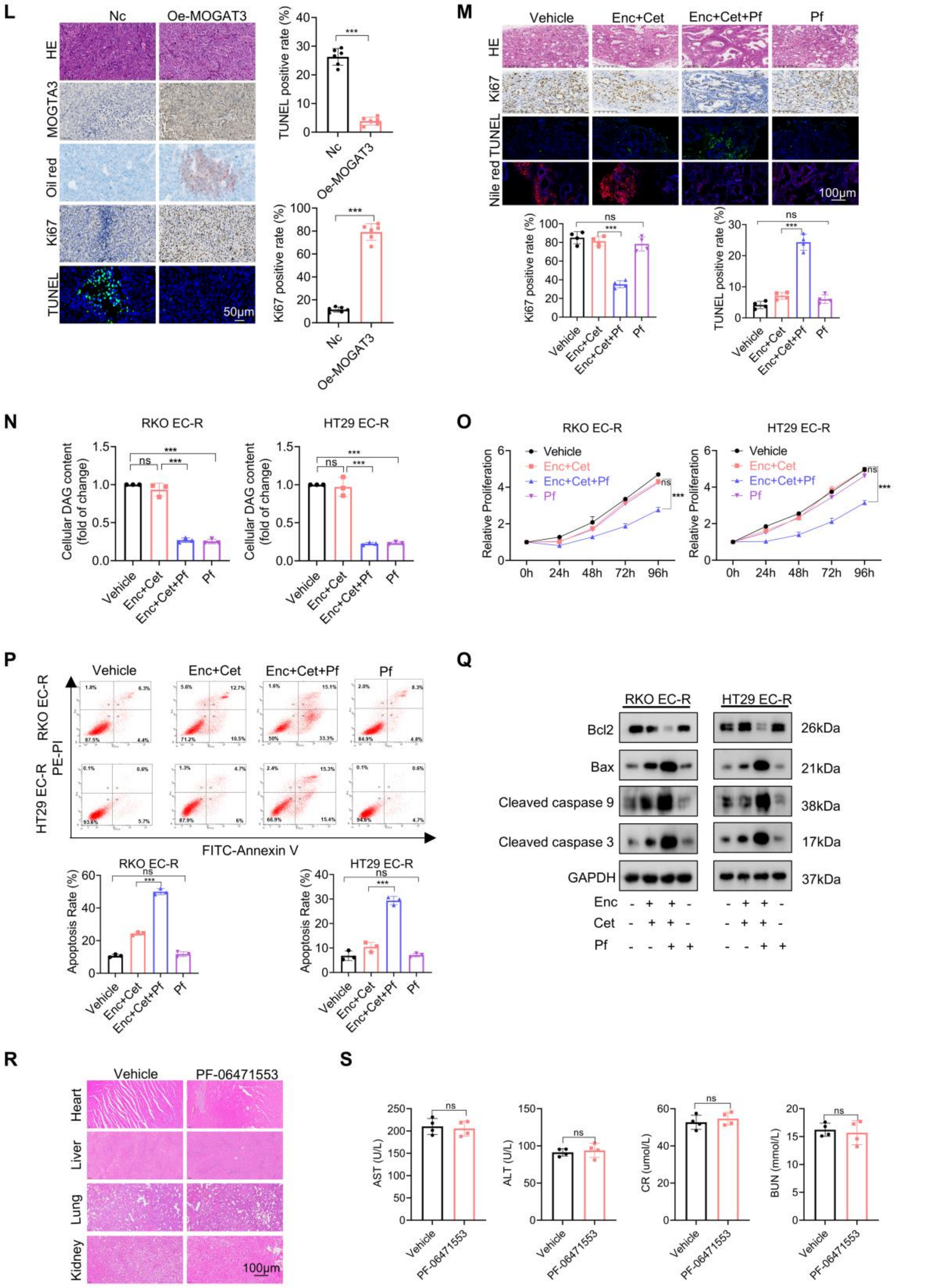

Supplemental Figure 3

A-B. Heatmap of analyzed gene sets related to Fig.S2A (right), and RT-qPCR showed folds of change on the mRNA levels of gene sets in resistant versus sensitive PDXs and RKO EC-R versus RKO cell lines (n=3).

C. Western blot assessed MOGAT1,2 proteins in resistant and sensitive tissues. A representative blot was shown from three independent experiments.

D-E. RT-qPCR showed folds of change on the mRNA levels of genes of Fig.1D in resistant versus sensitive PDXs and RKO EC-R versus RKO cell lines (n=3). Venn diagram showing the intersection of both genes (E).

F. Western blot evaluated MOGAT3 proteins under encorafenib or cetuximab treatment in RKO and HT29. A representative blot was shown from three independent experiments.

G. Western blot was used to detect MOGAT3 protein expression at different time points after combined treatment with encorafenib and cetuximab in RKO and HT29 cells. A representative blot was shown from three independent experiments.

H. The DAG content in RKO EC-R MOGAT3^{KO} and HT29 EC-R MOGAT3^{KO} cells (n=3).

I. Western blot evaluated MOGAT3 proteins in RKO MOGAT3^{KO} and HT29 MOGAT3^{KO} cells. Relative OD value was assessed, representing the cell viability by CCK-8 assays (n=3).

J-K. Representative images of HE, Oil red, TUNEL, and Ki67 related to Fig.3C (J) and quantification (K) (n=4).

L. Representative images of HE, TUNEL, Oil red, Ki67 and IHC of MOGAT3 related to Fig.3F and quantification (n=6).

M. Representative images of HE, Ki67, Nile red, and TUNEL related to Fig.3I and quantitation (n=4).

N. The DAG content in RKO EC-R and HT29 EC-R cells was tested after being exposed to vehicle (DMSO, vehicle), encorafenib(2μM)-cetuximab(4μM), or encorafenib-cetuximab+pf or pf (10μM) 48h (n=3).

O. After vehicle (DMSO), pf (10μM), encorafenib(2μM)-cetuximab(4μM) or pf (10μM) + encorafenib(2μM)-cetuximab (4μM) treatment in RKO EC-R and HT29 EC-R cells. Relative OD value was assessed to determine cell viability by the CCK-8 assay (n=3).

P. Apoptotic rate of the indicated RKO EC-R and HT29 EC-R cell lines were assessed by flow cytometry after vehicle, encorafenib(2μM)-cetuximab(4μM), encorafenib-cetuximab + pf or pf(10μM) treatment for 48 h representative images of apoptotic rates and quantification (down panel) (n=3).

Q. Western blotting to detect the apoptotic proteins in treated cell lines, a representative blot was shown from three independent experiments related to Fig.S3P.

R. Representative histopathology of liver, kidney, heart, and lung collected from rats treated with pf (100mg/kg/day) intraperitoneal injection for two weeks.

S. AST, ALT, BUN, and CR levels in serum were quantified after treatment as in Fig.S3R (n=4).

The data were presented as the mean ± SEM of three independent experiments, ns, no significance; *$p < 0.05$, **$p < 0.01$, and ***$p < 0.001$. (2-tailed unpaired t test in H, L and S; 1-way ANOVA with Tukey's multiple-comparison test in B, D, K, M, N and P; 2-way ANOVA with Tukey's multiple-comparison test in I and O).

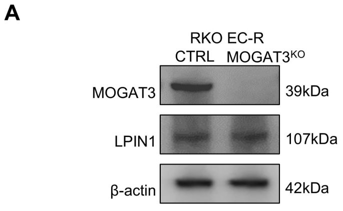

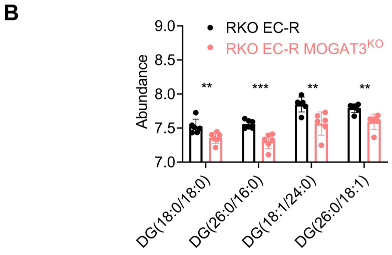

Supplemental Figure 4

A. Western blot showed protein levels of MOGAT3 and LPIN1 following knockout MOGAT3 in RKO EC-R cells. A representative blot was shown from three independent experiments.

B. DAG level in RKO EC-R cells after knocking out MOGAT3 (n=6).

The data were presented as the mean ± SEM of three independent experiments, ns, no significance; **p* < 0.05, ***p* < 0.01, and ****p* < 0.001. (2-tailed unpaired t test in B).

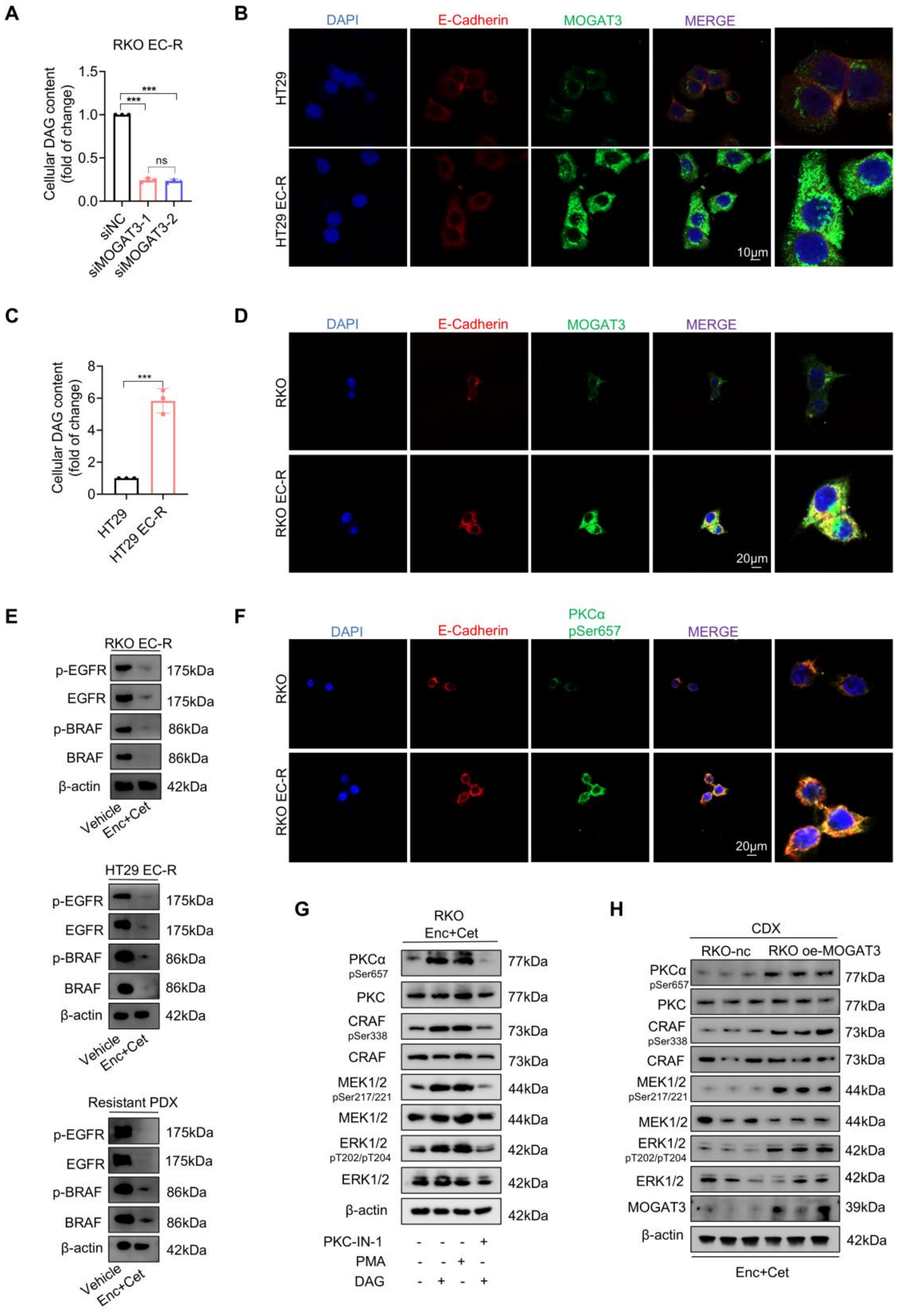

Supplemental Figure 5

A. The DAG content in RKO EC-R cells was tested after si-RNA-MOGAT3 treatment at 48h (n=3).

B. Immunofluorescence of MOGAT3 signaling in HT29 and HT29 EC-R cells.

Representative images were shown. Scale bar, 10μm.

C. The DAG content in HT29 and HT29 EC-R cells (n=3).

D. Immunofluorescence of MOGAT3 signaling in RKO and RKO EC-R cells. Representative images were shown. Scale bar, 20μm.

E. Western blot detected BRAF/EGFR signaling in RKO EC-R/ HT29 EC-R cells and resistant PDX tumors after BRAF/EGFR inhibitors.

F. Immunofluorescence of phospho-PKCα signaling in RKO and RKO EC-R cells. Representative images were shown. Scale bar, 20μm.

G. Western blot detected PKCα/CRAF and MEK/ERK signaling in RKO cells treated with PMA (10μM), DAG (10μM) or a combination of DAG (10μM) and PKCα-inhibitor (10μM) for 24 hours.

H. Immunoblot analysis of MOGAT3, PKCα/CRAF, and MEK/ERK signaling in RKO nc and oe-MOGAT3 cells derived Xenograft (CDX) tumors from Fig.3F.The tumor tissues were harvested for western blotting to detect the indicated signaling proteins. A representative blot was shown from three independent experiments.

The data were presented as the mean ± SEM of three independent experiments, ns, no significance; *$p < 0.05$, **$p < 0.01$, and ***$p < 0.001$. (2-tailed unpaired t test in C; 1-way ANOVA with Tukey's multiple-comparison test in A).

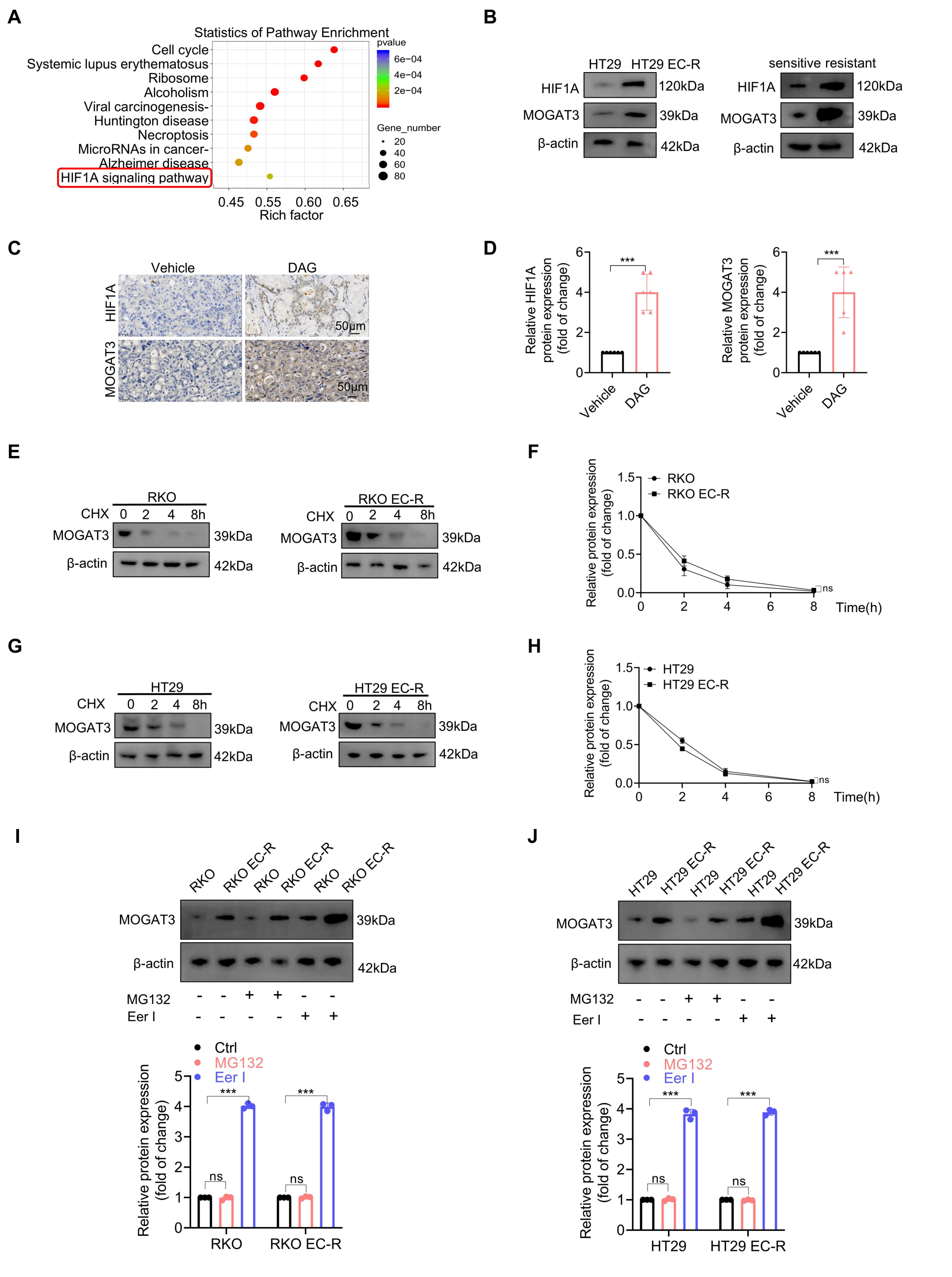

Supplemental Figure 6

A. Enrichment of KEGG pathways showed that the HIF1A signaling pathway was activated in resistant PDX tumors (n=3).

B. Immunoblot analysis of MOGAT3 and HIF1A in HT29 EC-R and resistant PDXs.

C-D. Representative images of HIF1A and MOGAT3 in PDX tissues related to Fig.2C and Quantitative analysis (D) (n=6).

E-F. RKO/RKO EC-R cells were treated with 50 μM cycloheximide (CHX) for the indicated time (E) respectively, followed by western blotting test, and the band intensity was quantified (F) (n=3).

G-H.HT29/HT29 EC-R cells were treated with 50 μM cycloheximide (CHX) for the indicated time (G) respectively, followed by western blotting test, and the band intensity was quantified (H) (n=3).

I-J.RKO/RKO EC-R (I) and HT29/HT29 EC-R cells (J) were treated with proteasome inhibitor MG132 (10 μM) and ERAD inhibitor eeyarestatins (Eer I, 20 μM) for the indicated time followed by western blotting test and the band intensity was quantified (n=3).

The data were presented as the mean ± SEM of three independent experiments, ns, no significance; *$p < 0.05$, **$p < 0.01$, and ***$p < 0.001$. (2-tailed unpaired t test in D; 1-way ANOVA with Tukey's multiple-comparison test in I and J; 2-way ANOVA with Tukey's multiple-comparison test in F and H).

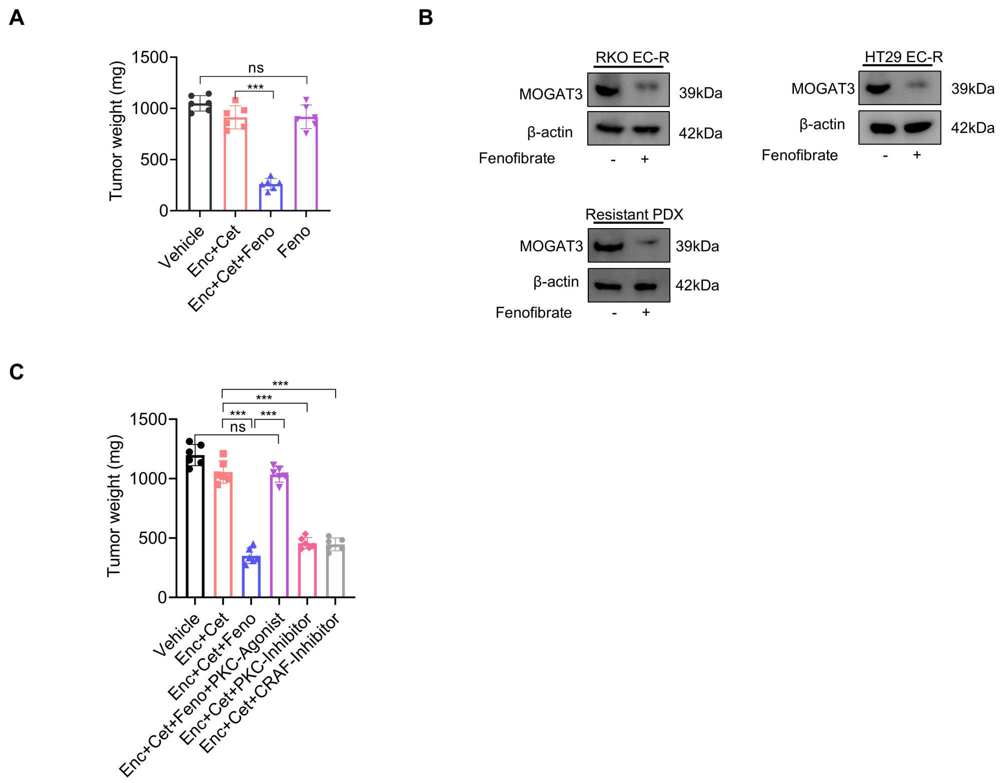

Supplemental Figure 7

A. Quantitation of xenograft tumor weight related to Fig.7A (n=6).

B. Immunoblot analysis of MOGAT3 in RKO EC-R/HT29 EC-R cells and resistant PDXs after fenofibrate treatment.

C. Tumor weight quantification related to Fig.7E (n=6).

The data were presented as the mean ± SEM of three independent experiments, ns, no significance; *$p < 0.05$, **$p < 0.01$, and ***$p < 0.001$. (1-way ANOVA with Tukey's multiple-comparison test in A and C).

Table S1. Primers Gene Species Forward Reverse

Gene	Species: Human	
	Forward	Reverse
ACOX2	CGCCTGGGTTGGTTAGAAGAT	CTGAGGGCTCTCACGAAGAC
AHCYL2	TTCAACAAACGTCCCACCAAA	CCTGGGCGATGTCTCATCA
AKR1B10	GTGACACCAGCACGCATTG	GCATTGAAGGGATAGTCTTCCAA
ALDOB	TGTCTGGTGGCATGAGTGAAG	GGCCCGTCCATAAGAGAAACTT
ANPEP	TTCAACATCACGCTTATCCACC	AGTCGAACTCACTGACAATGAAG
ASS1	CTTGGGGCCAAAAAGGTGTTC	GAGGTAGCGGTCCTCATACAG
B3GNT6	GTGCGCCGCCTCTTTCTATT	CCAGCCAGTCGAGCAAGTG
CYP3A4	CACGAGCAGTGTTCTCTCCTT	CACAGTATCATAGGTGGGTGGT
CYP4F2	GAGGGTAGTGCCTGTTTGGAT	CAGGAGGATCTCATGGTGTCTT
DDC	ATTCATCTGCCCTGAGTTCCG	CCAATAGCCATTTGTGGGGAT
DGAT1	TATTGCGGCCAATGTCTTTGC	CACTGGAGTGATAGACTCAACCA
DGAT2	ATTGCTGGCTCATCGCTGT	GGGAAAGTAGTCTCGAAAGTAGC
DGAT2L6	TTCTCCCGACTGAATCTCCAG	GTTCCAATCATAGGTGAGCCAG
FOSL2	CAGAAATTCCGGGTAGATATGCC	GGTATGGGTTGGACATGGAGG
GALNT8	GACACGCGAGACTACAGATGT	GATGGCCCGTTGTATAATGGAC
GGT6	AATTCCACGGCCCTGACATC	CCATCAGCATGGCAAAGTAGT
HAAO	AGATGGGCTCAGGTACTATGTG	CCGAGGTCCTTGCAGTAGAA
INPP5J	GCGCAGACATGATCGCCATA	CCTCACCGAACTCACCAGC

MAOA	GAATCAAGAGAAGGCGAGTATCG	GGCAGCAGATAGTCCTGAAATG
MGAT4A	AAAATCCATGTAAACCCACCTGC	AGTCTCCAGCTATCGGTGTGA
MOGAT1	AAAGTGTGTCCTACATGGTAAGC	TGATCCTTCAGGGTTGTCAGTT
MOGAT2	ACACTTGCTGTCCTACAGTTTG	GAGGAGCCAGAATCTTGTAAACA
MOGAT3	CCAACCACTTCCAAAACCTTGC	TGCCCGGTTCCTTATCCACT
NDUFA9	CCGACGAGTAGTACAACACAGC	GCTTCCTTGGACAGTTGAGCA
NOS2	TTCAGTATCACAACCTCAGCAAG	TGGACCTGCAAGTTAAAATCCC
PCK1	TTGAGAAAGCGTTCAATGCCA	CACGTAGGGTGAATCCGTCAG
PLA2G2A	ATGAAGACCCTCCTACTGTTGG	GCTTCCTTTCCTGTCGTCAACT
PLA2G4F	GAGACCTTCCACTACCAGATCC	TTGGTGATGACTTCAGATGCAG
PLCB4	TATTCGGTCGGGAGCCATAC	GACACAAACTATCCGCCCTTC
SGSH	GGGGCGGAACATCACTAGAAT	TGGAAGGCGACGTAGAGGAA
SI	AAATCAGACACCCAATCGTTTCC	GGGCAACCTTCACATCATACAA
ST6GALNAC1	CACAGCCAAGACGCTCATTC	CCTTTCTGTCTCGTCCTTGTTG
***PKCα*-si**	GCGUCCUGUUGUAUGAAAUTT	AUUUCAUACAACAGGACGCTT
***CRAF*-si**	GAGAGAUUCAAGCUAUUAUTT	AUAAUAGCUUGAAUCUCUCTT
**chip-*MOGAT3***	GACTTCAAGCAAGAGAGGGACA	GCTTTTCTTTCGGCGCCAT
***MOGAT3*-si1#**	GAGACAACUAAGGGAUUAUTT	AUAAUCCCUUAGUUGUCUCTT
***MOGAT3*-si2#**	GGCUUCCUCUGUAAUUUCUTT	AGAAAUUACAGAGGAAGCCTT
sg.RNA	CTTCTGCAAGGTTTTGGAAG	

Table S2. Antibodies and compounds

REAGENT or RESOURCE	SOURCE	IDENTIFIER
Antibodies		
Rabbit HIF1-alpha antibody	Abcam	ab308433,<br>RRID:AB_2941086
Rabbit Anti-CPT1A antibody	Abcam	ab128568,<br>RRID:AB_11141632
Rabbit anti-MOGAT3	Affinity	DF9099,<br>RRID:AB_2842295
Rabbit anti-Phospho-C-RAF (Ser338)	Affinity	AF3065,<br>RRID:AB_2834492
Rabbit anti-Cleaved-Caspase9(Asp353)	Affinity	AF5240<br>RRID:AB_2837726
Rabbit anti-Cleaved-Caspase3(Asp175,p17)	Affinity	AF7022<br>RRID:AB_2835326
Rabbit anti-Bcl-2	Affinity	AF6139,<br>RRID:AB_2835021
Rabbit anti-eIF4E	Affinity	AF6110,<br>RRID:AB_2834997
Rabbit anti-Phospho-eIF4E	Affinity	AF3110,<br>RRID:AB_2834547
Rabbit anti-Bax	Affinity	AF0120,<br>RRID:AB_2833304
Rabbit anti-MOGAT1	Affinity	DF15824,<br>RRID:AB_2941087
Rabbit anti-MOGAT2	proteintech	19514-1-AP,

		RRID:AB_10638917
Rabbit anti-Phospho-PKC Alpha (Ser657)	proteintech	28926-1-AP,<br>RRID:AB_2918214
Rabbit anti-PKC Alpha	proteintech	21991-1-AP,<br>RRID:AB_2878965
Rabbit anti-Phospho-ERK1/2 (Thr202/Tyr204)	proteintech	28733-1-AP,<br>RRID:AB_2881202
Rabbit anti-ERK1/2	proteintech	11257-1-AP,<br>RRID:AB_2139822
Rabbit anti-LPIN1	proteintech	27026-1-AP<br>RRID: AB_2880727
Mouse anti-HIF1-alpha	proteintech	66730-1-Ig,<br>RRID:AB_2882080
Rabbit anti-E-Cadherin	Cell Signaling Technology	3195 RRID: AB_2291471
Rabbit anti-Phospho-MEK1/2 (Ser217/221)	Cell Signaling Technology	9154S, RRID:AB_2941088
Rabbit anti-β-Actin (D6A8)	Cell Signaling Technology	8457S, RRID:AB_2941089
Rabbit anti-GAPDH (14C10)	Cell Signaling Technology	2118S, RRID:AB_2941090
Rabbit anti-EGER	Cell Signaling Technology	4267, RRID:AB_2246311
Rabbit anti- Phospho-EGFR	Cell Signaling Technology	3777, AB_2096270
Rabbit anti-BRAF	Cell Signaling Technology	14814, RRID:AB_2750887

Rabbit anti-Phospho-BRAF	Cell Signaling Technology	2696, RRID:AB_390721
DAG	Sigma	24529-88-2
Encorafenib	MCE	HY-15605
Cetuximab	MCE	HY-P9905
PMA	MCE	HY-18739
RAF-IN-1	MCE	HY-144271
PKC-IN-1	MCE	HY-16903
Tomivosertib	MCE	HY-100022
pf-06471553	MCE	HY-108339
Fenofibrate	MCE	HY-17356
Eeyarestatin I	MCE	HY-110078
MG132	MCE	HY-13259
Cycloheximide	MCE	HY-12320
TAG	Sigma	1716-07-0

***MOGAT3* promoter sequence (2000bp):**

>NC_000007.14:c101196885-101194886 Homo sapiens chromosome 7, GRCh38.p14 Primary Assembly

CAGCCTATTTATTTTTTGAGACAAAGTCTCACTCTGTCACCCAGACTGAAGTGCAGTGGTGTGATCTTAG

CTCACTGCAACCTCTGCCTCCCAGGTTCAAGCGATTCTTGTGCCTCAGCCTCTGTAGTAGCTGGAATTAC

AGGTACCCACTTCCACACCTGGCTAATTTTTATGTTTTTGCAGAGACGGGGGTTTCACCATGTTGGCCAG

GCTGGTCTCCAACTCCTGACCTCAAATGATCCTCCCACCGTGGCCTCCCAAAATGCTGGGATTACAGGTG

TGGTTACAAGACCCTCTCTCTAAAACAAAGAAAAAGAAAGAGAAAGAAATGCGAGTCCTGTCTTCTAGAT

CTTTCTTTGTAGCCATAGTCATGTGCTTTGAATGACCCTGGGAGGTAGCATCTGGACGTAGTGGGAAGGA

CTTGTAGCCCCTGGGGGTGCCCATCTCGGAGCAGTCCAGCAGCCTGAGCCCCCTCTTCTCTCTTGCTCCT

TCCCAGGGCGTCCCTGGTGCCCGTGTACTCCTTTGGGGAGAATGACATCTTTAGACTTAAGGCTTTTGCC

ACAGGCTCCTGGCAGCATTGGTGCCAGCTCACCTTCAAGAAGCTCATGGGCTTCTCTCCTTGCATCTTCT

GGGGTCGCGGTCTCTTCTCAGCCACCTCCTGGGGCCTGCTGCCCTTTGCTGTGCCCATCACCACTGTGGG

TGAGTGCCCACCTCCGGGGGGACGGCCACCAGCAGCTGCGTGGGCATCAGGGATCCCTCGCCCACCTGTC

TCCCTCTCCCTGCAGTGGGCCGCCCCATCCCCGTCCCCCAGCGCCTCCACCCCACCGAGGAGGAAGTCAA

TCACTATCACGCCCTCTACATGACGGCCCTGGAGCAGCTCTTCGAGGAGCACAAGGAAAGCTGTGGGGTC

CCCGCTTCCACCTGCCTCACCTTCATCTAGGCCTGGCCGCGGCCTTTCGCTGAGCCCCTGAGCCCAAGGC

ACTGAGACCTCCACCCACTGTGGACTCCATGCCTCCAATAAAAGGTAGTTCTGGGCCCAGCGCAGTGCCT

CATGCCTGTAATCCCAGCACTTTGGGAGGCCAAGGTGGGAGGATCGTTTGAGCCCAGGAGTTGAAGACCA

GCCTGGGCAACACAGTGAGACTTCATTTCTACAAAAATTAAAAAATAATGTTGTTAATTAGCCGGGCATG

GTGGCATGTGCCTGTAATCCCAGCTATTTGGGAGGCTGAGGCAGGAGACTTGCTTAAACCCAGGAGGCGG

AGGTTGCAGTGAGCTGAGATCACACCACAGTACTCCAGCCTGGGCAACACAGCGAGACTCAATCTCAAAA

AAAAAAAAAAAAAAAAAAAAAAGACTGTTAAAGCTGTAGTAGGCTGGGCGTGGTGGCTCATGCCCATAAT

CCCAACACTTTGGGAAGCCGAGGCAGGTGGATCAACTGAGGTCAGGAGTTCAAGACCAGCCTGGCCAAAA

TGGCAAAACCCCCTCTCTACTAAAACTACAAAAATTAGCCGGGCATGGTGGCTCATGCCTATAATCCCAG

CTACTCCGGAGGCTGAGGCAGGAGAATCGCTTGAACCCAGGAGGCAGAGGTTGCAGTGAGCCGAGATCAC

ACCACTGCACTCCAGTCTGGGCAACAGAGCGAGATTCCATCTCAAAAAAAAAAAAAATGTTGTTAAAGCTA

TAATCGTTCTGGAAGTGAACATGGCCAGGCATGACCTGCCCCCATCCTGATGTGCTACAAAGCTGCTGCC

TTAGATTGGGTATCAGGACCCTGAACTGTCCTCCCAGTGAGAGTTCAGTCTGTGGCATGACCTTGGACAA

GCCTCTTCCTTTTGCCTGTTTACTTCTTTGTCTGTAAATAAAGGCATCTGCTTAGCAAAGAGCTGTTGGG

CATTGGGATGGAGTTAGGAGGGAGGATGCGTGGAGAGGCAGGCTTTGAAGAGTGAGCAGGATTTAGATGG

AGGTGAAGTTAGGGTAGATGGCGGAATTCCAAGAGCTAAA

MOGAT3 promoter wt, >NC_000007.14: mut1, mut2, mut3

>NC_000007.14:c101203036-101201037 Homo sapiens chromosome 7, GRCh38.p14 Primary Assembly

CCCACCATCCTCTTCCCAGGGGACACTGGGGCCCAGCTCTCCTTTCCCCATGTCCTCTCCACTCTTCTTT

CCAAGTCCCAGGCTTTGGCTGGGTGCGGTGGTTCACACCTGTAATCCCAGCACTTGGGAGGCGCAGGCGA

GAGGATCGCTTAAGGCCAGGAGTTCAAGACCAGCCTGGGCAACACAGTGAAACCCCATCTCTACACAAGA

AAAAACAAAAACAAAAATTTAGCCAGGTGTGGTGGTACACACCTGTGGTCCCAGCTACTGGGAGGCTTGA

GGTGGGAGGGTCACTTGAGCCCTGAAGGTCAAGGATGCAGTGAACCAAGATCAGGCCACTGTACTCCAGC

CTGGGCAACAGAGCAAGACTCCGTCCCTAAATAAATAAGTAAATAAGACCCAGGCGTTGGGTCCCCCAGC

TTCTGCCCTCCCCCCAGGTTTGGTCTCCTTTGTCAAGGCCCCGCTGCCTCAGTGGTGGCCAGGTGGCTGT

CCTGGCGTGGGAGGGCCCCTGCAGGCGCTGGAGGCAAAACCCGGACAACTGAGCTTGCCGATTCGGAATC

AGAAGAGATTGGTTAAGTCAGCTCTGGAACTCGGGTGAGGACCCGAGCGTGACCCGGTGGGGACGCCCCA

GGGCTGCCGGGCCGGGGAGGAGGAGAGTGGCGCCACCCGCGGACCCCGGGGCCAGGAGTCAGCCGCGCCA

TGCTCTCCTGCGGCCTCCCTAGTGCCTGTCTTCTCCAGGGAGAATGAGCTCTTCCAGCAGTTCCCGAACC

CGCAGAGCTCGTGGGTGCAGAGGACGCAGGAGGCTCTGCGTCCGCTGCTAAGCGTGGCCCTGCAGCTGTT

CCTGGGCCGCCGGGGCCTCCCGCTGCCCTTCCGCGCGCCCATCCGCACCGTAGGTGAGCCCCGGCCTCCG

CCCCGCGATCCTGGCCCAGGGCCCGCTCCCTCCCCGTCCCGGGTTGGGGCCACCCAGACTGGCCTTTGGC

CACCGCGCCAGCCCTGAACTCTGTTCCCGCCTATCCCCAAGGAGGAAAAGAGGAGGAGAACCGGGGATCC

CAGCCCAGATCGCCTCCCGTCCCGCCCGCAGTGGGGTCGGCGATTCCCGTGCAGCAGAGCCCCCCGCCCA

GTCCGGCCCAGGTGGACACGCTGCAAGCGCGCTACGTGGGGCGACTCACGCAGCTCTTCGAGGAGCACCA

GGCGCGCTATGGTGTCCCCGCCGACAGACACCTGGTCCTCACGGAGGCGCGCCCCACCGCCTGGCCTCGC

CTGTCCGCTGGGTGACTGCAGGTGGGGAAGTGAAATTAAAGACTGGCGGCAGGGCACGGTGGCTCACGCC

TGTGATTCCAGCACTTTGGGAGGCCGAGGCGGGTGGATCACTTGAGGTCAGGAGTTCGAGACCAGCCTGG

CCATCATGGTGAAACCCCATCTCTATTAAAAATCCAAAAAAAAAAAAAAAAAAAAATTAGCCAGACGTGGTG

GCGCGCCTGTAATCCTAGCTACTCGGGAGGCTGAGGCAGGAGAATCGTTTGAACCCGGGAGGCGGAGGT

TGCAGTGAGCCGAGATCGTGCCACTGCTCTCCAGCCTGGGTGACCGAGACGTGTGAAAAAAAAAGAAAAA

AAAGAAAAGAAAAGAAAAAGAAAAAAAATTAAAGTGGGAAACAGACCTGCAGCCTGTGTGTGTTTGTGCAT

GGCGGGGGAGGTCCAGGCAGGGGTGGTGACCTTGACATTCCTGGAGATGCCCCGGACCAGGCCCTGCCCC

CACCCCGACTGGAGGTCAAATAGTCTCAGCAGGGCTTCTGGCCCCTTCCAGCACCTGGGACCCAGTGGGT

GGGGCTAGCCAGATCACTCTTGTCCACTCCAACATCAATTCCTGAGGCACTCGGGTGTCACCTCTCCCTT

GCACACCCCTTACGCCTCCCAGCCCCACCCAGCAGCCAGCCCAGACTGGTGACTGACAGGAAGTTCAAAG

ATCAGGCTGAGAAAAAACCCAGAGACATCTGGGGCTCTGG

>NC_000007.14: mut1

CCCACCATCCTCTTCCCAGGGGACACTGGGGCCCAGCTCTCCTTTCCCCATGTCCTCTCCACTCTTCTTT

CCAAGTCCCAGGCTTTGGCTGGGTGCGGTGGTTCACACCTGTAATCCCAGCACTTGGGAGGCGCAGGCGA

GAGGATCGCTTAAGGCCAGGAGTTCAAGACCAGCCTGGGCAACACAGTGAAACCCCATCTCTACACAAGA

AAAAACAAAAACAAAAATTTAGCCAGGTGTGGTGGTACACACCTGTGGTCCCAGCTACTGGGAGGCTTGA

GGTGGGAGGGTCACTTGAGCCCTGAAGGTCAAGGATGCAGTGAACCAAGATCAGGCCACTGTACTCCAGC

CTGGGCAACAGAGCAAGACTCCGTCCCTAAATAAATAAGTAAATAAGACCCAGGCGTTGGGTCCCCCAGC

TTCTGCCCTCCCCCCAGGTTTGGTCTCCTTTGTCAAGGCCCCGCTGCCTCAGTGGTGGCCAGGTGGCTGT

CCTGGCGTGGGAGGGCCCCTGCAGGCGCTGGAGGCAAAACCCGGACAACTGAGCTTGCCGATTCGGAATC

AGAAGAGATTGGTTAAGTCAGCTCTGGAACTCGGGTGAGGACCCGAGCGTGACCCGGTGGGGACGCCCCA

GGGCTGCCGGGCCGGGGAGGAGGAGAGTGGCGCCACCCGCGGACCCCGGGGCCAGGAGTCAGCCGCGCCA

TGCTCTCCTGCGGCCTCCCTAGTGCCTGTCTTCTCCAGGGAGAATGAGCTCTTCCAGCAGTTCCCGAACC

CGCAGAGCTCGTGGGTGCAGAGGACGCAGGAGGCTCTGCGTCCGCTGCTAAGCGTGGCCCTGCAGCTGTT

CCTGGGCCGCCGGGGCCTCCCGCTGCCCTTCCGCGCGCCCATCCGCACCGTAGGTGAGCCCCGGCCTCCG

CCCCGCGATCCTGGCCCAGGGCCCGCTCCCTCCCCGTCCCGGGTTGGGGCCACCCAGACTGGCCTTTGGC

CACCGCGCCAGCCCTGAACTCTGTTCCCGCCTATCCCCAAGGAGGAAAAGAGGAGGAGAACCGGGGATCC

CAGCCCAGATCGCCTCCCGTCCCGCCCGCAGTGGGGTCGGCGATTCCCGTGCAGCAGAGCCCCCCGCCCA

GTCCGGCCCAGGTGGACACGCTGCAAGCGCGGATGCACCCCCGACTCACGCAGCTCTTCGAGGAGCACCA

GGCGCGCTATGGTGTCCCCGCCGACAGACACCTGGTCCTCACGGAGGCGCGCCCCACCGCCTGGCCTCGC

CTGTCCGCTGGGTGACTGCAGGTGGGGAAGTGAAATTAAAGACTGGCGGCAGGGCACGGTGGCTCACGCC

TGTGATTCCAGCACTTTGGGAGGCCGAGGCGGGTGGATCACTTGAGGTCAGGAGTTCGAGACCAGCCTGG

CCATCATGGTGAAACCCCATCTCTATTAAAAATCCAAAAAAAAAAAAAAAAAAAAATTAGCCAGACGTGGTG

GCGCGCCTGTAATCCTAGCTACTCGGGAGGCTGAGGCAGGAGAATCGTTTGAACCCGGGAGGCGGAGGT

TGCAGTGAGCCGAGATCGTGCCACTGCTCTCCAGCCTGGGTGACCGAGACGTGTGAAAAAAAAAGAAAAA

AAAGAAAAGAAAAGAAAAAGAAAAAAATTAAAGTGGGAAACAGACCTGCAGCCTGTGTGTGTTTGTGCAT

GGCGGGGGAGGTCCAGGCAGGGGTGGTGACCTTGACATTCCTGGAGATGCCCCGGACCAGGCCCTGCCCC

CACCCCGACTGGAGGTCAAATAGTCTCAGCAGGGCTTCTGGCCCCTTCCAGCACCTGGGACCCAGTGGGT

GGGGCTAGCCAGATCACTCTTGTCCACTCCAACATCAATTCCTGAGGCACTCGGGTGTCACCTCTCCCTT

GCACACCCCTTACGCCTCCCAGCCCCACCCAGCAGCCAGCCCAGACTGGTGACTGACAGGAAGTTCAAAG

ATCAGGCTGAGAAAAAACCCAGAGACATCTGGGGCTCTGG

>NC_000007.14 mut2

CCCACCATCCTCTTCCCAGGGGACACTGGGGCCCAGCTCTCCTTTCCCCATGTCCTCTCCACTCTTCTTT

CCAAGTCCCAGGCTTTGGCTGGGTGCGGTGGTTCACACCTGTAATCCCAGCACTTGGGAGGCGCAGGCGA

GAGGATCGCTTAAGGCCAGGAGTTCAAGACCAGCCTGGGCAACACAGTGAAACCCCATCTCTACACAAGA

AAAAACAAAAACAAAAATTTAGCCAGGTGTGGTGGTACACACCTGTGGTCCCAGCTACTGGGAGGCTTGA

GGTGGGAGGGTCACTTGAGCCCTGAAGGTCAAGGATGCAGTGAACCAAGATCAGGCCACTGTACTCCAGC

CTGGGCAACAGAGCAAGACTCCGTCCCTAAATAAATAAGTAAATAAGACCCAGGCGTTGGGTCCCCCAGC

TTCTGCCCTCCCCCCAGGTTTGGTCTCCTTTGTCAAGGCCCCGCTGCCTCAGTGGTGGCCAGGTGGCTGT

CCTGGCGTGGGAGGGCCCCTGCAGGCGCTGGAGGCAAAACCCGGACAACTGAGCTTGCCGATTCGGAATC

AGAAGAGATTGGTTAAGTCAGCTCTGGAACTCGGGTGAGGACCCGAGCGTGACCCGGTGGGGACGCCCCA

GGGCTGCCGGGCCGGGGAGGAGGAGAGTGGCGCCACCCGCGGACCCCGGGGCCAGGAGTCAGCCGCGCCA

TGCTCTCCTGCGGCCTCCCTAGTGCCTGTCTTCTCCAGGGAGAATGAGCTCTTCCAGCAGTTCCCGAACC

CGCAGAGCTCGTGGGTGCAGAGGACGCAGGAGGCTCTGCGTCCGCTGCTAAGCGTGGCCCTGCAGCTGTT

CCTGGGCCGCCGGGGCCTCCCGCTGCCCTTCCGCGCGCCCATCCGCACCGTAGGTGAGCCCCGGCCTCCG

CCCCGCGATCCTGGCCCAGGGCCCGCTCCCTCCCCGTCCCGGGTTGGGGCCACCCAGACTGGCCTTTGGC

CACCGCGCCAGCCCTGAACTCTGTTCCCGCCTATCCCCAAGGAGGAAAAGAGGAGGAGAACCGGGGATCC

CAGCCCAGATCGCCTCCCGTCCCGCCCGCAGTGGGGTCGGCGATTCCCGTGCAGCAGAGCCCCCCGCCCA

GTCCGGCCCAGGTGGACACGCTGCAAGCGCGCTACGTGGGGCGACTCACGCAGCTCTTCGAGGAGCACCA

GGCGCGCTATGGTGTCCCCGCCGACAGACACCTGGTCCTCACGGAGGCGCGCCCCACCGCCTGGCCTCGC

CTGTCCGCTGGGTGACTGCAGGTGGGGAAGTGAAATTAAAGACTGGCGGCAGGGCACGGTGGCTCACGCC

TGTGATTCCAGCACTTTGGGAGGCCGAGGCGGGTGGATCACTTGAGGTCAGGAGTTCGAGACCAGCCTGG

CCATCATGGTGAAACCCCATCTCTATTAAAAATCCAAAAAAAAAAAAAAAAAAAATTAGCCTCTGCACCACGCG

CGCCTGTAATCCTAGCTACTCGGGAGGCTGAGGCAGGAGAATCGTTTGAACCCGGGAGGCGGAGGT

TGCAGTGAGCCGAGATCGTGCCACTGCTCTCCAGCCTGGGTGACCGAGACGTGTGAAAAAAAAAGAAAAA

AAAGAAAAGAAAAGAAAAAGAAAAAAATTAAAGTGGGAAACAGACCTGCAGCCTGTGTGTGTTTGTGCAT

GGCGGGGGAGGTCCAGGCAGGGGTGGTGACCTTGACATTCCTGGAGATGCCCCGGACCAGGCCCTGCCCC

CACCCCGACTGGAGGTCAAATAGTCTCAGCAGGGCTTCTGGCCCCTTCCAGCACCTGGGACCCAGTGGGT

GGGGCTAGCCAGATCACTCTTGTCCACTCCAACATCAATTCCTGAGGCACTCGGGTGTCACCTCTCCCTT

GCACACCCCTTACGCCTCCCAGCCCCACCCAGCAGCCAGCCCAGACTGGTGACTGACAGGAAGTTCAAAG

ATCAGGCTGAGAAAAAACCCAGAGACATCTGGGGCTCTGG

>NC_000007.14 mut3

CCCACCATCCTCTTCCCAGGGGACACTGGGGCCCAGCTCTCCTTTCCCCATGTCCTCTCCACTCTTCTTT

CCAAGTCCCAGGCTTTGGCTGGGTGCGGTGGTTCACACCTGTAATCCCAGCACTTGGGAGGCGCAGGCGA

GAGGATCGCTTAAGGCCAGGAGTTCAAGACCAGCCTGGGCAACACAGTGAAACCCCATCTCTACACAAGA

AAAAACAAAAACAAAAATTTAGCCAGGTGTGGTGGTACACACCTGTGGTCCCAGCTACTGGGAGGCTTGA

GGTGGGAGGGTCACTTGAGCCCTGAAGGTCAAGGATGCAGTGAACCAAGATCAGGCCACTGTACTCCAGC

CTGGGCAACAGAGCAAGACTCCGTCCCTAAATAAATAAGTAAATAAGACCCAGGCGTTGGGTCCCCCAGC

TTCTGCCCTCCCCCCAGGTTTGGTCTCCTTTGTCAAGGCCCCGCTGCCTCAGTGGTGGCCAGGTGGCTGT

CCTGGCGTGGGAGGGCCCCTGCAGGCGCTGGAGGCAAAACCCGGACAACTGAGCTTGCCGATTCGGAATC

AGAAGAGATTGGTTAAGTCAGCTCTGGAACTCGGGTGAGGACCCGAGCGTGACCCGGTGGGGACGCCCCA

GGGCTGCCGGGCCGGGGAGGAGGAGAGTGGCGCCACCCGCGGACCCCGGGGCCAGGAGTCAGCCGCGCCA

TGCTCTCCTGCGGCCTCCCTAGTGCCTGTCTTCTCCAGGGAGAATGAGCTCTTCCAGCAGTTCCCGAACC

CGCAGAGCTCGTGGGTGCAGAGGACGCAGGAGGCTCTGCGTCCGCTGCTAAGCGTGGCCCTGCAGCTGTT

CCTGGGCCGCCGGGGCCTCCCGCTGCCCTTCCGCGCGCCCATCCGCACCGTAGGTGAGCCCCGGCCTCCG

CCCCGCGATCCTGGCCCAGGGCCCGCTCCCTCCCCGTCCCGGGTTGGGGCCACCCAGACTGGCCTTTGGC

CACCGCGCCAGCCCTGAACTCTGTTCCCGCCTATCCCCAAGGAGGAAAAGAGGAGGAGAACCGGGGATCC

CAGCCCAGATCGCCTCCCGTCCCGCCCGCAGTGGGGTCGGCGATTCCCGTGCAGCAGAGCCCCCCGCCCA

GTCCGGCCCAGGTGGACACGCTGCAAGCGCGCTACGTGGGGCGACTCACGCAGCTCTTCGAGGAGCACCA

GGCGCGCTATGGTGTCCCCGCCGACAGACACCTGGTCCTCACGGAGGCGCGCCCCACCGCCTGGCCTCGC

CTGTCCGCTGGGTGACTGCAGGTGGGGAAGTGAAATTAAAGACTGGCGGCAGGGCACGGTGGCTCACGCC

TGTGATTCCAGCACTTTGGGAGGCCGAGGCGGGTGGATCACTTGAGGTCAGGAGTTCGAGACCAGCCTGG

CCATCATGGTGAAACCCCATCTCTATTAAAAATCCAAAAAAAAAAAAAAAAAAAAATTAGCCAGACGTGGTG

GCGCGCCTGTAATCCTAGCTACTCGGGAGGCTGAGGCAGGAGAATCGTTTGAACCCGGGAGGCGGAGGT

TGCAGTGAGCCGAGATCGTGCCACTGCTCTCCAGCCTGGGTGACCGTCTGCACACTAAAAAAAAGAAAAA

AAAGAAAAGAAAAGAAAAAGAAAAAAAATTAAAGTGGGAAACAGACCTGCAGCCTGTGTGTGTTTGTGCAT

GGCGGGGGAGGTCCAGGCAGGGGTGGTGACCTTGACATTCCTGGAGATGCCCCGGACCAGGCCCTGCCCC

CACCCCGACTGGAGGTCAAATAGTCTCAGCAGGGCTTCTGGCCCCTTCCAGCACCTGGGACCCAGTGGGT

GGGGCTAGCCAGATCACTCTTGTCCACTCCAACATCAATTCCTGAGGCACTCGGGTGTCACCTCTCCCTT

GCACACCCCTTACGCCTCCCAGCCCCACCCAGCAGCCAGCCCAGACTGGTGACTGACAGGAAGTTCAAAG

ATCAGGCTGAGAAAAAACCCAGAGACATCTGGGGCTCTGG
