# Supplementary material for: MOGAT3-mediated DAG accumulation drives acquired resistance to anti-BRAF/anti-EGFR therapy in BRAFV600E-mutant metastatic colorectal cancer
Source: J Clin Invest. 2024 Oct 22;134(24):e182217. doi: 10.1172/JCI182217 (PMC11645146; doi:10.1172/JCI182217)

Full unedited gel for Figure 3 and Figure S3

Fig.3A

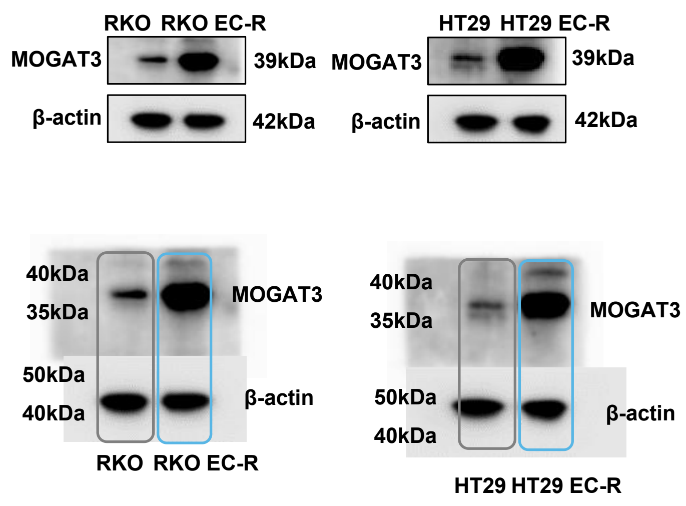

Fig.3B

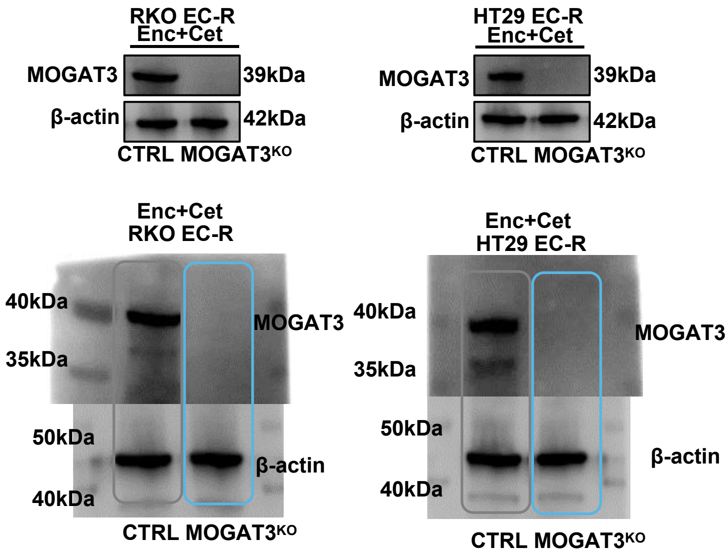

Fig.S3C

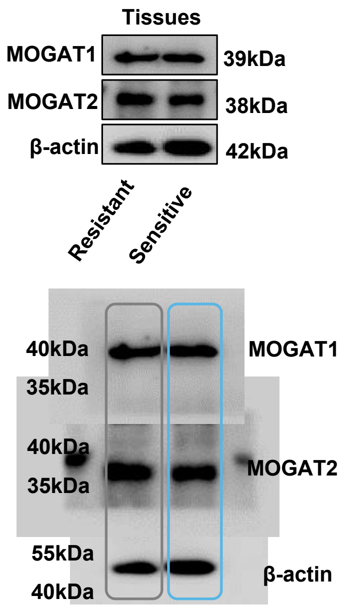

Fig.S3F

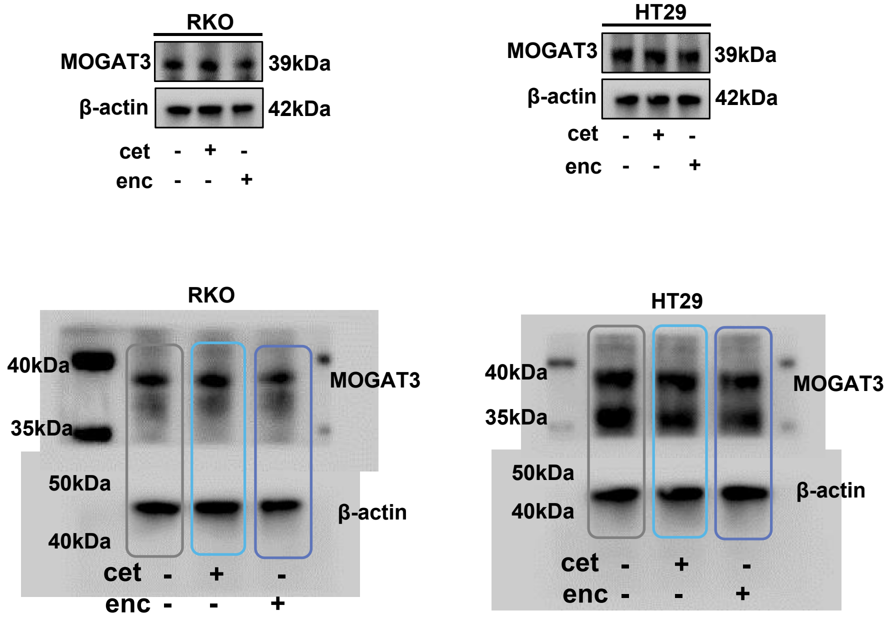

Fig.S3G

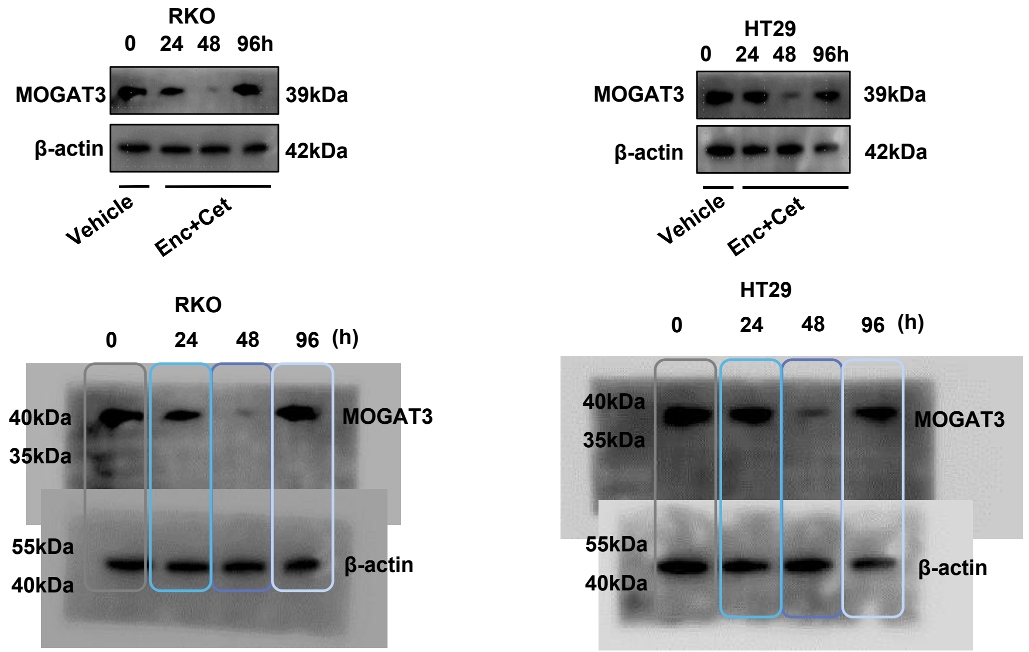

Full unedited gel for Figure S3

Fig.S3I

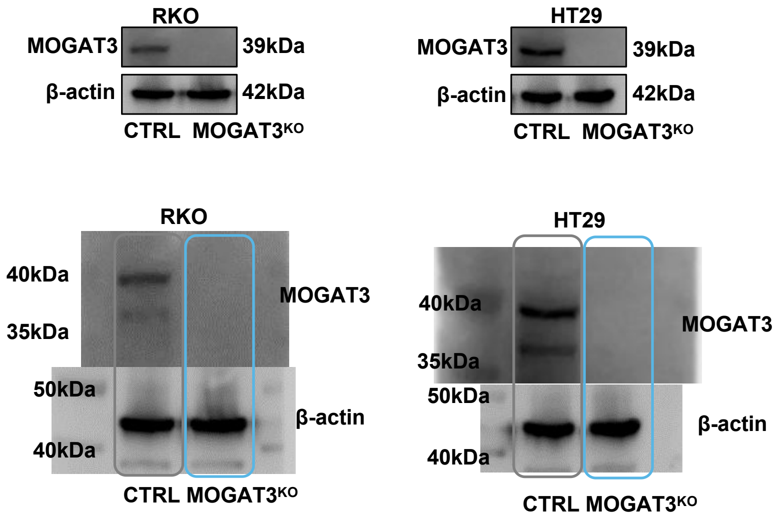

Fig.S3Q

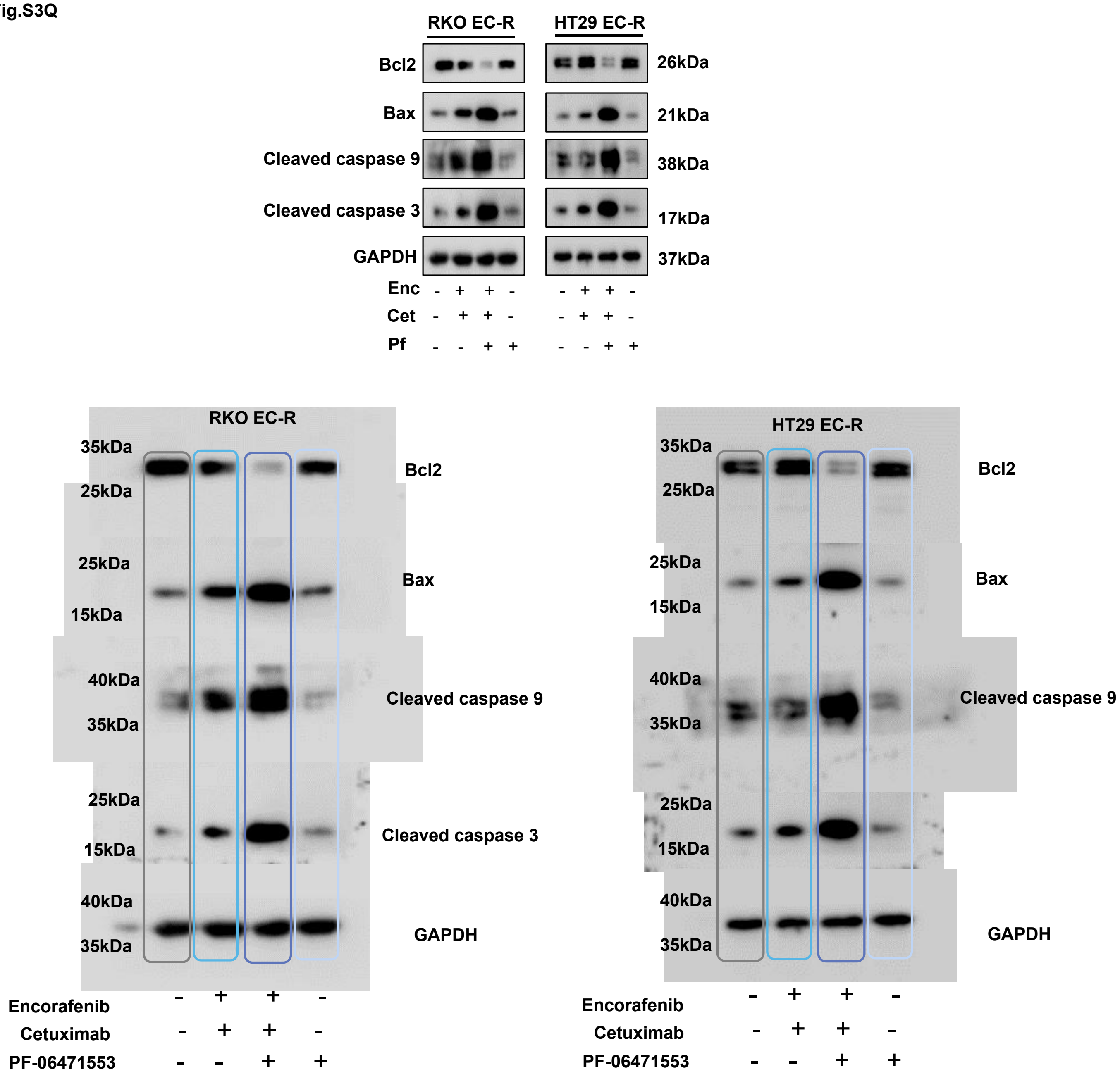

Full unedited gel for Figure 4 and Figure S4

Fig.4A

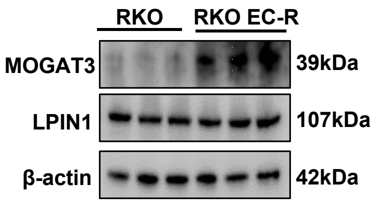

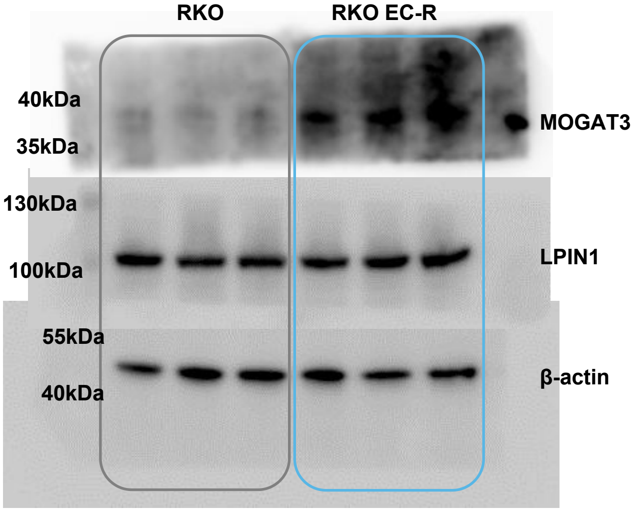

Fig.S4A

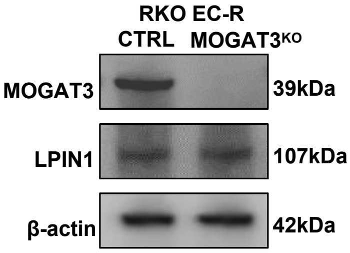

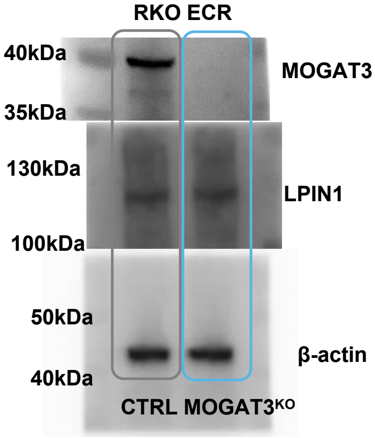

Full unedited gel for Figure 5

Fig5A

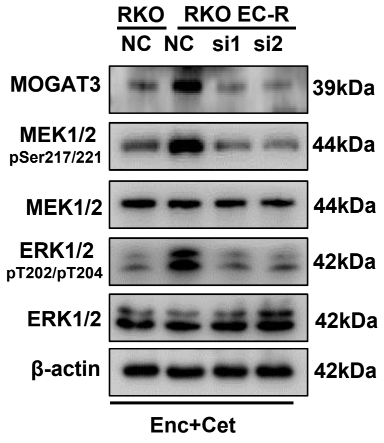

Fig.5B.

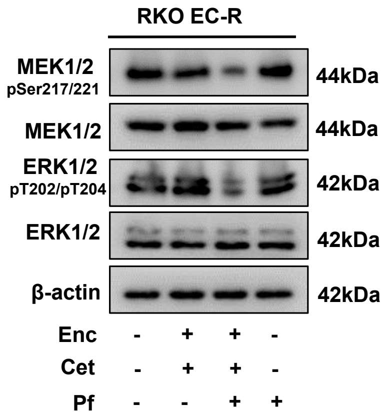

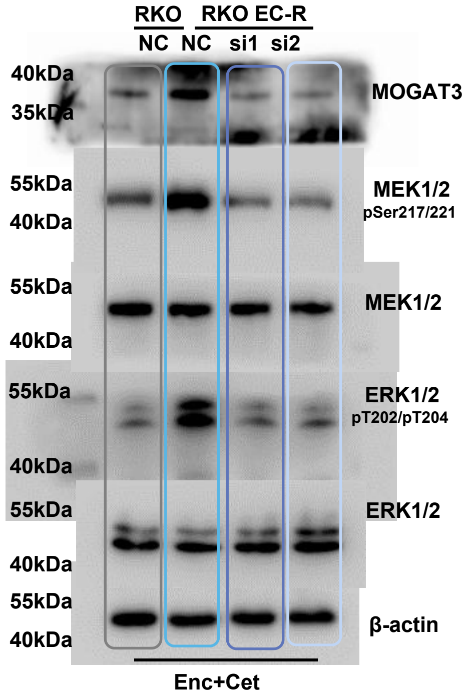

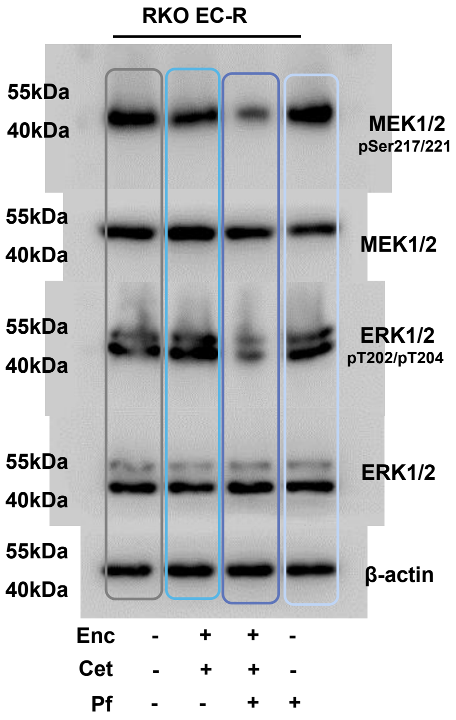

Full unedited gel for Figure 5

Fig5C

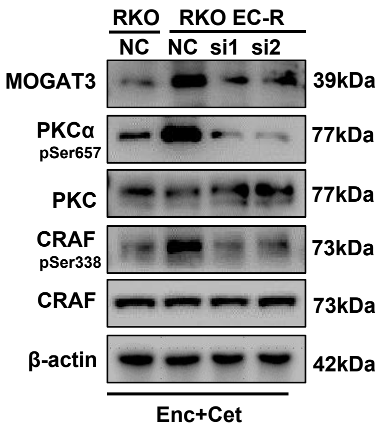

Fig.5E

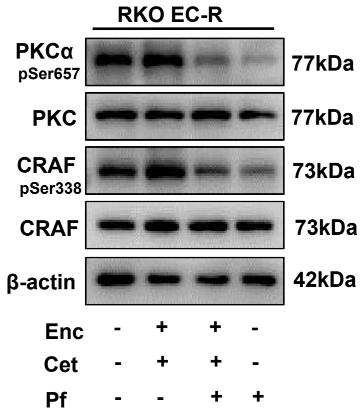

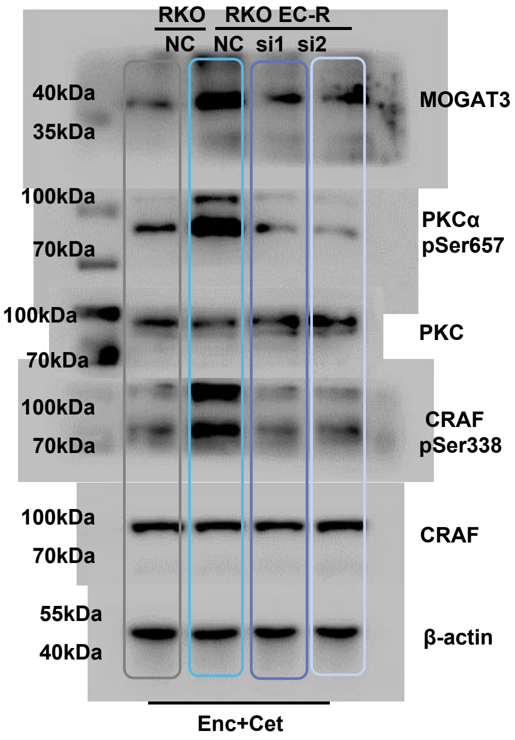

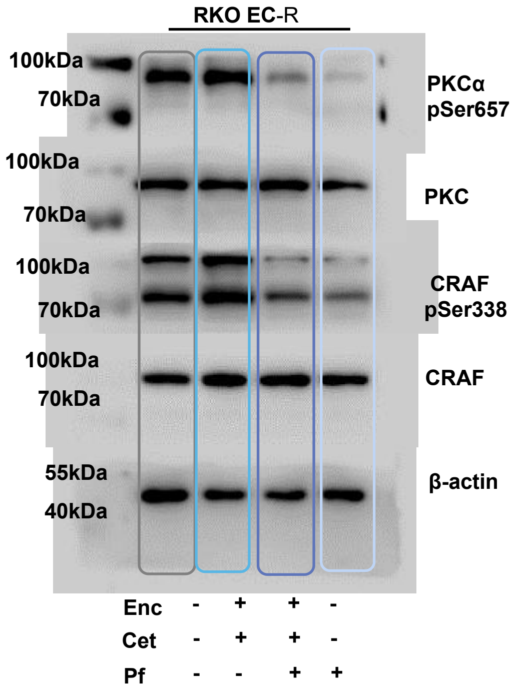

Full unedited gel for Figure 5

Fig.5F.

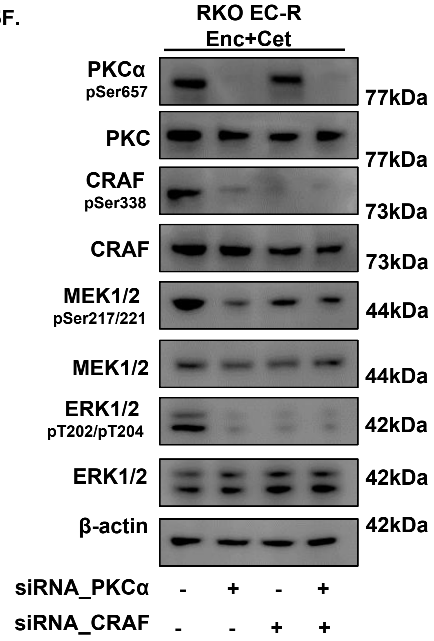

Fig.5G.

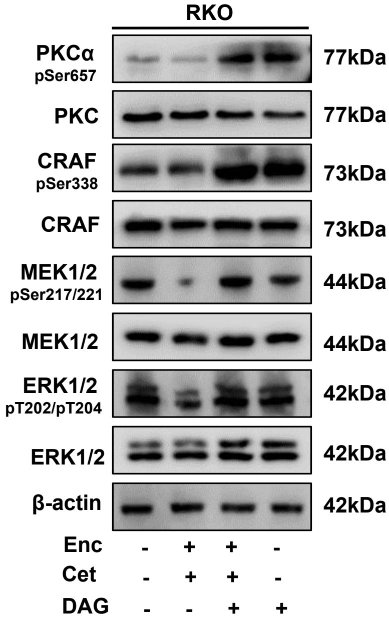

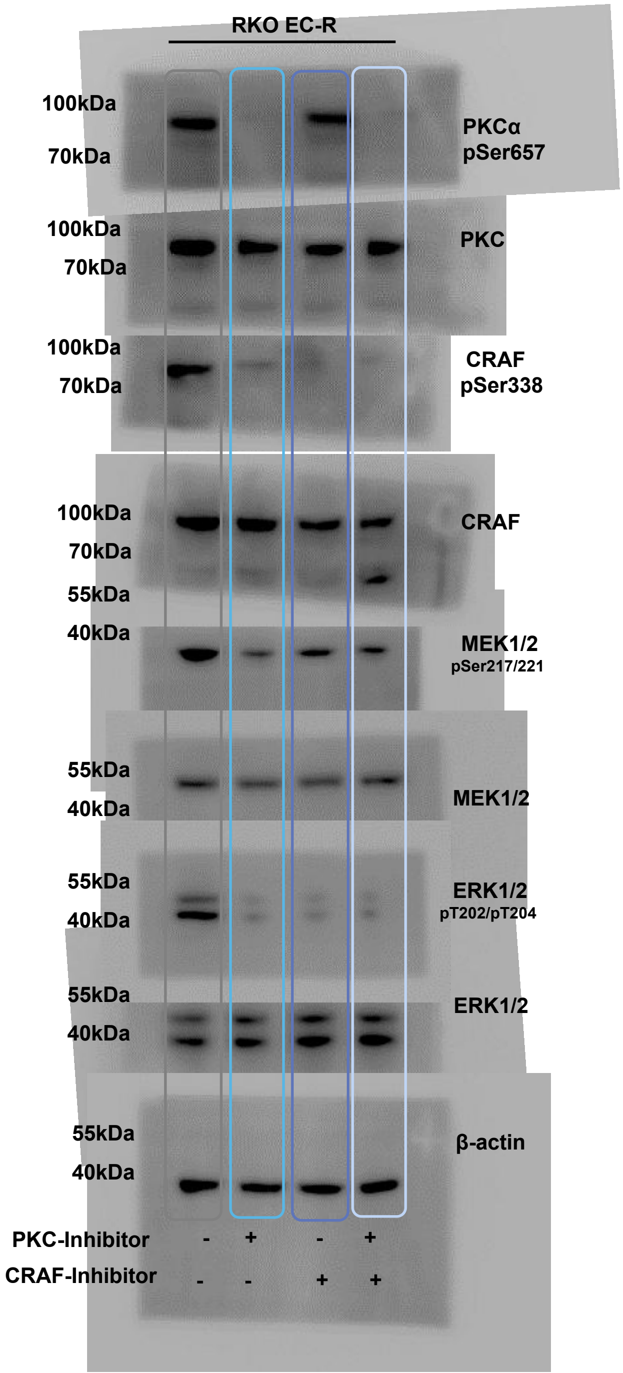

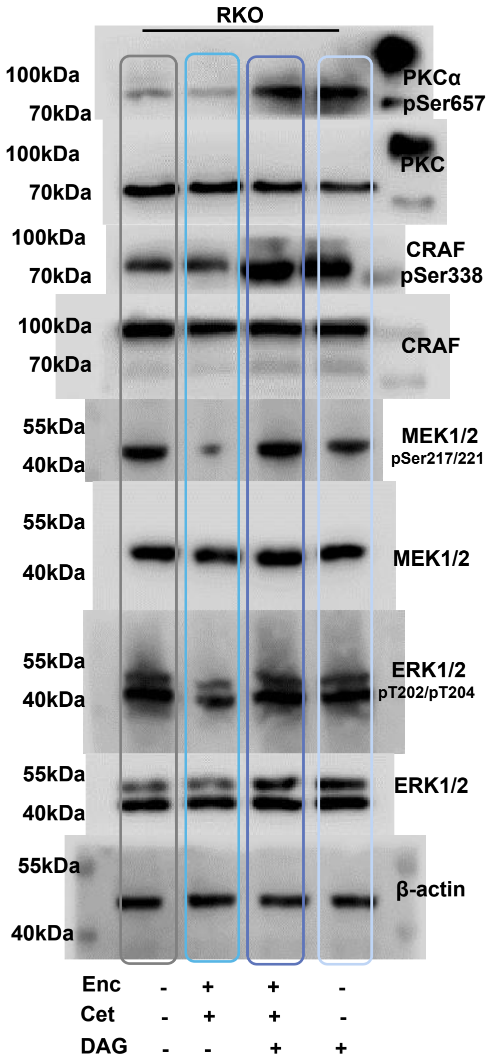

Full unedited gel for Figure 5 and Figure S5

Fig.5H

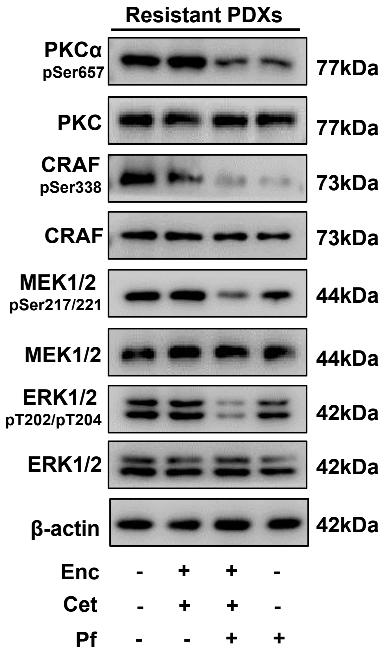

Fig.S5E

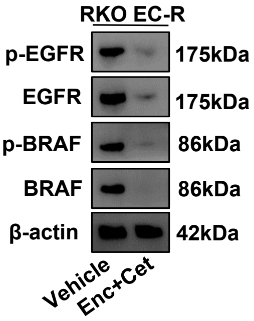

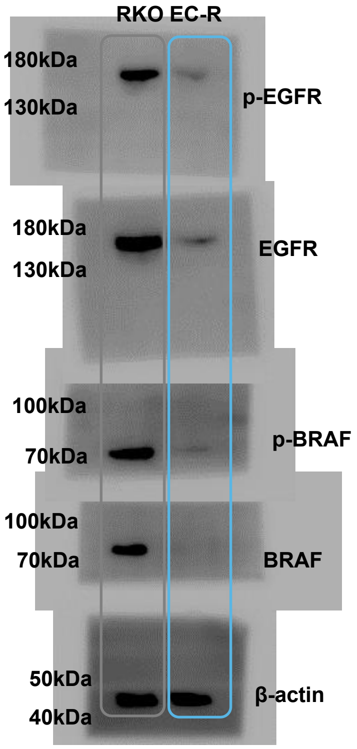

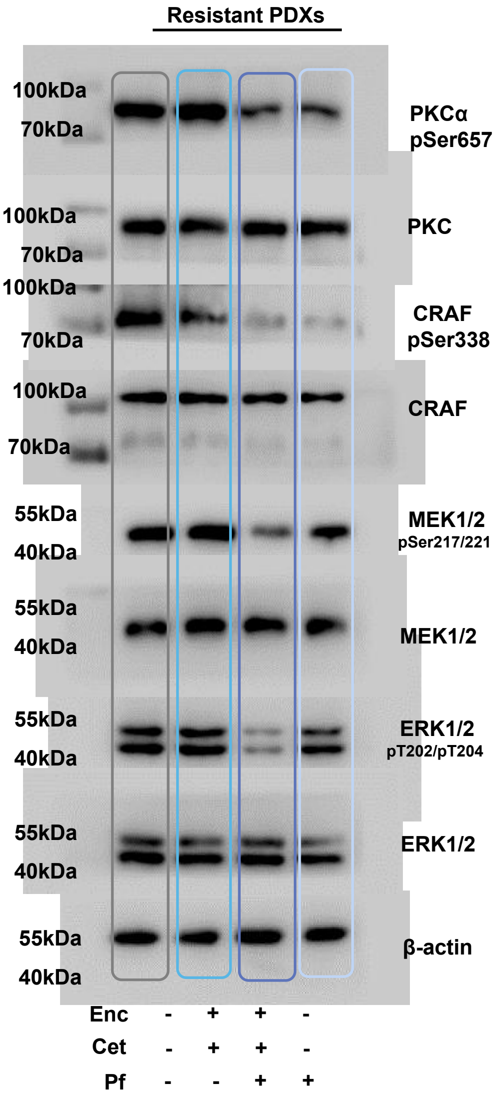

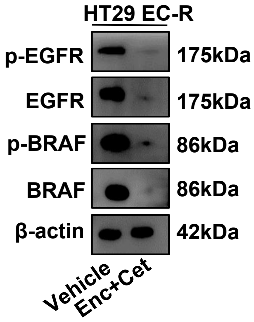

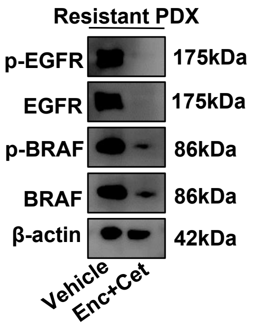

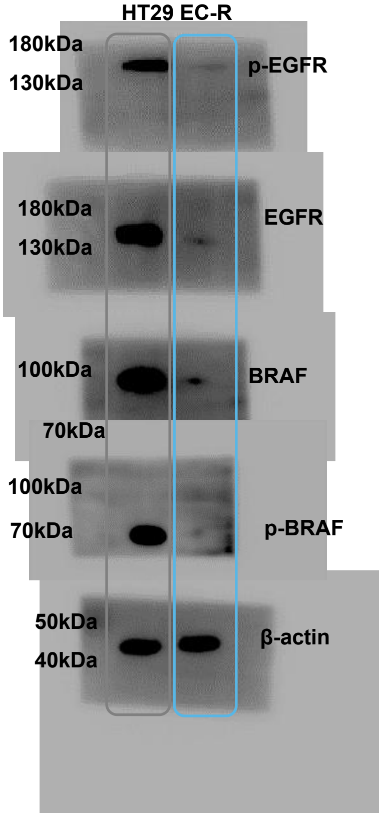

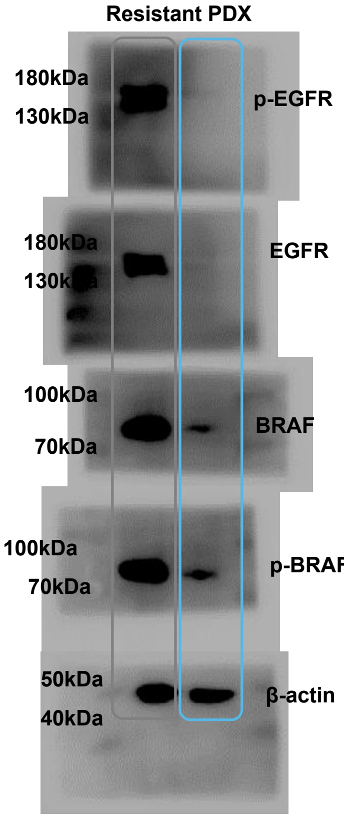

Full unedited gel for Figure S5

Fig.S5G

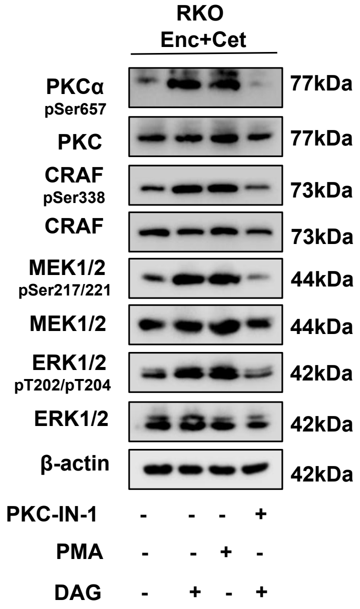

Fig.S5H

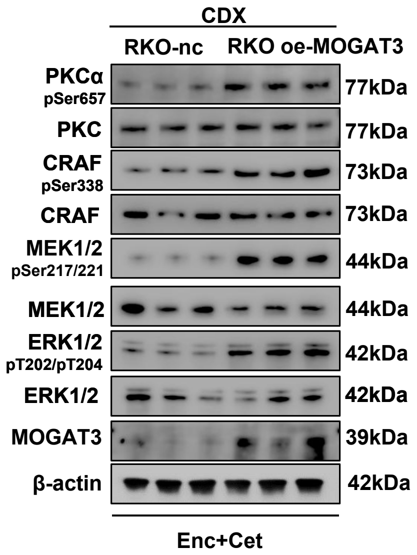

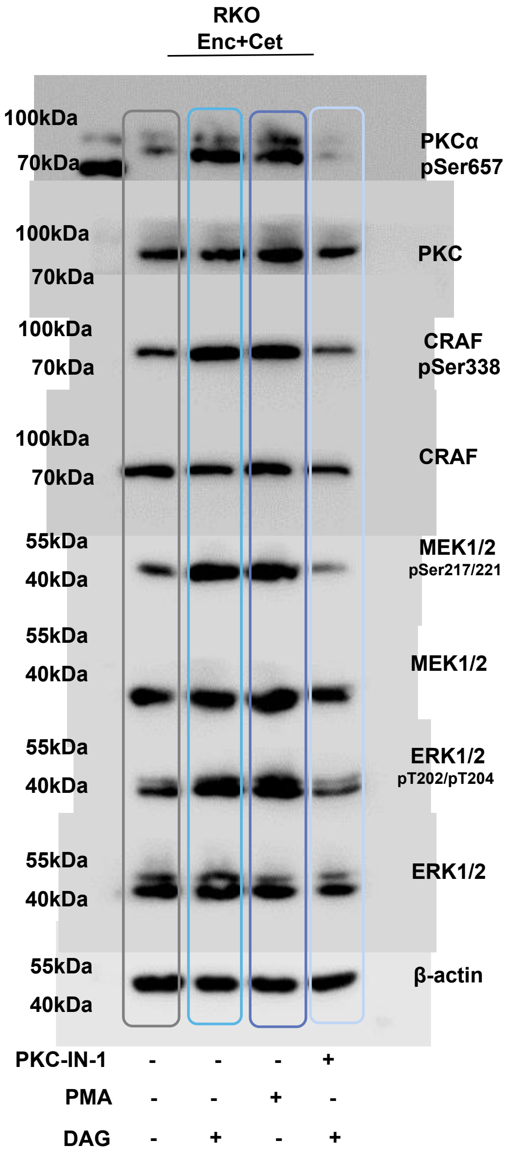

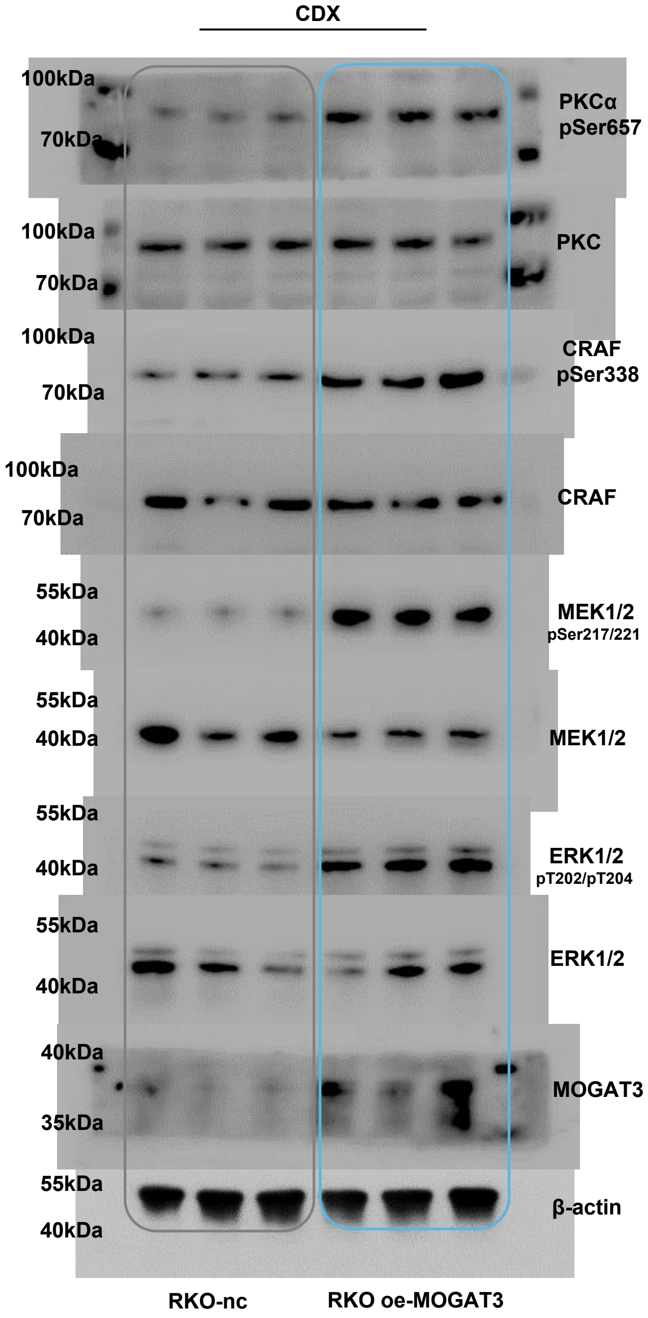

Full unedited gel for Figure 6

Fig.6B

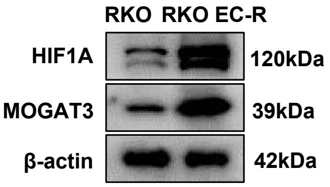

Fig.6C

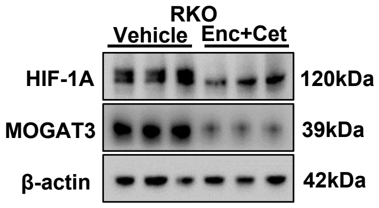

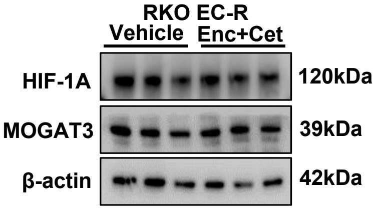

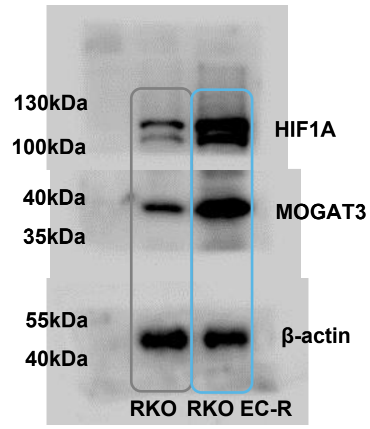

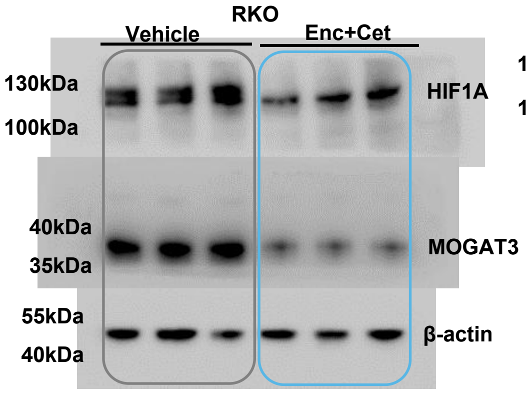

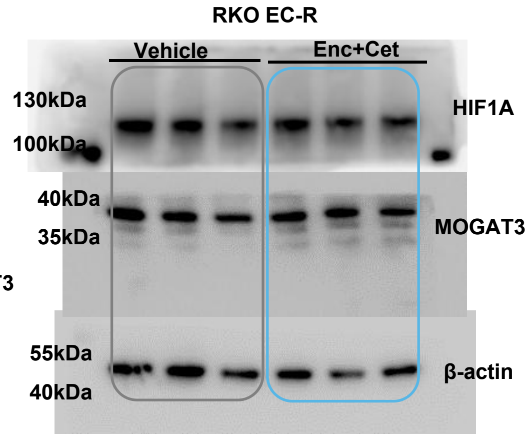

Fig.6D

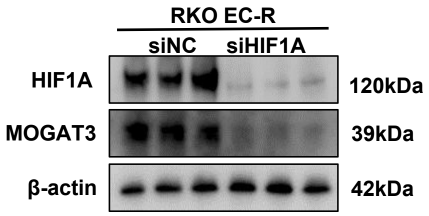

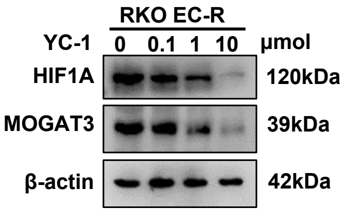

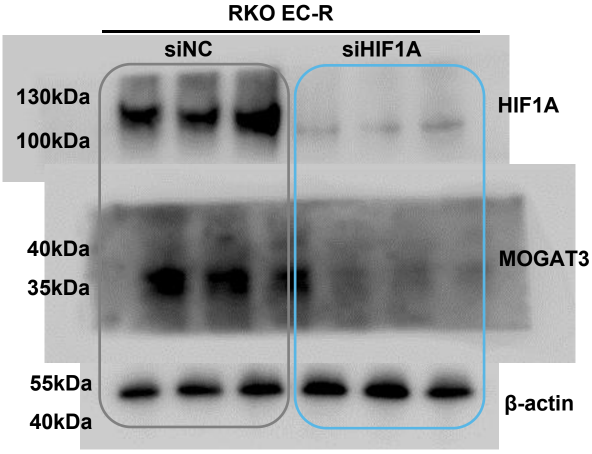

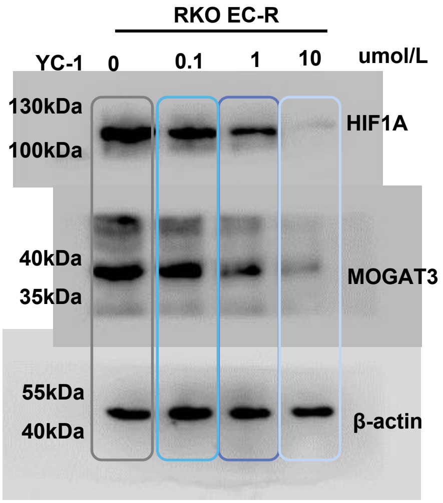

Full unedited gel for Figure 6

Fig.6E

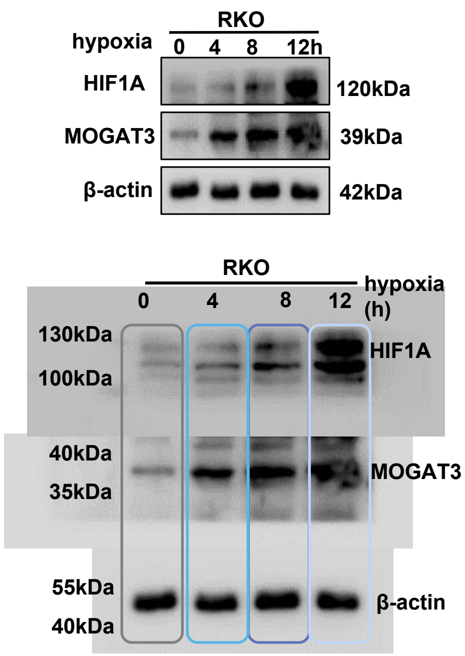

Fig.6I

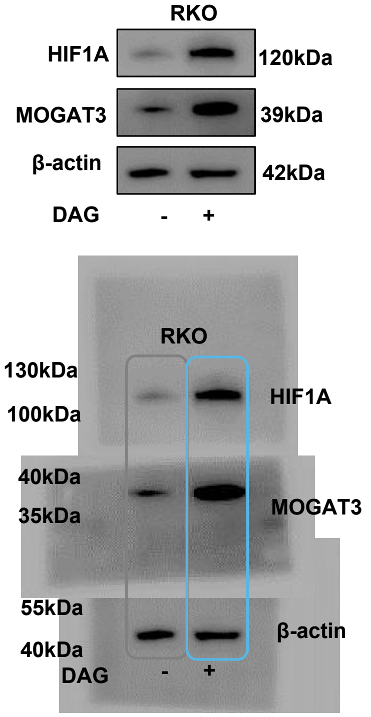

Fig.6J

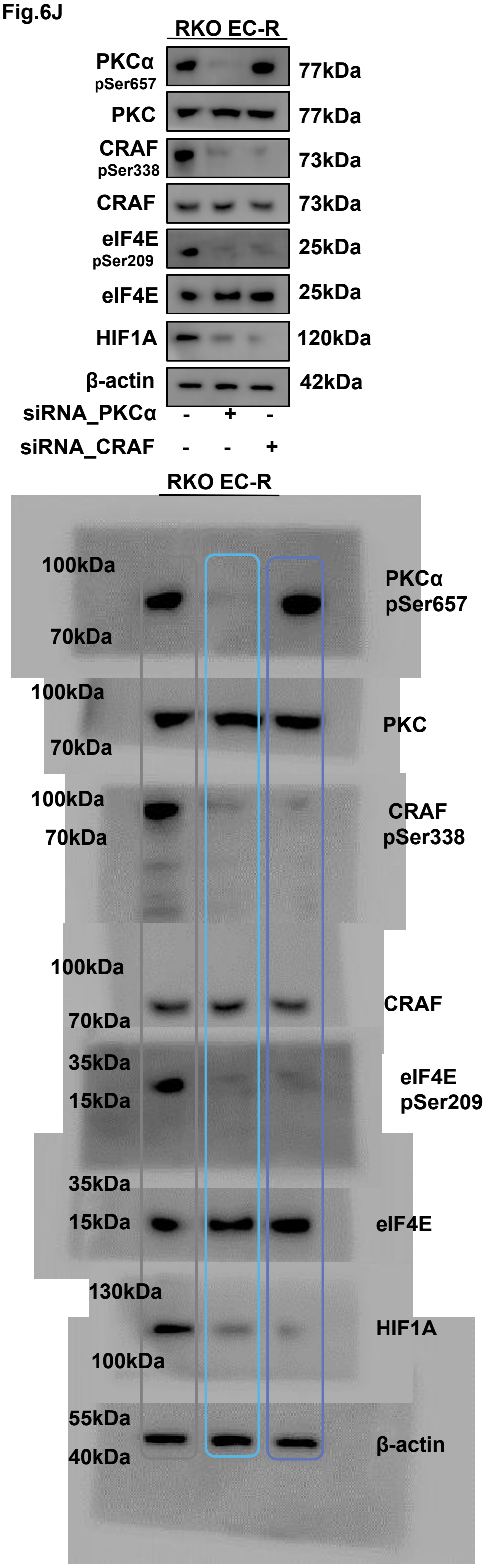

Fig.6K

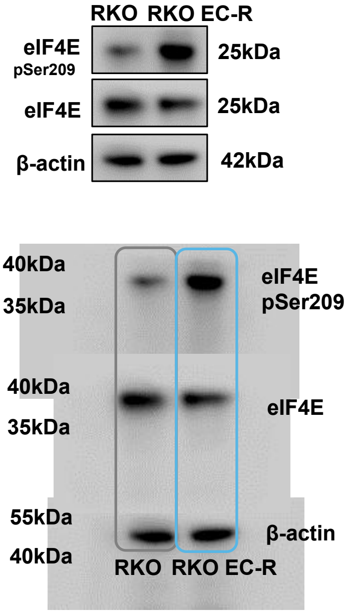

Fig.6L

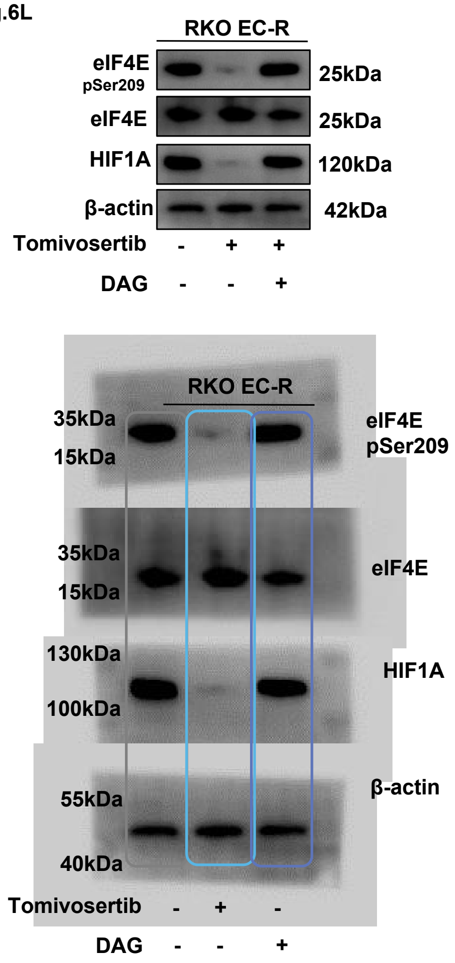

Full unedited gel for Figure 6and Figure S6

Fig.6M

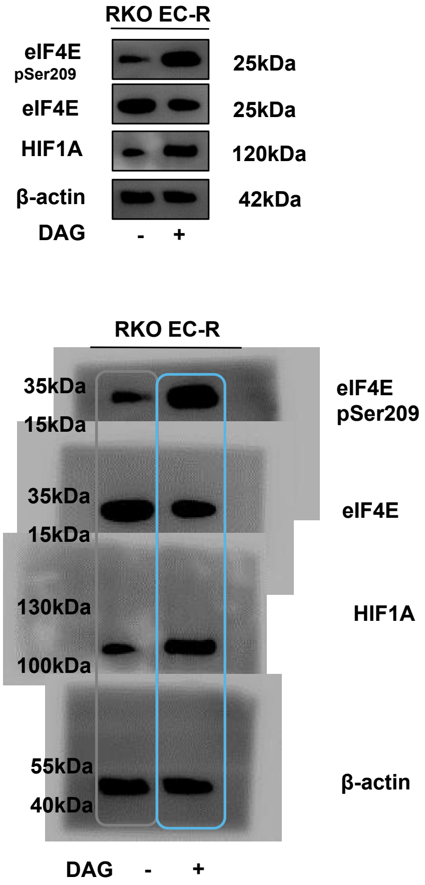

Fig.S6B

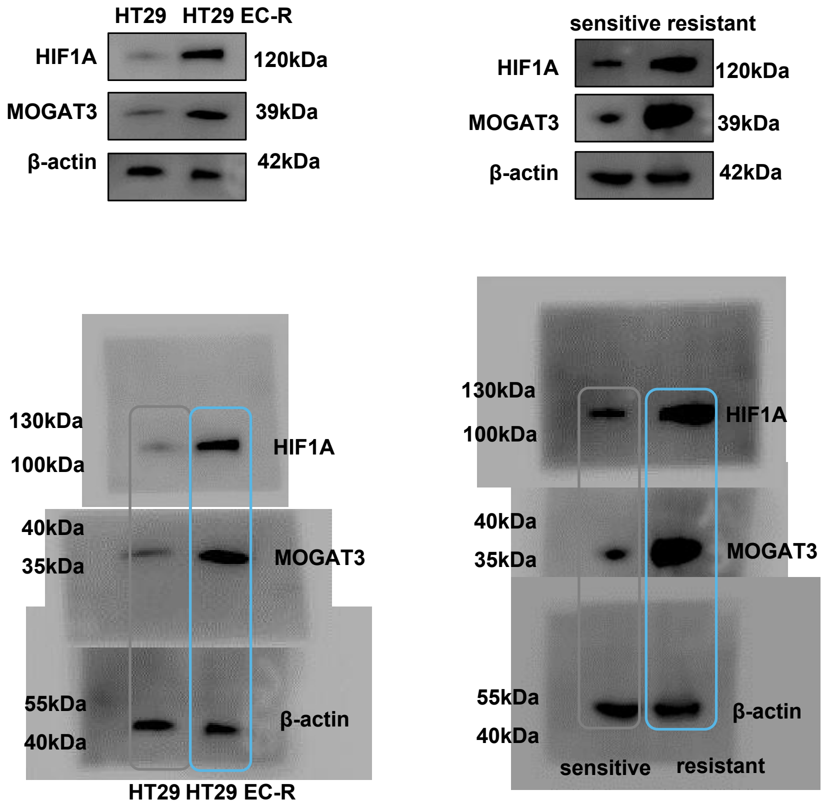

Fig.S6E

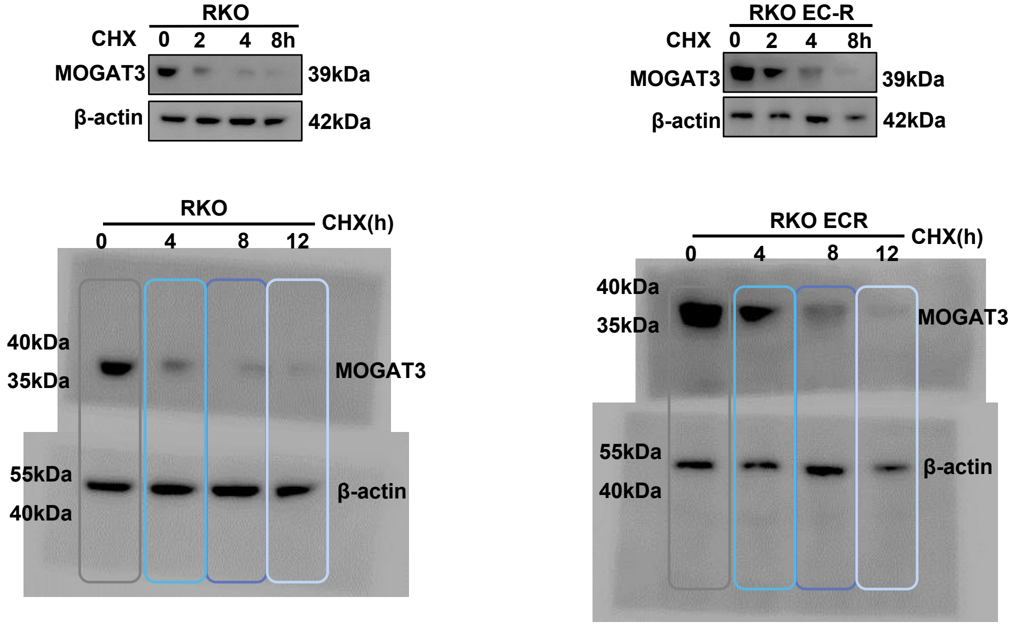

Full unedited gel for Figure S6

Fig.S6G

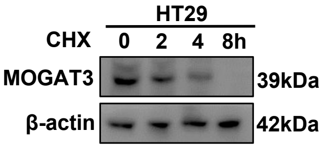

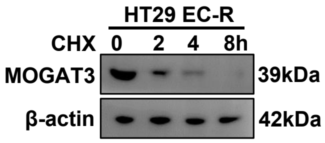

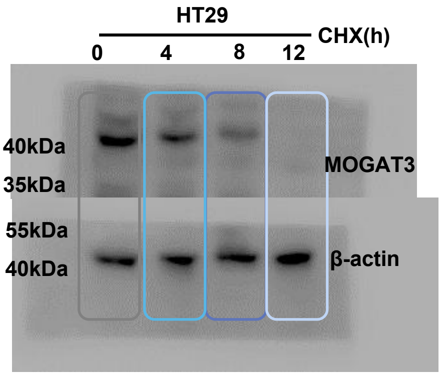

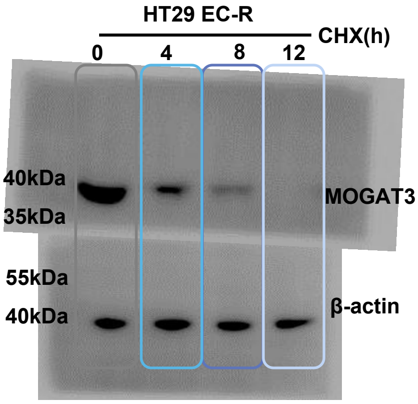

Fig.S6I

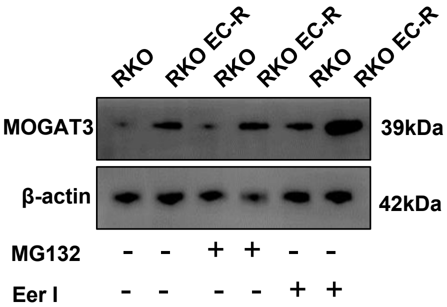

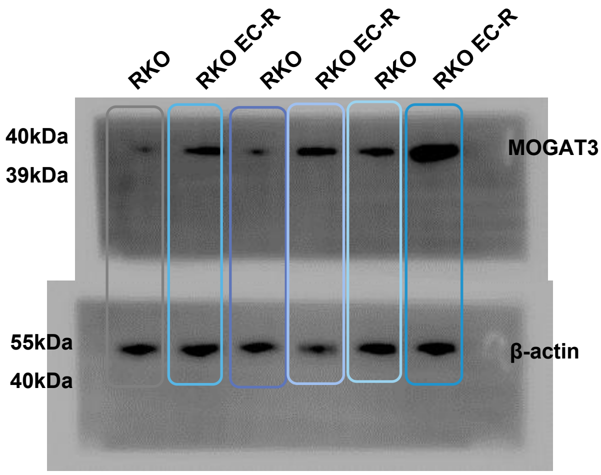

Fig.S6J

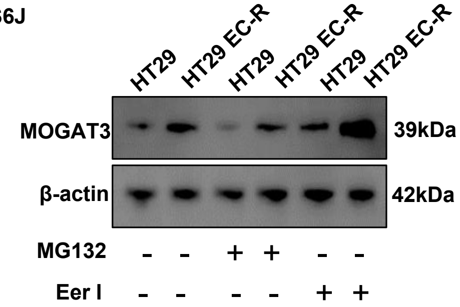

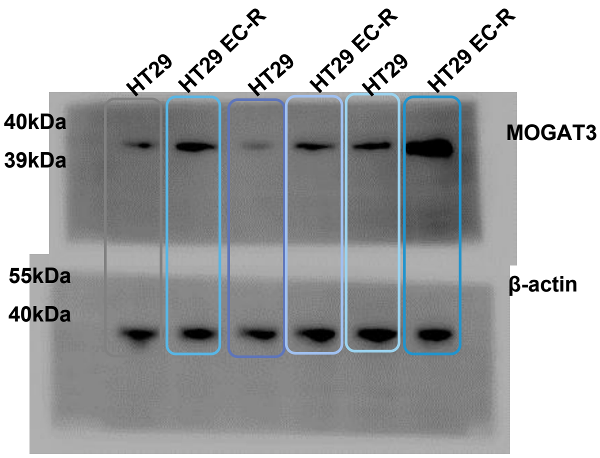

Full unedited gel for Figure 7

Fig.7F

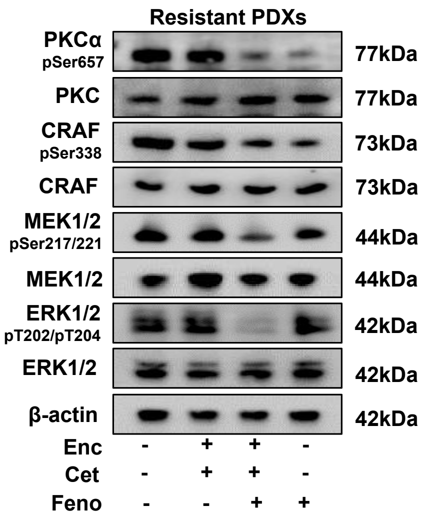

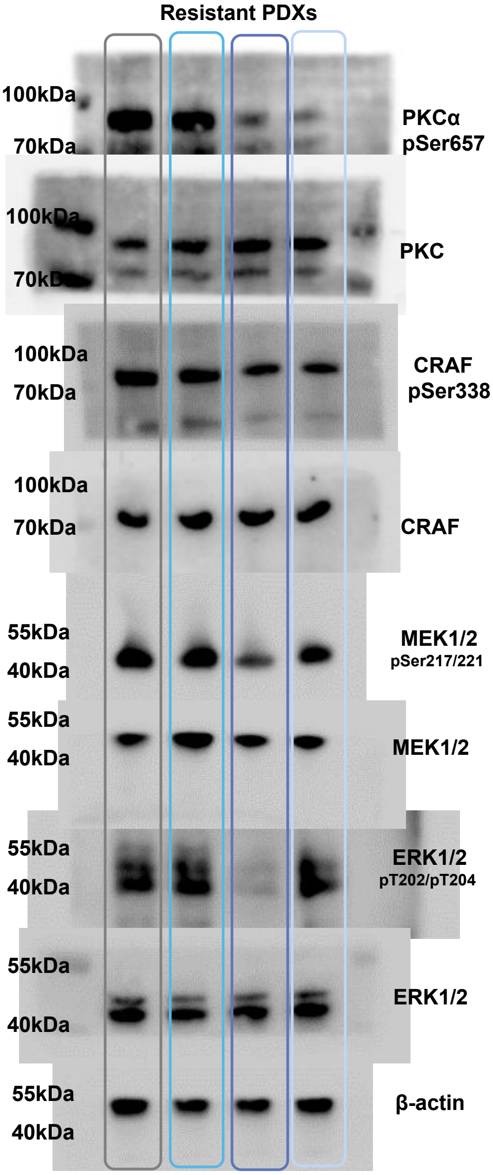

Fig.7I

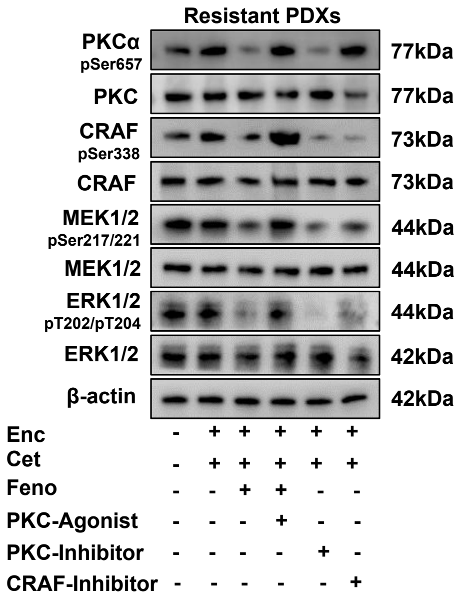

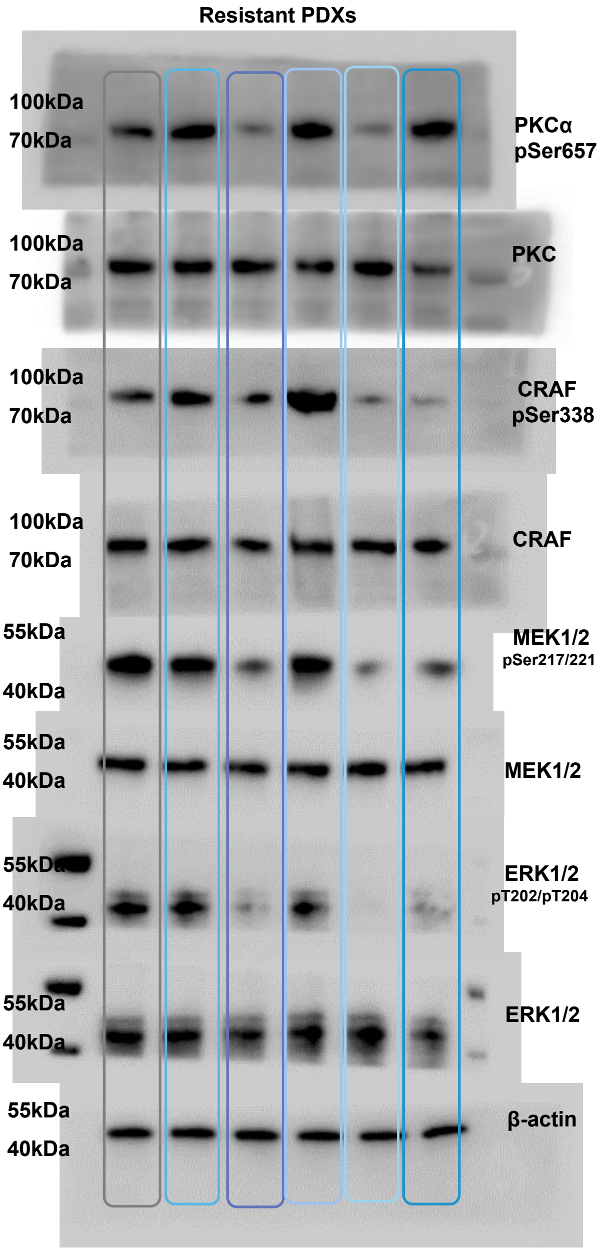

Full unedited gel for FigureS7

Fig.S7B

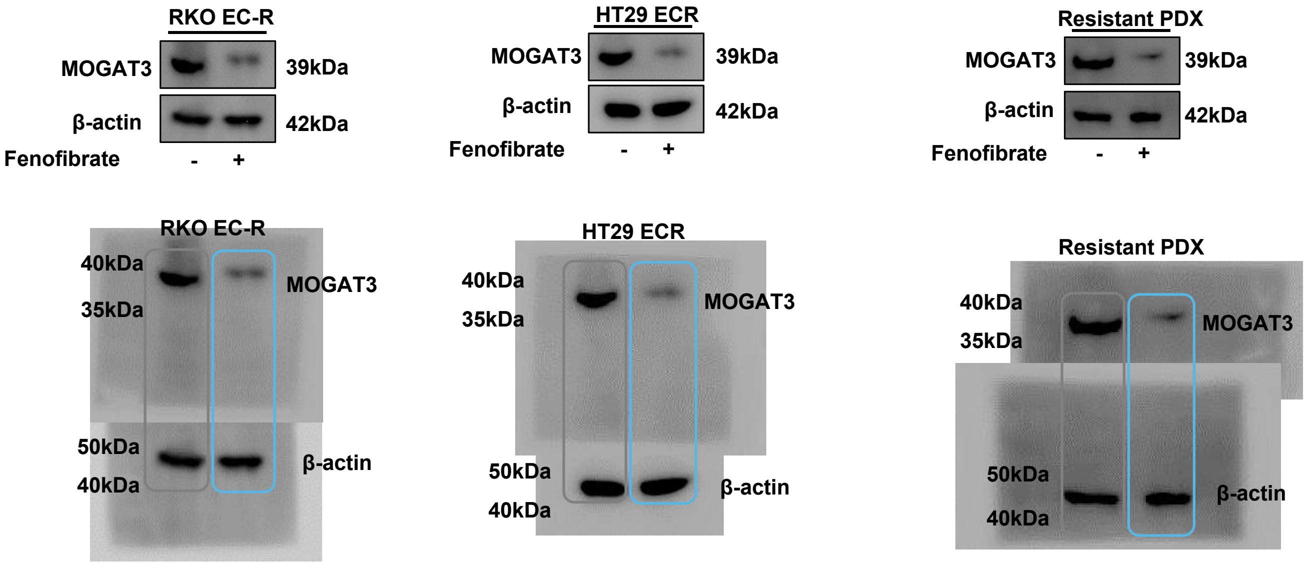

Supplement: Unedited blot and gel images [file jci-134-182217-s086.pdf]
